# Origin of the Ligand Ring‐Size Effect on the Catalytic Activity of Cationic Calcium Hydride Dimers in the Hydrogenation of Unactivated 1‐Alkenes

**DOI:** 10.1002/open.202200240

**Published:** 2022-12-16

**Authors:** Hui Zhu, Zheng‐Wang Qu, Stefan Grimme

**Affiliations:** ^1^ Mulliken Center for Theoretical Chemistry University of Bonn Beringstr. 4 53115 Bonn Germany

**Keywords:** 1-alkene, calcium hydride complexes, homogenous catalysis, hydrogenation, isotope exchange

## Abstract

Recently, it was shown that the double Ca−H−Ca‐bridged calcium hydride cation dimer [LCaH_2_CaL]^2+^ when stabilized by a larger macrocyclic N,N’,N’’,N’’’,N’’’’‐pentadentate ligand showed evidently higher activity than when stabilized by a smaller N,N’,N’’,N’’’‐tetradentate ligand in the catalytic hydrogenation of unactivated 1‐alkenes. In this DFT‐mechanistic work, the origin of the observed ring‐size effect is examined in detail using 1‐hexene, CH_2_=CH_2_ and H_2_ as substrates. It is shown that, at room temperature, both the N,N’,N’’,N’’’,N’’’’‐stabilized dimer and the monomer are not coordinated by THF in solution, while the corresponding N,N’,N’’,N’’’‐stabilized structures are coordinated by one THF molecule mimicking the fifth N‐coordination. Catalytic 1‐alkene hydrogenation may occur via *anti*‐Markovnikov addition over the terminal Ca−H bonds of transient monomers, followed by faster Ca−C bond hydrogenolysis. The higher catalytic activity of the larger N,N’,N’’,N’’’,N’’’’‐stabilized dimer is due to not only easier formation of but also due to the higher reactivity of the catalytic monomeric species. In contrast, despite unfavorable THF‐coordination in solution, the smaller N,N’,N’’,N’’’‐stabilized dimer shows a 3.2 kcal mol^−1^ lower barrier via a dinuclear cooperative Ca−H−Ca bridge for H_2_ isotope exchange than the large N,N’,N’’,N’’’,N’’’’‐stabilized dimer, mainly due to less steric hindrance. The observed ring‐size effect can be understood mainly by a subtle interplay of solvent, steric and cooperative effects that can be resolved in detail by state‐of‐the‐art quantum chemistry calculations.

## Graphical abstract

**The catalytic activity of double Ca−H−Ca‐bridged calcium hydride cation dimer complexes** is influenced by using macrocyclic tetra‐ or pentadentate ligands. Extensive dispersion‐corrected DFT calculations disclose that this influence is due to a subtle balance of solvent, steric and dinuclear cooperative effects that also depend on the substrate.

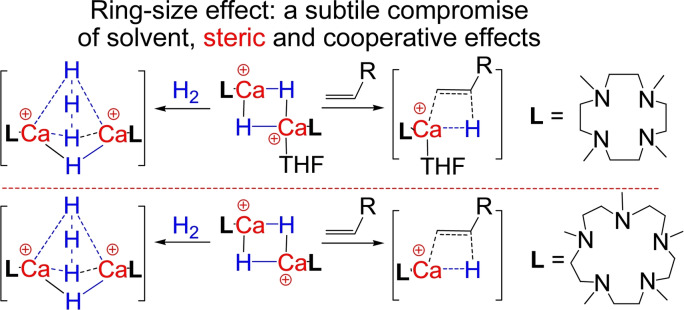

## Introduction

**Scheme 1:**
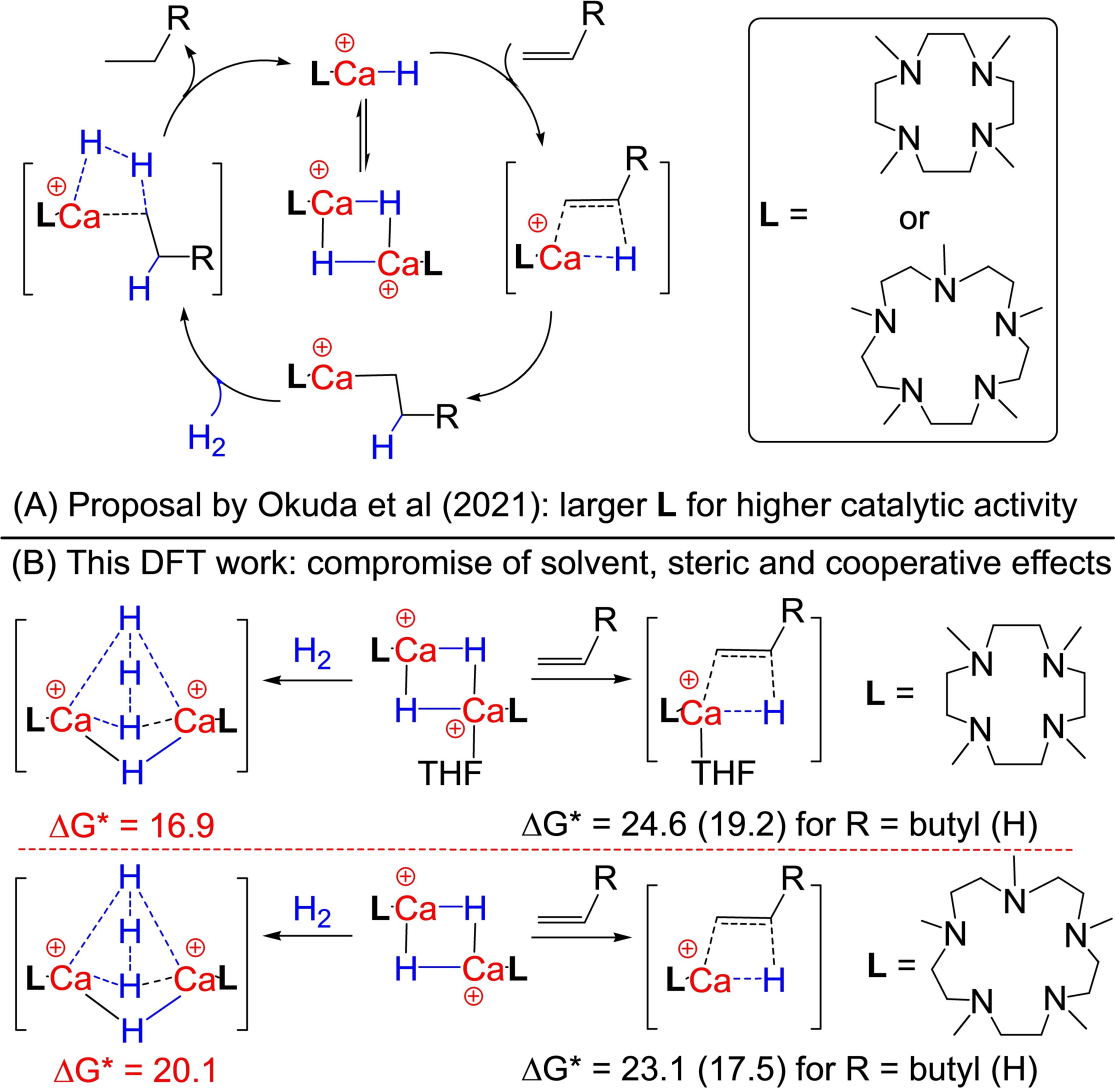
(A) Recently proposed ligand ring‐size effect for the catalytic activity of calcium hydride cation dimer catalysts; (B) subtle interplay of solvent, steric and dinuclear cooperative effects via competitive Ca−H−Ca bridges and terminal Ca−H bonds, as found in this DFT‐mechanistic work.

Heavier alkaline‐earth metals Ca, Sr and Ba (especially the abundant and low‐toxic calcium)^[1]^ have been successfully applied to a variety of catalytic hydroelementation and hydrogenation reactions that partially mimic^[2]^ the well‐developed transition metal catalysis.^[3]^ Usually, “frustrated” Lewis pair (FLP)^[4]^ and main‐group‐metal[^1^, ^5^] hydrogenation catalysts are limited to conjugation‐activated alkene substrates. Rare examples of hydrogenation catalysts for unactivated alkene substrates include the borane HB(C_6_F_5_)_2_ under elevated heating,^[6]^ the neutral calcium hydride monomer TpCaH (Tp=hydrotris(3‐adamantyl‐5‐isopropyl‐pyrazolyl)borate, a super‐bulky N,N’,N’’‐type ligand),^[7]^ the non‐THF‐coordinated calcium hydride dimer [(BDI)CaH_2_Ca(BDI)] (ligand BDI=HC[(Me)CN‐2,6‐*i*‐Pr_2_C_6_H_3_]_2_,^[8]^ and the double Ca−H−Ca‐bridged calcium hydride cation dimer **1^2+^** [LCaH_2_CaL]^2+^ (L=1,4,7,10‐tetramethyl‐1,4,7,10‐tetraazacyclododecane, N,N’,N’’,N’’’‐tetradentate L4)^[9]^ with a coordinating THF.^[10]^ More recently, Okuda et al. showed that the use of a larger N,N’,N’’,N’’’,N’’’’‐pentadentate macrocyclic ligand in the similar cation dimer **2^2+^** (L=1,4,7,10,13‐pentamethyl‐1,4,7,10,13‐pentaazacyclopentadecane, L5) can evidently improve such catalytic activity, due to easier monomer formation from dimer catalysts when stabilized by larger L5 ligands (Scheme 1A).^[11]^ In contrast, the triple Ca−H−Ca‐bridged dimer cation **3^+^** [LCaH_3_CaL]^+^ formally resulting from a hydride attachment to **1^2+^** showed significantly lower catalytic activity.^[10]^ The terminal Ca−H bond of reactive cation monomer LCaH^+^ was proposed to catalyze both the 1‐alkene hydrogenation and H_2_ isotope exchange reactions,[^10^, ^11^] as supported by the observed partial reaction order of 0.5 in both the **1^2+^** ⋅ THF‐ and the **2^2+^**‐catalyzed hydrogenation of 1‐alkenes (CH_2_=CH_2_ and 1‐octene).[^11^, ^12^]

In view of the remarkable catalytic activity of double Ca−H−Ca‐bridged cation dimers **1^2+^** ⋅ THF and **2^2+^** in the hydrogenation of unactivated 1‐alkenes with H_2_, it is highly desirable to make clear the origin of the observed ligand ring‐size effect on the catalytic activity.[^10^, ^11^] Stable Ca−H−Ca bridges may react via cooperative FLP‐like reactivity known for similar M−H−M bridges (metal M=Li, K, Al),^[13]^ although terminal Ca−H bonds of calcium hydride monomers are expected to be intrinsically more reactive especially when stabilized by very bulky ligands.[^7^, ^10^, ^11^] Very recently, our extensive dispersion‐corrected DFT calculations have shown that both monomeric and dimeric mechanisms can be involved depending on the nature of substrates (unactivated vinyl‐cyclohexene, conjugation‐activated styrene, and H_2_) even with the same **1^2+^** ⋅ THF catalyst.^[14]^

In this DFT‐mechanistic work, using **1^2+^** ⋅ THF and **2^2+^** as catalysts and CH_2_=CHBu, CH_2_=CH_2_ and H_2_ as substrates of different size, the origin of the experimentally observed ligand ring‐size effect is explored. This effect is actually due to a subtle compromise of solvent, steric and dinuclear cooperative effects via competitive Ca−H−Ca bridges and terminal Ca−H bonds (Scheme 1B). Even though the steric hindrance within the Ca−H−Ca‐bridged cation dimers is increasing in the order of **1^2+^** <**1^2+^** ⋅ THF<**2^2+^**, the reactivity of terminal Ca−H bonds of the transient cation monomers is also increasing in the order of **1 m^+^** <**1 m^+^** ⋅ THF<**2 m^+^**, leading to a higher activity of **2^2+^** in the cation‐monomer‐catalyzed hydrogenation but to a lower activity for the cation‐dimer‐catalyzed H_2_ isotope exchange in contrast to that observed for **1^2+^** ⋅ THF.

## Results and Discussion

To gain mechanistic insight into the hydrogenation of unactivated 1‐alkenes and H_2_ isotope exchange reactions catalyzed by cation dimers **1^2+^** ⋅ THF and **2^2+^**, dispersion‐corrected DFT calculations are performed at the PW6B95‐D3/def2‐QZVP+COSMO‐RS//TPSS‐D3/def2‐TZVP+COSMO level in THF solution (see below for computational details), and final free energies (at 298 K and 1 m concentration) are used in our discussion unless noted otherwise.

**Figure 1:**
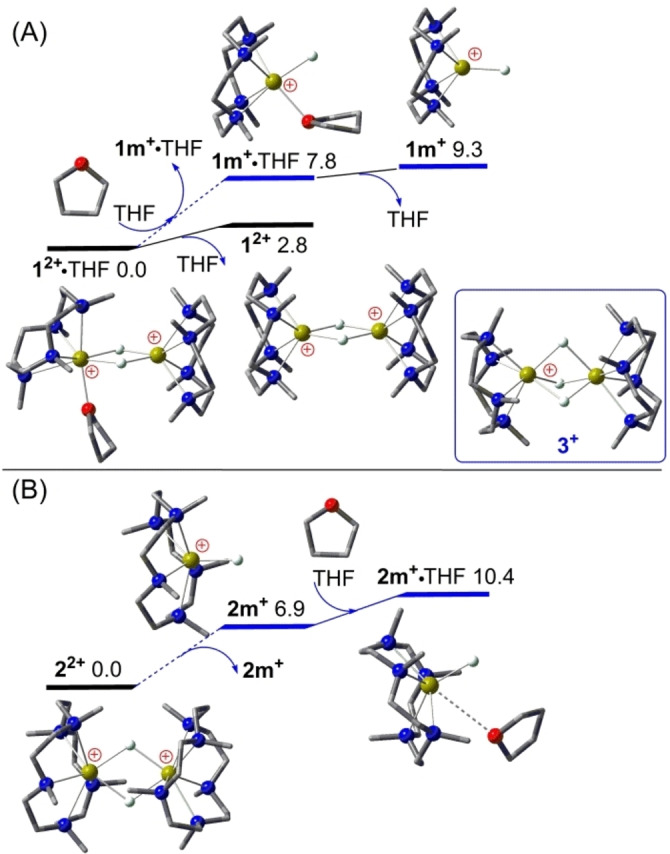
DFT‐computed free energy profile (in kcal mol^−1^, at 298 K and 1 m) for the dimer‐to‐monomer equilibrium in THF solution of (A) the L4‐stabilized cation dimer **1^2+^** ⋅ THF; (B) the L5‐stabilized cation dimer **2^2+^**. All molecules shown in ball‐and‐stick model, crucial Ca, N, O and H atoms are highlighted as yellow‐green, blue, red and white balls, while most H atoms are omitted for clarity.

As shown in Figure 1(A), our DFT calculations show that the L4‐stabilized **1^2+^** ⋅ THF and **1 m^+^** ⋅ THF complexes require 2.8 and 1.5 kcal mol^−1^ free energy to eliminate the coordinating THF, with decisive dispersion contributions of 7.6 and 4.2 kcal mol^−1^ favoring THF coordination, respectively. The stable complex **1^2+^** ⋅ THF was confirmed by an X‐ray structure at room temperature.^[9]^ Further THF coordination to **1^2+^** ⋅ THF smoothly leads to two **1 m^+^** ⋅ THF monomers which are 15.6 kcal mol^−1^ higher in free energy, indicating a rapid but endergonic dimer‐to‐monomer conversion in THF solution. When stabilized by larger L5 ligands instead, neither the crowded dimer **2^2+^** nor the small monomer **2 m^+^** is able to bind an additional THF ligand. The THF‐coordinated monomer **2 m^+^** ⋅ THF is already 3.5 kcal mol^−1^ less stable than free **2 m^+^** at room temperature, indicating that the L5 ligand may inhibit further THF coordination to the calcium cation. Moreover, the dimers **1^2+^** and **2^2+^** need 15.6 and 13.8 kcal mol^−1^ free energy to cleave the double Ca−H−Ca bridges stabilized by sizable dispersion contributions of 6.2 and 11.4 kcal mol^−1^, respectively, indicating the crucial role of dispersion interactions in the chemistry of calcium hydrides. A rapid dimer‐to‐monomer equilibrium is thus possible in THF solution for stable dimers of **1^2+^** ⋅ THF and **2^2+^** at room temperature, with the cleavage of **2^2+^** being indeed kinetically 1.8 kcal mol^−1^ easier than for **1^2+^** ⋅ THF. This is consistent with the increasing steric hindrance within Ca−H−Ca‐bridged dimers in the order of **1^2+^** <**1^2+^** ⋅ THF<**2^2+^**.

In contrast, the triple Ca−H−Ca‐bridged dimer cation **3^+^** can be formally obtained from a hydride attachment to the THF‐free cation dimer **1^2+^**. Further THF coordination to **3^+^** is energetically unfavorable while the direct cleavage of **3^+^** into the cation monomer **1 m^+^** and neutral monomer LCaH_2_ is 23.2 kcal mol^−1^ endergonic (see Supporting Information), suggesting a higher thermal stability than for the dimer **1^2+^** ⋅ THF. In such case, the involvement of a terminal Ca−H bond of cation monomer **1 m^+^** ⋅ THF from **3^+^** for further catalytic reactions is highly unlikely; instead, Ca−H−Ca bridges of **3^+^** should be directly involved in catalytic 1‐alkene hydrogenation and H_2_ isotope exchange reactions.

For comparison, dispersion‐uncorrected DFT calculations were used in previous mechanistic studies on related 1‐alkene hydrogenation reactions using the neutral calcium hydride dimer [(BDI)CaH_2_Ca(BDI)]^[8]^ or its THF‐coordinated counterpart [(THF)(BDI)CaH]_2_ ^[15]^ as catalysts. Dispersion‐uncorrected DFT calculations in the gas phase suggested that the dimer [(BDI)CaH_2_Ca(BDI)] is 40.4 kcal mol^−1^ lower in enthalpy than two (BDI)CaH monomers,^[8]^ but it should be further enhanced by 17.5 kcal mol^−1^ due to dispersion interactions and decreased by 26.8 kcal mol^−1^ due to benzene solvation, respectively, according to our recent dispersion‐corrected DFT calculations.^[14]^ In benzene solution, the dimer [(THF)(BDI)CaH]_2_ was computed to be about 7.7 kcal mol^−1^ higher in free energy than two (THF)(BDI)CaH monomers,^[15]^ in sharp contrast to previous experimental^[1c]^ and our recent dispersion‐corrected DFT results (−18.2 kcal mol^−1^ lower dimer than two monomers).^[14]^ It is thus crucial to include both dispersion corrections and suitable solvation in modeling such catalytic reactions in solution.

Natural population analysis^[16]^ is performed at the TPSS−D3/def2‐TZVP+COSMO level to understand the bonding situation at central calcium and hydride atoms. The calcium atoms of the complexes **1^2+^**, **2^2+^**, **3^+^**, **1 m^+^**, **2 m^+^**, and **1 m^+^** ⋅ THF adopt valence electron configurations of 4 s^0.21^3d^0.11^, 4 s^0.20^3d^0.11^, 4 s^0.23^3d^0.12^, 4 s^0.25^3d^0.13^, 4 s^0.23^3d^0.11^ and 4 s^0.20^3d^0.11^, respectively, leading to a positive charge of about 1.7 electrons with a small 3*d*‐orbital contribution of about 0.1 electron on each calcium center, consistent with recent bonding analysis on related calcium compounds.^[2]^ With a negative charge of about −0.8 electron on each hydride, the Ca−H bonds show strongly ionic bonding nature with about 30 % dative bond nature. The THF coordination to cation monomer **1 m^+^** may enhance the negative charge on the hydride from −0.77 to −0.85 electron and reduce the Wiberg bond index of the terminal Ca−H bond from 0.93 to 0.71, suggesting a higher reactivity of **1 m^+^** ⋅ THF.

**Figure 2:**
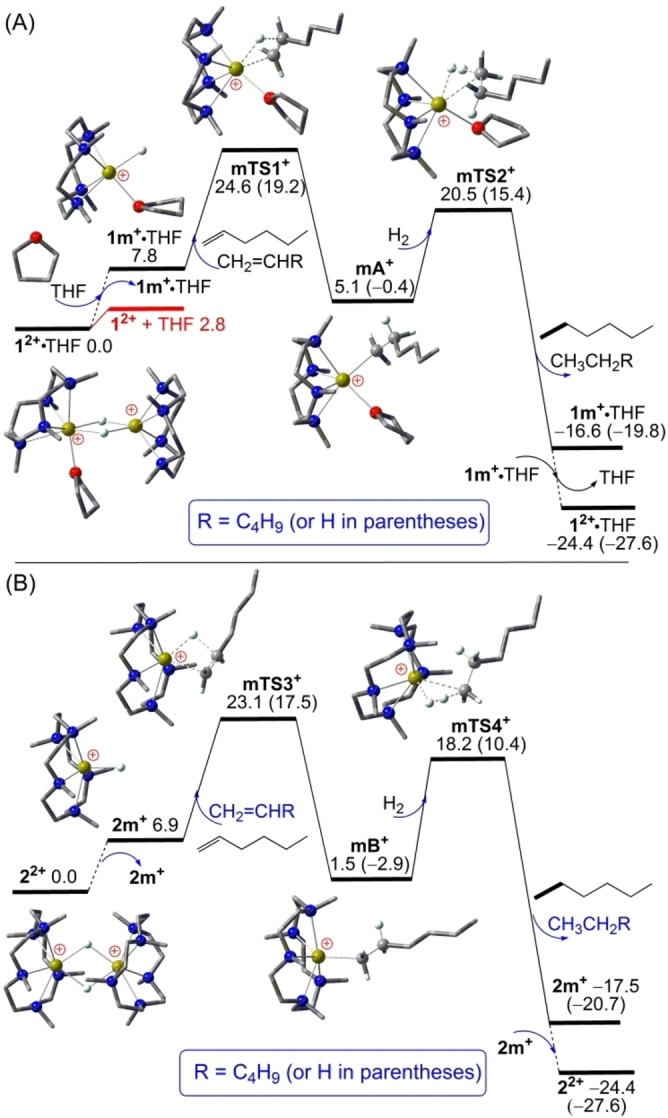
DFT‐computed free energy profile (in kcal mol^−1^, at 298 K and 1 m) for the hydrogenation of unactivated CH_2_=CHR (R=butyl or H) catalyzed by: (A) the L4‐stabilized cation dimer **1^2+^** ⋅ THF; (B) the L5‐stabilized cation dimer **2^2+^**. In both cases of catalysts, *anti‐*Markovnikov 1‐alkene addition to the terminal Ca−H bond of the respective cation monomer is the rate‐limiting step. In the ball‐and‐stick models, crucial Ca, N, O and H atoms are highlighted as yellow‐green, blue, red and white balls, while most H atoms are omitted for clarity.

As shown in Figure 2(A), starting from **1^2+^** ⋅ THF, the *anti*‐Markovnikov‐selective addition of unactivated 1‐hexene CH_2_=CHBu (Bu=butyl) to the terminal Ca−H bond of **1 m^+^** ⋅ THF is 5.1 kcal mol^−1^ endergonic over a sizeable barrier of 24.6 kcal mol^−1^ (via transition state **mTS1^+^**) to form the THF‐coordinated calcium alkyl complex **mA^+^** LCaCH_2_CH_2_Bu^+^ ⋅ THF. Further hydrogenolysis of the Ca−C bond of **mA^+^** is kinetically 4.1 kcal mol^−1^ more favorable (via **mTS2^+^**) than the preceding alkene addition step and is −21.7 kcal mol^−1^ exergonic to release the hexane product CH_3_CH_2_Bu along with **1 m^+^** ⋅ THF, followed by exergonic dimerization of two **1 m^+^** ⋅ THF complexes to regenerate the catalyst **1^2+^** ⋅ THF. The catalytic hydrogenation of CH_2_=CHBu via this monomeric mechanism is −24.4 kcal mol^−1^ exergonic over a sizeable barrier of 24.6 kcal mol^−1^, consistent with the moderate heating at 60 °C required experimentally.^[10]^ The same monomeric mechanism was also found in our recent DFT calculations using the CH_2_=CHCye (Cye=cyclohexenyl) substrate.^[14]^ When smaller ethylene (CH_2_=CH_2_) is used as substrate instead, the overall catalytic hydrogenation becomes −27.6 kcal mol^−1^ over a 5.4 kcal mol^−1^ lower barrier of 19.2 kcal mol^−1^, suggesting a much faster reaction even at room temperature. Due to reduced steric hindrance, direct CH_2_=CH_2_ addition to a Ca−H−Ca bridge of cation dimer **1^2+^** becomes kinetically only 0.5 kcal mol^−1^ less favorable (via **eTS1^2+^**, see Supporting Information). For comparison, similar addition of CH_2_=CHBu and CH_2_=CH_2_ to a Ca−H−Ca bridge of dimer cation **3^+^** encounters relatively higher barriers of 26.3 and 21.8 kcal mol^−1^ (via **TS7^+^** and **eTS7^+^**, see Supporting Information), respectively, consistent with the lower catalytic activity of **3^+^** compared to **1^2+^** ⋅ THF as observed experimentally.^[10]^

As shown in Figure 2(B), starting from **2^2+^** stabilized by larger L5 ligands, the *anti*‐Markovnikov‐selective addition of CH_2_=CHBu to the terminal Ca−H bond of *THF‐free* **2 m^+^** is still 1.5 kcal mol^−1^ endergonic over a sizeable barrier of 23.1 kcal mol^−1^ (via **mTS3^+^**) to form the calcium alkyl complex **mB^+^**. Further hydrogenolysis of the Ca−C bond of **mB^+^** is kinetically 4.9 kcal mol^−1^ more favorable (via **mTS4^+^**) than the preceding alkene addition step and is −19.0 kcal mol^−1^ exergonic to release the hexane product CH_3_CH_2_Bu along with **2 m^+^**, followed by exergonic **2 m^+^** dimerization to regenerate the catalyst **2^2+^**. Compared with the above **1^2+^** ⋅ THF‐catalyzed hydrogenation of CH_2_=CHBu, the **2^2+^**‐catalyzed one is indeed kinetically 1.5 kcal mol^−1^ more favorable and insensitive to THF coordination, consistent with the higher catalytic activity observed for the **2^2+^** catalyst stabilized by larger L5 ligands.^[11]^ When smaller CH_2_=CH_2_ is used as substrate instead, the overall barrier for catalytic hydrogenation is reduced to only 17.5 kcal mol^−1^ via the cation monomer **2 m^+^**, consistent with the rapid reaction observed even at 0 °C.^[11]^ Due to stronger steric hindrance, direct CH_2_=CH_2_ addition to a Ca−H−Ca bridge of L5‐stabilized **2^2+^** encounters a high free energy barrier of 36.6 kcal mol^−1^ and thus is kinetically highly disfavored. Moreover, compared with **1^2+^** ⋅ THF‐catalyzed hydrogenation of CH_2_=CHBu, the 1.5 kcal mol^−1^ lower barrier computed for the catalyst **2^2+^** is due to not only the 0.9 kcal mol^−1^ lower free energy for the monomer **2 m^+^** formation, but also to the 0.6 kcal mol^−1^ lower alkene addition barrier to *THF‐free* **2 m^+^** than to THF‐coordinated **1^+^** ⋅ THF. In other words, the observed “ligand ring‐size” effect^[11]^ is actually due to a subtle balance of steric and solvent coordination effects.

**Figure 3:**
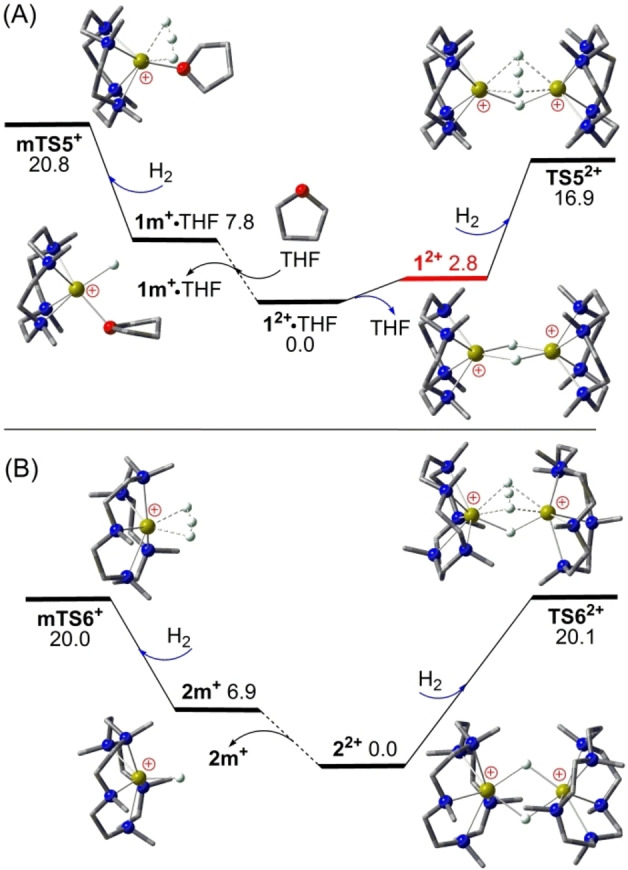
Comparison of DFT‐computed free energy profiles (in kcal mol^−1^, at 298 K and 1 m) for H_2_ isotope exchange catalyzed by (A) the L4‐stabilized cation dimer **1^2+^** ⋅ THF used in our recent work;^[14]^ (B) the larger L5‐stabilized cation dimer **2^2+^**. In the ball‐and‐stick models, crucial Ca, N, O and H atoms are highlighted as yellow‐green, blue, red and white balls, while most H atoms are omitted for clarity.

When very small dihydrogen is used as substrate for catalytic H_2_ isotope exchange, the dimeric mechanism becomes more important due to evidently reduced steric hindrance and stronger dinuclear cooperative effects. As seen in Figure 3(A), according to our recent DFT calculations, a very facile H⋅⋅⋅H⋅⋅⋅H‐type H_2_ isotope exchange may occur via a cooperative Ca−H−Ca bridge of THF‐free cation dimer **1^2+^** over a low barrier of 16.9 kcal mol^−1^ (via **TS5^2+^**) after THF elimination,^[14]^ consistent with the very fast reaction observed at room temperature.^[10]^ The three exchanging hydrogen atoms are placed evenly between two calcium ions and perpendicular to the other Ca−H−Ca bridge, suggesting potentially strong dinuclear cooperative effects. Such H_2_ isotope exchange via a Ca−H−Ca bridge of THF‐coordinated **1^2+^** ⋅ THF becomes kinetically 5.0 kcal mol^−1^ less favorable (via **TS5a^2+^**), mainly due increased steric hindrance. On the other hand, the cation monomer **1 m^+^** ⋅ THF is 7.8 kcal mol^−1^ higher in free energy but intrinsically 8.9 kcal mol^−1^ more reactive than **1^2+^** ⋅ THF, and thus is kinetically 1.1 kcal mol^−1^ more favorable for catalytic H_2_ isotope exchange (via **mTS5^+^**). However, **1^2+^** ⋅ THF remains kinetically 3.9 kcal mol^−1^ less active than **1^2+^** for the catalytic H_2_ isotope exchange. Considering the overall reaction of **1^2+^** ⋅ THF+H_2_→**TS5^2+^** +THF, it is clear that such H_2_ isotope exchange is disfavored by the THF coordination by about 2.8 kcal mol^−1^. For comparison, a similar dimeric mechanism for cation **3^+^**‐catalyzed H_2_ isotope exchange (via **TS9^+^**, see Supporting Information) encounters a moderate but higher free energy barrier of 21.7 kcal mol^−1^, thus is kinetically less efficient than **1^2+^**.

As seen in Figure 3(B), starting from the L5‐stabilized cation dimer **2^2+^**, a similar cooperative Ca−H−Ca‐bridge‐mediated H_2_ isotope exchange may also occur but over a 3.2 kcal mol^−1^ higher barrier of 20.1 kcal mol^−1^ (via **TS6^2+^**). Interestingly, the terminal Ca−H mediated H_2_ isotope exchange via the *THF‐free* cation monomer **2 m^+^** becomes kinetically competitive over nearly the same barrier of 20.0 kcal mol^−1^ (via **mTS6^+^**). In contrast to the **1^2+^**‐catalyzed H_2_ isotope exchange that is disfavored by THF coordination within stable **1^2+^** ⋅ THF, those reactions catalyzed by both the cation dimer **2^2+^** and the cation monomer **2 m^+^** stabilized by the larger L5 ligand are not disfavored by the THF solvent but remain kinetically 3.2 kcal mol^−1^ less favorable, in sharp contrast to the cases of catalytic hydrogenation of unactivated 1‐alkenes. In other words, no simple “ligand ring‐size” effects can be expected for calcium hydride catalysts, with the catalytic activity actually being due to a subtle balance between solvent, steric and dinuclear cooperative effects.

## Conclusion

By using cationic dimers **1^2+^** ⋅ THF and **2^2+^** as catalysts stabilized by macrocyclic L4 and larger L5 ligands and using CH_2_=CHBu, CH_2_=CH_2_ and H_2_ as substrates of different size, the origin of the experimentally observed ligand ring‐size effect is explored by extensive dispersion‐corrected DFT calculations. It is disclosed that the catalytic activity of calcium hydride cation dimer catalysts can be influenced by a subtle interplay of solvent, steric and substrate‐dependent dinuclear cooperative effects that may change the underlying mechanism in 1‐alkene hydrogenation and H_2_ isotope exchange reactions.

## Computational Methods

All DFT calculations are performed with the TURBOMOLE 7.4 suite of programs.^[17]^ The structures are fully optimized at the TPSS−D3/def2‐TZVP+COSMO level in THF solution, which combines the TPSS meta‐GGA density functional^[18]^ with the BJ‐damped DFT−D3 dispersion correction^[19]^ and the def2‐TZVP basis set,^[20]^ using the Conductor‐like Screening Model (COSMO)^[21]^ for THF solvent (dielectric constant ϵ=7.58 and radius R_solv_=3.18 Å). The density‐fitting RI−J approach^[22]^ is used to accelerate the calculations. The optimized structures are characterized by frequency analysis (no imaginary frequency for true minima and only one imaginary frequency for transition states) to provide thermal free‐energy corrections (at 298.15 K and 1 atm) according to the modified ideal gas‐rigid rotor‐harmonic oscillator model.^[23]^

More accurate solvation free energies in THF solution are computed with the COSMO‐RS model^[24]^ (parameter file: BP_TZVP_C30_1601.ctd) using the COSMOtherm package^[25]^ based on the TPSS−D3 optimized structures, corrected by +1.89 kcal mol^−1^ to account for the 1 mol L^−1^ reference concentration in solution. To check the effects of the chosen DFT functional on the reaction energies and barriers, single‐point calculations at both TPSS‐D3^[18]^ and hybrid‐meta‐GGA PW6B95‐D3^[26]^ levels are performed using the larger def2‐QZVP^[20]^ basis set. Final reaction free energies (ΔG) are determined from the electronic single‐point energies plus TPSS‐D3 thermal corrections and COSMO‐RS solvation free energies. As also noted previously for similar hydrogenation reactions,[^13^, ^27^] the reaction energies from both DFT functionals are in very good mutual agreement of −0.2±1.4 kcal mol^−1^ (mean±standard deviation) though as expected 0.5±1.5 kcal mol^−1^ higher barriers are found at the PW6B95‐D3 level. In our discussion, the more reliable PW6B95‐D3+COSMO‐RS free energies (in kcal mol^−1^, at 298.15 K and 1 mol L^−1^ concentration) are used unless specified otherwise. The applied DFT methods in combination with the large AO basis set provide usually accurate electronic energies leading to errors for chemical energies (including barriers) on the order of typically 1–2 kcal mol^−1^. This has been tested thoroughly for the huge data base GMTKN55^[28]^ which is the common standard in the field of DFT benchmarking. See Ref. [29] for general recommendations on DFT‐based computational chemistry studies.

## Conflict of interest

The authors declare no conflict of interest.

1

## Supporting information

As a service to our authors and readers, this journal provides supporting information supplied by the authors. Such materials are peer reviewed and may be re‐organized for online delivery, but are not copy‐edited or typeset. Technical support issues arising from supporting information (other than missing files) should be addressed to the authors.

Supporting Information

## Data Availability

The data that support the findings of this study are available in the supplementary material of this article.
