# Supplementary material for: Origin of the Ligand Ring‐Size Effect on the Catalytic Activity of Cationic Calcium Hydride Dimers in the Hydrogenation of Unactivated 1‐Alkenes
Source: ChemistryOpen. 2022 Dec 16;11(12):e202200240. doi: 10.1002/open.202200240 (PMC9756592; doi:10.1002/open.202200240)
Supplement: Supplementary file 1 — Supporting Information [file OPEN-11-e202200240-s001.pdf]

ChemistryOpen

Supporting Information

Origin of the Ligand Ring-Size Effect on the Catalytic Activity of Cationic Calcium Hydride Dimers in the Hydrogenation of Unactivated 1-Alkenes

Hui Zhu, Zheng-Wang Qu,* and Stefan Grimme

Table of Contents

Table S1. DFT-computed energies in THF solution. S2

Table S2. DFT-optimized Cartesian coordinates in THF solution S6

Table S1. DFT-computed energies in toluene solution. TPSS-D3/def2-TZVP + COSMO computed imaginary frequency (ImF), zero-point energies (ZPE), enthalpic (Hc) and Gibbs free-energy (Gc) corrections; the COSMO-RS computed solvation enthalpic (Hsol) and Gibbs free-energy (Gsol) corrections in THF solution; TPSS-D3/def2-QZVP and PW6B95-D3/def2-QZVP single-point energies (TPSS-D3 and PW6B95-D3 (E_P)); total PW6B95-D3 Gibbs free energies ($G_P = E_P + Gc + Gsol$) at 298 K, relative electronic energies (ΔE_T and ΔE_P) and final Gibbs free-energies (ΔG_T and ΔG_P) at the TPSS-D3 and PW6B95-D3 levels at 298 K. Each structure is labeled either by its molecular formula or a specific name in bold, with singly and doubly charged cations indicated by the + and 2+ superscripts, respectively. Dimeric and monomeric transition structures (with only one imaginary frequency) are indicated by the "**TS**" and "**mTS**" prefixes, respectively. See also main-text **Figures 1** and **2** for structural labelling. The final PW6B95-D3 Gibbs free energies at 298 K and 1 M concentration are used in our discussion.

Reactions in THF (1M)	ImF cm^{-1}	ZPE kcal/mol	Hc kcal/mol	Gc kcal/mol	Hsol kcal/mol	Gsol kcal/mol	TPSS-D3 E_h	PW6B95-D3 E_h	G_P E_h	ΔE_T kcal/mol	ΔE_P kcal/mol	*ΔG_P kcal/mol*	ΔG_T kcal/mol
THF	0	71.89	75.64	54.80	-6.76	-4.40	-232.60386	-232.84221	-232.75888	0.00	0.00	*0.00*	0.00
With L4 ligand													
*Only one THF molecule is bound to the cation monomer **1m**$^+$.*													
**1m**$^+$ + 2THF	0	400.19	421.41	337.47	-80.00	-64.36	-1836.91120	-1838.53617	-1838.09191	0.00	0.00	*0.00*	0.00
**1m**$^+$•THF + THF	0	401.81	422.88	352.09	-67.84	-56.08	-1836.94546	-1838.57198	-1838.09423	-21.49	-22.47	*-1.46*	-0.48
**1m**$^+$•$(THF)_2$	0	403.37	424.60	366.74	-58.21	-49.81	-1836.97125	-1838.60153	-1838.09345	-37.68	-41.02	*-0.96*	2.37
*One THF molecule is -2.80 kcal/mol bound to the cation dimer **1**$^{2+}$*													
**1**$^{2+}$•THF – THF	0	517.73	544.53	490.34	-131.38	-121.99	-2743.46265	-2745.76467	-2745.17767	0.00	0.00	*0.00*	0.00
**1**$^{2+}$	0	516.49	542.91	476.67	-141.26	-127.96	-2743.43373	-2745.73194	-2745.17323	18.15	20.54	*2.79*	0.40
*..while the second THF coordination leads to dimer cleavage into **1m**$^+$•THF*													
**1**$^{2+}$•$(THF)_2$ - 2THF	0	519.47	546.37	505.57	-124.17	-117.03	-2743.46666	-2745.77044	-2745.15427	-2.52	-3.62	*14.68*	15.79
2***1m**$^+$•THF- 2THF	0	516.05	543.19	485.01	-108.66	-94.58	-2743.47549	-2745.77511	-2745.15292	-8.06	-6.55	*15.53*	14.02
2***1m**$^+$	0	512.81	540.25	455.75	-132.98	-111.14	-2743.40699	-2745.70348	-2745.14828	34.93	38.40	*18.44*	14.97
With unactivated 1-hexene (CH_2=CHBu) substrate													
*Anti-Markovnikov alkene addition to terminal Ca-H bond of cation monomer over a sizeable barrier (via **mTS1**$^+$) of 24.6 kcal/mol*													
**1m**$^+$•THF + CH_2=CHBu	0	431.88	454.84	379.38	-68.08	-55.66	-1840.34748	-1841.98469	-1841.46279	0.00	0.00	*7.77*	0.00
**mTS1**$^+$	352i	432.91	455.45	395.72	-57.43	-48.85	-1840.35473	-1841.99181	-1841.43603	-4.55	-4.47	*24.56*	16.71
**mA**$^+$	0	436.51	459.28	398.78	-60.26	-50.85	-1840.38406	-1842.02459	-1841.46711	-22.95	-25.04	*5.06*	-0.63
**1m**$^+$•THF + CH_2=CHBu + H_2	0	438.21	463.24	378.50	-68.95	-54.66	-1841.52807	-1843.16063	-1842.63552	0.00	0.00	*7.77*	0.00
**mTS2**$^+$	803i	445.36	468.36	407.03	-58.23	-49.31	-1841.55554	-1843.18825	-1842.61519	-17.24	-17.33	*20.53*	12.85

**1m**$^+$•THF + CH_3CH_3	0	446.35	469.66	393.50	-67.80	-55.36	-1841.58348	-1843.21923	-1842.67435	-34.77	-36.77	*-16.60*	-22.37
With small ethylene CH_2=CH_2 as substrate with less steric hindrance													
*Addition via cation monomer **1m**$^+$**.**THF is almost neutral in free energy over a moderate barrier (via **eTS1**$^+$) of 19.2 kcal/mol*													
**1m**$^+$•THF + CH_2=CH_2	0	361.47	381.29	315.75	-64.07	-52.83	-1682.98321	-1684.45362	-1684.02861	0.00	0.00	*7.77*	0.00
**eTS1**$^+$	345i	362.89	381.92	329.21	-56.49	-48.34	-1682.99142	-1684.46101	-1684.01041	-5.15	-4.64	*19.19*	10.91
**eA**$^+$	0	365.72	385.28	331.27	-57.63	-49.25	-1683.02229	-1684.49402	-1684.04158	-24.52	-25.35	*-0.37*	-7.30
**eA0**$^+$ + THF	0	364.17	383.79	315.96	-68.52	-56.53	-1682.98978	-1684.45755	-1684.03811	-4.12	-2.47	*1.81*	-7.61
..followed by very facile Ca–C bond hydrogenolysis with H_2 to release CH_3CH_3													
**1m**$^+$•THF + CH_2=CH_2 + H_2	0	367.80	389.70	314.88	-64.94	-51.83	-1684.16381	-1685.62956	-1685.20134	0.00	0.00	*7.77*	0.00
**eTS2**$^+$	822i	375.01	394.63	340.67	-55.59	-47.59	-1684.19299	-1685.65917	-1685.18910	-18.31	-18.58	*15.45*	7.95
CH_3CH_3 + **1m**$^+$•THF	0	376.00	396.12	329.92	-63.72	-52.54	-1684.22466	-1685.69333	-1685.24527	-38.19	-40.01	*-19.80*	-25.74
*Addition via cation dimer **1**$^{2+}$ is -5.4 kcal/mol exergonic but kinetically 0.5 kcal/mol less favorable.*													
**1**$^{2+}$ + CH_2=CH_2	0	548.04	576.96	495.12	-144.25	-129.11	-2822.07534	-2824.45579	-2823.86649	0.00	0.00	*2.79*	0.00
**eTS1**$^{2+}$	642i	549.93	577.99	508.57	-141.57	-127.99	-2822.06591	-2824.44909	-2823.83958	5.92	4.20	*19.68*	18.60
**eA**$^{2+}$	0	552.25	580.82	510.21	-140.74	-127.72	-2822.10666	-2824.49217	-2823.87961	-19.65	-22.82	*-5.44*	-5.06
..followed by very facile H2 hydrogenolysis with H_2 to release CH_3CH_3													
**1**$^{2+}$ + CH_2=CH_2 + H_2	0	554.37	585.37	494.24	-145.12	-128.11	-2823.25593	-2825.63173	-2825.03922	0.00	0.00	*2.79*	0.00
**eTS2**$^{2+}$	742i	561.47	589.69	519.94	-139.48	-126.87	-2823.27355	-2825.65265	-2825.02323	-11.06	-13.12	*12.83*	12.10
CH_3CH_3 + **1**$^{2+}$	0	562.57	591.79	509.29	-143.90	-128.82	-2823.31679	-2825.69550	-2825.08315	-38.19	-40.01	*-24.78*	-25.74
..the second ethylene CH_2=CH_2 addition is prevented by a sizable barrier of 27.7 kcal/mol													
**eA**$^{2+}$ + CH_2=CH_2	0	583.80	614.87	528.66	-143.72	-128.86	-2900.74827	-2903.21602	-2902.57288	0.00	0.00	*0.00*	0.00
**eTS1a**$^{2+}$	639i	585.58	615.76	542.55	-143.29	-128.90	-2900.72463	-2903.19097	-2902.52876	14.84	15.72	*27.68*	26.80
With larger L5 ligand													
*The dissociation of cation dimer **2**$^{2+}$ needs 1.8 kcal/mol less free enery thus is less stable than **1**$^{2+}$.THF*													
**2**$^{2+}$	0	643.22	675.57	598.20	-136.60	-125.69	-3090.24100	-3092.90758	-3092.15157	0.00	0.00	*0.00*	0.00
2***2m**$^+$	0	641.01	673.58	579.71	-123.46	-104.01	-3090.23491	-3092.89380	-3092.12971	3.82	8.64	*13.72*	8.89
*..while no THF is bound to the cation monomer **2m**$^+$ at room temperature*													
**2m**$^+$ + THF	0	392.40	412.43	344.65	-68.48	-56.40	-1777.72131	-1779.28911	-1778.82374	0.00	0.00	*0.00*	0.00
**2m**$^+$•THF	0	393.29	413.91	357.78	-59.34	-50.59	-1777.74257	-1779.31071	-1778.81816	-13.34	-13.55	*3.50*	3.71
*With unactivated 1-hexene (CH_2=CHBu) as substrate: alkene addition to terminal Ca-H bond encounters a barrier (via **mTS3**$^+$) of 23.1 kcal/mol*													

**2m**$^+$ + CH_2=CHBu	0	422.47	444.39	371.93	-68.72	-55.98	-1781.12333	-1782.70182	-1782.19230	0.00	0.00	*6.86*	0.00
**mTS3**$^+$	353i	423.03	444.64	386.95	-56.57	-48.45	-1781.13131	-1782.70893	-1782.16648	-5.00	-4.46	*23.06*	15.66
**mB**$^+$	0	425.81	447.95	388.52	-62.05	-52.56	-1781.16060	-1782.73927	-1782.20088	-23.39	-23.50	*1.47*	-5.28
..followed by faster hydrogenaolysis of Ca-C bond													
**2m**$^+$ + CH_2=CHBu + H_2	0	428.80	452.80	371.06	-69.59	-54.98	-1782.30392	-1783.87776	-1783.36503	0.00	0.00	*6.86*	0.00
**mTS4**$^+$	985i	435.17	457.28	398.45	-58.56	-49.68	-1782.33187	-1783.90573	-1783.34692	-17.53	-17.55	*18.23*	11.38
CH_3CH_2Bu + **2m**$^+$	0	436.94	459.21	386.05	-68.44	-55.68	-1782.35934	-1783.93636	-1783.40386	-34.77	-36.77	*-17.51*	-22.37
*With small ethylene CH_2=CH_2 as substrate: kinetically much more facile over a barrier of 17.5 kcal/mol via **eTS3**$^+$, exergonic intermediate **eB**$^+$ formation*													
**2m**$^+$ + CH_2=CH_2	0	352.06	370.85	308.30	-64.71	-53.15	-1623.75907	-1625.17075	-1624.75812	0.00	0.00	*6.86*	0.00
**eTS3**$^+$	373i	353.29	371.28	321.06	-55.44	-47.81	-1623.76884	-1625.17956	-1624.74110	-6.13	-5.53	*17.54*	10.08
**eB**$^+$	0	355.89	374.51	322.77	-58.54	-50.00	-1623.79959	-1625.21140	-1624.77370	-25.43	-25.51	*-2.91*	-9.70
..followed by kinetically faster hydrogenolysis of Ca-C bond													
**2m**$^+$ + CH_2=CH_2 + H_2	0	358.39	379.25	307.43	-65.58	-52.15	-1624.93966	-1626.34670	-1625.93086	0.00	0.00	*6.86*	0.00
**eTS4**$^+$	906i	365.04	383.64	332.18	-56.24	-48.35	-1624.97309	-1626.38054	-1625.92521	-20.98	-21.24	*10.40*	3.80
CH_3CH_3 + **2m**$^+$	0	366.59	385.68	322.48	-64.36	-52.86	-1625.00052	-1626.41046	-1625.97479	-38.19	-40.01	*-20.71*	-25.74
Proton transfer from THF is kinetically less favorable over a barrier 25.5 kcal/mol													
**eB**$^+$ + THF	0	427.78	450.15	377.57	-65.29	-54.40	-1856.40345	-1858.05361	-1857.53259	0.00	0.00	*-2.91*	0.00
**eB**$^+$•THF	0	429.42	451.97	392.09	-56.62	-48.82	-1856.42130	-1858.07495	-1857.52491	-11.20	-13.39	*1.91*	7.00
**eTS4a**$^+$	1447i	426.65	448.66	390.34	-55.09	-47.59	-1856.39122	-1858.04124	-1857.49202	7.67	7.77	*22.55*	25.36
**eC**$^+$ + CH_3CH_3	0	427.80	450.31	379.91	-57.61	-48.55	-1856.43044	-1858.08153	-1857.54745	-16.94	-17.52	*-12.24*	-8.75
*The calcium hydride cation dimer **2**$^{2+}$ is too crowded for direct 1-alkene addition*													
**2**$^{2+}$ + CH_2=CHBu	0	745.18	783.17	680.28	-143.59	-129.67	-3326.24688	-3329.16250	-3328.27901	0.00	0.00	*0.00*	0.00
**TS3**$^{2+}$	574i	746.09	783.63	696.09	-138.04	-125.71	-3326.21521	-3329.12774	-3328.21577	19.87	21.81	*39.69*	37.74
**2**$^{2+}$ + CH_2=CH_2	0	674.77	709.62	616.65	-139.58	-126.84	-3168.88261	-3171.63143	-3170.84484	0.00	0.00	*0.00*	0.00
**eTS3**$^{2+}$	649i	676.49	710.70	629.91	-139.55	-127.44	-3168.84829	-3171.59033	-3170.78658	21.54	25.79	*36.56*	32.30
H_2 isotope exchange reactions													
*With small L4 ligand: Very facile H_2 isotope exchange via the dimer **1**$^{2+}$ is 3.9 kcal/mol more reactive than via monomer **1m**$^+$.THF: cooperative effects*													
**1**$^{2+}$ + H_2	0	522.82	551.31	475.79	-142.13	-126.96	-2744.61432	-2746.90788	-2746.34596	0.00	0.00	*2.79*	0.00
**TS5**$^{2+}$	913i	525.51	552.08	485.42	-139.51	-127.24	-2744.60251	-2746.89729	-2746.32348	7.41	6.65	*16.90*	14.87
*..H_2 exchange via cation monomer **1m**$^+$.THF encounters a barrier (via **mTS5**$^+$) of 20.8 kcal/mol*													

**1m**$^+$•THF + H_2	0	336.25	355.64	296.43	-61.96	-50.69	-1605.52219	-1606.90571	-1606.50808	0.00	0.00	*7.77*	0.00
**mTS5**$^+$	1158i	338.40	356.10	305.91	-56.54	-48.27	-1605.51888	-1606.90090	-1606.48731	2.08	3.02	*20.80*	12.09
*..direct H_2 exchange with THF-coordinated cation dimer **1**$^{2+}$**.**THF is kinetically 4.4 kcal/mol less favorable.*													
**1**$^{2+}$•THF + H_2	0	595.95	628.58	544.26	-139.00	-125.39	-2977.24710	-2979.78283	-2979.10929	0.00	0.00	*0.00*	0.00
**TS5a**$^{2+}$	814i	598.26	628.94	554.23	-138.05	-126.00	-2977.22492	-2979.75988	-2979.07444	13.91	14.40	*21.86*	21.38
*..H_2 exchange via THF-free cation monomer **1m**$^+$ is kinetically disfavored: THF-coordination may enhance the hydride nature*													
**1m**$^+$ + H_2	0	262.74	278.53	227.00	-67.36	-54.57	-1372.88409	-1374.02768	-1373.74687	0.00	0.00	*9.22*	0.00
**1m**$^+$•H_2	0	265.25	279.94	236.29	-65.25	-54.57	-1372.88741	-1374.03123	-1373.73863	-2.09	-2.23	*14.39*	5.32
**mTS5a**$^+$	1346i	264.39	278.49	235.65	-61.45	-51.64	-1372.87810	-1374.01854	-1373.72229	3.76	5.74	*24.65*	13.44
With larger L5 ligand:													
*H_2 isotope exchange may be catalyzed by both cation dimer **2**$^{2+}$ and monomer **2m**$^+$ over similar barriers of 20.0 kcal/mol*													
**2**$^{2+}$ + H_2	0	649.55	683.98	597.33	-137.47	-124.69	-3091.42159	-3094.08352	-3093.32430	0.00	0.00	*0.00*	0.00
**TS6**$^{2+}$	792i	652.11	684.58	607.25	-135.93	-125.06	-3091.40457	-3094.06374	-3093.29232	10.68	12.41	*20.07*	18.33
**2m**$^+$ + H_2	0	326.84	345.20	288.98	-62.60	-51.01	-1546.29805	-1547.62284	-1547.23759	0.00	0.00	*6.86*	0.00
**mTS6**$^+$	1194i	328.45	345.16	297.39	-56.45	-48.43	-1546.29303	-1547.61645	-1547.21669	3.15	4.01	*19.97*	12.25
*With small L4 ligand and an additional hydride: triple Ca-H-Ca bridged dimer cation **3**$^+$*													
*1.7 kcal/mol higher addition barrier is found for unactivated 1-alkene CH_2=CHBu: less active than the cation dimer **1**$^{2+}$.THF*													
**3**$^+$ + CH_2=CHBu	0	619.97	652.45	559.35	-61.09	-50.07	-2980.24962	-2982.80089	-2981.98328	0.00	0.00	*0.00*	0.00
**TS7**$^+$	457i	620.78	652.90	575.49	-58.82	-49.49	-2980.23165	-2982.78256	-2981.94131	11.27	11.50	*26.34*	26.11
**C**$^+$	0	624.08	656.35	578.42	-58.15	-48.77	-2980.27970	-2982.83443	-2981.98737	-18.87	-21.05	*-2.57*	-0.39
..followed by kinetically faster hydrogenolysis with H_2 of the Ca-C bond													
**3**$^+$ + CH_2=CHBu + H_2	0	626.30	660.86	558.47	-61.96	-49.07	-2981.43021	-2983.97683	-2983.15601	0.00	0.00	*0.00*	0.00
**C**$^+$•H_2	0	631.21	665.63	583.48	-58.48	-48.99	-2981.46245	-2984.01281	-2983.15805	-20.23	-22.58	*-1.28*	1.08
**TS8**$^+$	1107i	632.68	665.32	586.72	-60.29	-50.42	-2981.43612	-2983.98543	-2983.12778	-3.71	-5.40	*17.71*	19.41
**3**$^+$ + CH_3CH_2Bu	0	634.44	667.27	573.46	-60.81	-49.77	-2981.48563	-2984.03543	-2983.19484	-34.77	-36.77	*-24.37*	-22.37
..A moderate barrier for catalytic H_2 isotope exchange													
**3**$^+$ + H_2	0	524.34	553.26	476.39	-54.97	-45.09	-2745.42434	-2747.72191	-2747.02857	0.00	0.00	*0.00*	0.00
**3**$^+$•H_2	0	525.16	553.98	482.19	-54.18	-46.07	-2745.42217	-2747.71981	-2747.02180	1.36	1.32	*4.25*	4.29
**TS9**$^+$	1062i	527.33	553.90	487.67	-55.67	-47.13	-2745.40187	-2747.69903	-2746.99397	14.10	14.36	*21.71*	21.45

Table S2. TPSS-D3/def2-TZVP + COSMO optimized Cartesian coordinates (in Å) in THF solution. Each structure is labeled by the specific name (See also **Table S1** and main-text **Figures 1** and **2**), followed by the number of atoms, the total energy, and the detailed atomic coordinates (in double-column text list).

3^{+}•CH_2=CHBu : loose complex of 1-hexene

111

Energy = -2980.175585332

Ca	1.6805451	-0.7359355	-0.1526429
H	0.0988126	-1.2314070	-1.6059253
N	2.8823875	-2.7379548	-1.4921432
N	3.1613805	0.2627795	-2.0294170
N	3.6019870	0.7868858	0.9096492
N	3.2459013	-2.1861578	1.4272403
C	3.9612419	-2.0966027	-2.2751927
H	4.3568488	-2.7985744	-3.0288363
H	4.7879411	-1.8634837	-1.5990445
C	3.4947412	-0.8264010	-2.9766743
H	4.2697652	-0.4994287	-3.6895671
H	2.5901289	-1.0339960	-3.5546294
C	4.3887017	0.8788399	-1.4779658
H	4.8378351	1.5710305	-2.2092702
H	5.1225484	0.0863317	-1.3097980
C	4.1375251	1.6393020	-0.1795562
H	5.0744325	2.1314694	0.1312755
H	3.4015479	2.4310926	-0.3512347
C	4.6439319	-0.1202783	1.4417828
H	5.3590976	0.4404423	2.0661841
H	5.2108592	-0.5258178	0.6001738
C	4.0492475	-1.2615909	2.2597000
H	4.8604635	-1.8015849	2.7750922
H	3.3901214	-0.8631913	3.0361058
C	4.1402468	-3.0702079	0.6457729
H	4.5594690	-3.8599166	1.2912762
H	4.9861649	-2.4752342	0.2928183
C	3.4364275	-3.7223959	-0.5361764
H	4.1403334	-4.4102578	-1.0352726
H	2.5985916	-4.3294236	-0.1809093
C	1.9576122	-3.4351550	-2.4076245
H	1.4408983	-2.7109452	-3.0375169
H	1.2031090	-3.9554891	-1.8165183
H	2.4964932	-4.1686650	-3.0316011
C	2.3396335	1.2710131	-2.7310729
H	2.1436285	2.1159670	-2.0697703
H	1.3805671	0.8164408	-2.9934342
H	2.8462513	1.6351541	-3.6397534
C	3.1159037	1.6599480	1.9976484
H	2.6520830	1.0599130	2.7833365
H	2.3655584	2.3433509	1.5990902
H	3.9406286	2.2429877	2.4387610
C	2.3672780	-2.9958885	2.2970098
H	1.7913949	-3.6965101	1.6888347
H	1.6569022	-2.3372338	2.8001384
H	2.9539686	-3.5653570	3.0369165
Ca	-1.4615498	-0.3407645	-0.2622418
H	-0.0084821	-1.2837375	1.1885414
N	-3.2180880	-0.5207317	1.6999398
N	-2.4208165	-2.7477299	-0.2013759
N	-2.7281341	-0.7741163	-2.4885449
N	-3.5692892	1.4145057	-0.6130543
C	-3.9747500	-1.7794010	1.5029282
H	-4.5205847	-2.0423833	2.4246524
H	-4.7286283	-1.6096494	0.7299061
C	-3.0837318	-2.9489789	1.1044304
H	-3.6853373	-3.8740621	1.1068612
H	-2.2870597	-3.0760191	1.8426383
C	-3.3577313	-2.9092457	-1.3333744
H	-3.5429100	-3.9775827	-1.5362299
H	-4.3205214	-2.4738886	-1.0523936
C	-2.8326214	-2.2472892	-2.6050157
H	-3.4768415	-2.5284242	-3.4554459
H	-1.8226927	-2.6100067	-2.8165051
C	-4.0580439	-0.1345588	-2.5256698
H	-4.4676541	-0.1409552	-3.5499376
H	-4.7422780	-0.7254205	-1.9112751
C	-4.0174035	1.3025729	-2.0225762
H	-5.0137615	1.7579549	-2.1503562
H	-3.3218332	1.8903259	-2.6284443
C	-4.6500962	0.9800591	0.3032857
H	-5.4210700	1.7643043	0.3840199
H	-5.1400808	0.1071672	-0.1342018
C	-4.1377075	0.6395554	1.6971950
H	-4.9981583	0.4647047	2.3646645
H	-3.5833278	1.4896523	2.1078094
C	-2.4931983	-0.5708331	2.9876891
H	-1.7269172	-1.3460552	2.9450278
H	-1.9867099	0.3834149	3.1462452
H	-3.1845960	-0.7596044	3.8258952
C	-1.3053582	-3.7039420	-0.3248189
H	-0.8097283	-3.5506421	-1.2829488
H	-0.5821811	-3.4843976	0.4645767
H	-1.6622553	-4.7456253	-0.2481671
C	-1.8810334	-0.2654957	-3.5875603
H	-1.7825858	0.8203635	-3.5116882
H	-0.8881702	-0.7081588	-3.4736840
H	-2.3134254	-0.5108150	-4.5719274
C	-3.2466955	2.8311697	-0.3502206
H	-2.9885806	2.9665280	0.7007477
H	-2.3871274	3.1266599	-0.9557875
H	-4.1011604	3.4852287	-0.5895669
C	0.2551126	1.5478185	-0.0610804
C	-0.3358563	1.9845308	1.2863568
H	-0.2600971	2.1019859	-0.8739264
H	1.2901924	1.9300003	-0.1168405
H	-1.3867161	1.6402427	1.3730039
H	0.1774153	1.4554891	2.1026019
C	-0.3484307	3.4892553	1.6109515

H	0.6888755	3.8524819	1.6537434
H	-0.8227479	4.0324868	0.7819591
C	-1.0632524	3.8256919	2.9240747
C	-1.0984719	5.3229795	3.2482482
H	-0.5794802	3.2849261	3.7507043
H	-2.0964561	3.4455415	2.8792973
C	-1.8346664	5.6356790	4.5552035
H	-0.0688635	5.7018387	3.3058668
H	-1.5790746	5.8585581	2.4179200
H	-1.8450580	6.7114257	4.7627384
H	-2.8751808	5.2918483	4.5091447
H	-1.3552178	5.1330376	5.4037664

3^{+}•H_2 : loose complex of H_2 and cation **3**$^{+}$

95

Energy = -2745.334018155

Ca	-1.5878686	-0.0575004	-0.0786434
H	0.0123691	-0.8542486	-1.4208544
N	-3.3396020	-1.0235243	-1.8082201
N	-3.2250252	-1.7332164	1.1125525
N	-2.8420468	1.1785729	1.7922664
N	-2.9449439	1.8675734	-1.1495766
C	-4.4593705	-1.6241397	-1.0551124
H	-5.0970794	-2.2286444	-1.7217522
H	-5.0853343	-0.8167816	-0.6661709
C	-3.9715647	-2.5021853	0.0906397
H	-4.8306222	-3.0281477	0.5391772
H	-3.2942716	-3.2688913	-0.2985908
C	-4.1565839	-0.9432758	1.9506766
H	-4.6641478	-1.5968859	2.6797641
H	-4.9352862	-0.5319034	1.3031617
C	-3.4565822	0.1846288	2.6998825
H	-4.1756646	0.6620715	3.3866304
H	-2.6488176	-0.2266348	3.3114163
C	-3.8581488	2.0572560	1.1752433
H	-4.2224647	2.8011259	1.9038233
H	-4.7186151	1.4423621	0.8961870
C	-3.3234342	2.7889448	-0.0537407
H	-4.0747661	3.5212859	-0.3930644
H	-2.4219321	3.3469391	0.2134257
C	-4.1337883	1.3455545	-1.8562239
H	-4.5500945	2.1088061	-2.5349774
H	-4.9072222	1.1301077	-1.1141298
C	-3.8167869	0.0882380	-2.6622474
H	-4.7100308	-0.2025493	-3.2402296
H	-3.0252401	0.3073352	-3.3830049
C	-2.6233706	-2.0633978	-2.6039005
H	-1.6197853	-2.2246709	-2.1971156
H	-2.5124366	-1.7288699	-3.6384299
H	-3.1852945	-3.0046921	-2.6097716
C	-2.4474699	-2.6639955	1.9566722
H	-1.8513971	-2.1015795	2.6758526
H	-1.7539273	-3.2229614	1.3255335
H	-3.1123472	-3.3677156	2.4854393
C	-1.8434745	1.9723171	2.5350284
H	-1.3127868	2.6321908	1.8473966
H	-1.1095568	1.2843030	2.9587548
H	-2.3161995	2.5622931	3.3384924
C	-2.0459112	2.5634586	-2.0920432
H	-1.7259498	1.8746822	-2.8778108
H	-1.1572773	2.8781723	-1.5409845
H	-2.5445424	3.4290402	-2.5589828
Ca	1.5747399	-0.0964217	-0.0107232
H	0.0122749	1.5447331	-0.0241811
N	2.8888216	1.3138145	1.7236476
N	2.9232079	1.6952272	-1.2743721
N	3.2843360	-1.2520331	-1.6697170
N	3.2306814	-1.6454457	1.3108537
C	3.8777241	2.1387805	0.9958650
H	4.2421548	2.9591953	1.6369309
H	4.7437589	1.5117916	0.7661348
C	3.3080140	2.7319237	-0.2903132
H	4.0419007	3.4349056	-0.7190012
H	2.4021238	3.3006135	-0.0632495
C	4.1063490	1.0975952	-1.9262516
H	4.5345250	1.7915333	-2.6692151
H	4.8744868	0.9380323	-1.1645741
C	3.7758667	-0.2227557	-2.6150413
H	4.6655347	-0.5804821	-3.1594618
H	2.9893781	-0.0666752	-3.3585575
C	4.4041949	-1.8245506	-0.8905216
H	4.9877746	-2.5267723	-1.5090569
H	5.0811500	-1.0109554	-0.6166626
C	3.9275108	-2.5471242	0.3647000
H	4.7882784	-3.0380866	0.8482560
H	3.2226966	-3.3383666	0.0934438
C	4.2052468	-0.7890148	2.0248922
H	4.7459717	-1.3744193	2.7874883
H	4.9515770	-0.4480206	1.3024749
C	3.5497014	0.4136653	2.6946662
H	4.3076236	0.9523776	3.2875609
H	2.7797757	0.0742051	3.3926117
C	1.9137624	2.1725162	2.4244002
H	1.3475242	2.7536666	1.6949810
H	1.2060567	1.5306037	2.9524256
H	2.4129352	2.8442901	3.1430210
C	2.0107200	2.2746289	-2.2804249
H	1.6378442	1.4882896	-2.9410742
H	1.1534602	2.6977954	-1.7541092
H	2.5159667	3.0449174	-2.8867802
C	2.5937119	-2.3151666	-2.4282815
H	2.2507469	-3.0931986	-1.7445805
H	1.7128567	-1.8876357	-2.9109632
H	3.2624885	-2.7702337	-3.1777695
C	2.4535069	-2.4518494	2.2748289
H	1.9111181	-1.7943041	2.9548600
H	1.7092603	-3.0378031	1.7317810
H	3.1093781	-3.1250948	2.8520021
H	-0.0160746	-3.4608009	-0.7231867
H	-0.0406781	-0.7770669	1.3787083

H 0.0986907 -4.1742992 -0.5340629

3$^+$: triple Ca-H-Ca bridged dimer cation
Energy = -2744.156185184

Ca	-1.5716308	0.0891820	0.0848949
H	0.0041076	-1.5098879	0.0615552
N	-3.0537226	-1.9414594	0.7525198
N	-3.4551189	0.8406494	1.7635080
N	-3.0498412	1.9558290	-0.9879303
N	-2.6303284	-0.8690741	-2.0347526
C	-4.3045242	-1.4794507	1.3898280
H	-4.8155832	-2.3127207	1.9003573
H	-4.9803971	-1.1298392	0.6046122
C	-4.0521879	-0.3595273	2.3946163
H	-4.9961495	-0.1090615	2.9060316
H	-3.3555588	-0.7016373	3.1652315
C	-4.4883048	1.6007285	1.0246048
H	-5.1494093	2.1359427	1.7268887
H	-5.1149064	0.8842167	0.4866163
C	-3.8913544	2.6027903	0.0437245
H	-4.7073883	3.1853420	-0.4153219
H	-3.2569652	3.3146355	0.5791334
C	-3.8900834	1.2979506	-2.0148294
H	-4.3223928	2.0507219	-2.6954489
H	-4.7300442	0.8143065	-1.5083694
C	-3.1207248	0.2709573	-2.8403499
H	-3.7616040	-0.0748154	-3.6691480
H	-2.2380271	0.7417730	-3.2816115
C	-3.7183452	-1.7960998	-1.6607270
H	-4.0068814	-2.4280849	-2.5176783
H	-4.5993938	-1.2045853	-1.3961597
C	-3.3263546	-2.6976035	-0.4919425
H	-4.1174572	-3.4491416	-0.3317231
H	-2.4086865	-3.2388231	-0.7373561
C	-2.2817694	-2.7930537	1.6805162
H	-2.0789314	-2.2467935	2.6050255
H	-1.3242819	-3.0276610	1.2092311
H	-2.8318435	-3.7151650	1.9314086
C	-2.8327073	1.6880882	2.8008752
H	-2.3673225	2.5608218	2.3391792
H	-2.0445529	1.1199274	3.2990679
H	-3.5751423	2.0261979	3.5428043
C	-2.1704818	2.9662451	-1.6114362
H	-1.4937411	2.4778372	-2.3132592
H	-1.5551359	3.4256015	-0.8336817
H	-2.7591812	3.7472835	-2.1216684
C	-1.5709664	-1.5773960	-2.7815102
H	-1.1718330	-2.3889614	-2.1713114
H	-0.7573321	-0.8706648	-2.9615153
H	-1.9559507	-1.9810409	-3.7335666
Ca	1.5706277	0.0921143	-0.0779452
H	-0.0295800	0.8217873	-1.4190940
N	3.4472968	1.0730170	-1.6480831
N	3.1078139	-1.8256406	-0.9811249
N	2.6416893	-1.0929129	1.9265551
N	2.9930805	1.8246191	1.2065329
C	4.5029955	0.0564574	-1.8532994
H	5.1517672	0.3383851	-2.6994850
H	5.1368579	0.0424880	-0.9627214
C	3.9352306	-1.3351709	-2.1067691
H	4.7653639	-2.0283118	-2.3222756
H	3.2970076	-1.3202599	-2.9949507
C	3.9647567	-2.2569911	0.1474025
H	4.4407997	-3.2253098	-0.0820155
H	4.7721956	-1.5281536	0.2586443
C	3.1974844	-2.3818436	1.4597689
H	3.8599027	-2.8288464	2.2202730
H	2.3503548	-3.0604381	1.3279373
C	3.6867406	-0.2144647	2.4933834
H	3.9610353	-0.5410812	3.5106934
H	4.5874037	-0.3110685	1.8802235
C	3.2453431	1.2466926	2.5468062
H	4.0019476	1.8338581	3.0939680
H	2.3053287	1.3249683	3.0998597
C	4.2507438	2.1019785	0.4826994
H	4.7445969	3.0033497	0.8829307
H	4.9355674	1.2662565	0.6512666
C	4.0160568	2.2855846	-1.0141653
H	4.9620628	2.5780961	-1.4986325
H	3.3084233	3.1020461	-1.1818075
C	2.8188098	1.4334502	-2.9352424
H	2.3895305	0.5441393	-3.4012867
H	2.0027433	2.1346766	-2.7492288
H	3.5495625	1.8819261	-3.6285473
C	2.2626761	-2.9460011	-1.4428862
H	1.5834955	-3.2465872	-0.6445001
H	1.6498014	-2.6036058	-2.2802672
H	2.8769345	-3.8027734	-1.7678278
C	1.5764125	-1.3477352	2.9172068
H	1.1290796	-0.4016298	3.2251030
H	0.7943960	-1.9342385	2.4300098
H	1.9701840	-1.8844424	3.7973303
C	2.1913104	3.0572560	1.3511538
H	1.9928848	3.4896948	0.3673329
H	1.2324941	2.7853845	1.7991868
H	2.7167429	3.8070544	1.9657886
H	0.0230469	0.9199081	1.3745146

1m^{+}•H_2 : loose complex of H_2 and cation **1m**$^+$
48
Energy = -1372.890596600

Ca	0.0089320	0.0003560	1.2501960
H	0.5169060	-0.1058860	3.2751710
N	1.2684040	-1.7158170	-0.0325030
N	-1.7054210	-1.2629920	-0.1023480
N	-1.2190580	1.7262350	-0.1054140
N	1.7435710	1.2394190	-0.0405180
C	0.3906570	-2.1842020	-1.1365360
H	0.8042810	-3.0958830	-1.5930130
H	0.3865920	-1.4157130	-1.9140190

C	-1.0332370	-2.4659360	-0.6700290
H	-1.6149660	-2.8709440	-1.5110800
H	-1.0293080	-3.2311320	0.1110330
C	-2.1311440	-0.3427710	-1.1928530
H	-3.0290230	-0.7367020	-1.6912860
H	-1.3390330	-0.3195580	-1.9455530
C	-2.4193420	1.0690410	-0.6925820
H	-2.8239420	1.6688390	-1.5212370
H	-3.1869540	1.0433810	0.0861710
C	-0.2815670	2.1564700	-1.1791350
H	-0.6708320	3.0527040	-1.6842730
H	-0.2376200	1.3636960	-1.9301430
C	1.1175250	2.4451970	-0.6469600
H	1.7413920	2.8424380	-1.4606730
H	1.0760710	3.2155810	0.1277150
C	2.2517860	0.3248530	-1.0973470
H	3.1831880	0.7210140	-1.5282850
H	1.5154410	0.2989920	-1.9050650
C	2.5036510	-1.0846440	-0.5723520
H	2.9364020	-1.6993460	-1.3750090
H	3.2350270	-1.0586230	0.2397380
C	1.6363190	-2.8444010	0.8556090
H	0.7360370	-3.3062030	1.2668270
H	2.2401010	-2.4669130	1.6843580
H	2.2069550	-3.6097780	0.3105420
C	-2.8879320	-1.7047540	0.6763410
H	-3.4334620	-0.8382170	1.0539100
H	-2.5577480	-2.3084570	1.5252710
H	-3.5686190	-2.3031330	0.0540950
C	-1.6422600	2.9025960	0.6916280
H	-0.7727040	3.3683080	1.1602860
H	-2.3301930	2.5814440	1.4763860
H	-2.1441820	3.6497190	0.0603250
C	2.8446310	1.6479230	0.8641890
H	3.2898220	0.7678470	1.3329850
H	2.4438330	2.2912060	1.6512190
H	3.6275770	2.1924170	0.3172970
H	-1.7379150	0.3751640	3.1539220
H	-2.3487540	0.5024730	2.7341070

1m^{+}•(THF)$_{2}$: cation **1m**$^{+}$ with two THFs
72
Energy = -1836.931350232

Ca	0.0165545	-0.0044588	-0.3445284
H	-0.7621317	-0.5312136	-2.2868268
N	1.5902284	2.0707529	0.5160492
N	0.9652497	-0.4612683	2.1057774
N	1.4227582	-2.1406661	-0.3514153
N	2.0250712	0.4199802	-1.9154423
C	2.3570361	1.5864978	1.6877265
H	2.8201435	2.4350253	2.2168626
H	3.1767805	0.9613034	1.3268689
C	1.4999984	0.8047760	2.6690645
H	2.0888355	0.5997008	3.5776938
H	0.6400642	1.4065879	2.9791912
C	2.0514105	-1.4665989	1.9713144
H	2.3142579	-1.8798636	2.9579295
H	2.9428208	-0.9583108	1.5970451
C	1.6810745	-2.6079919	1.0318033
H	2.4808978	-3.3649547	1.0521990
H	0.7707371	-3.1022771	1.3834903
C	2.6970628	-1.8372053	-1.0509856
H	3.1819568	-2.7730439	-1.3711057
H	3.3748424	-1.3616880	-0.3374535
C	2.5003693	-0.9402305	-2.2680472
H	3.4456332	-0.8885316	-2.8328551
H	1.7418441	-1.3704984	-2.9279826
C	3.0952691	1.2229812	-1.2871791
H	3.8294809	1.5533327	-2.0395576
H	3.6355339	0.5907131	-0.5781547
C	2.5294005	2.4420847	-0.5720510
H	3.3568355	3.0553884	-0.1803811
H	1.9778818	3.0679542	-1.2788259
C	0.8231129	3.2731642	0.9027846
H	0.1340772	3.0344012	1.7146593
H	0.2431719	3.6245778	0.0482184
H	1.4941719	4.0796130	1.2385715
C	-0.0581407	-0.9650768	3.0472907
H	-0.3922357	-1.9553414	2.7419640
H	-0.9172907	-0.2893835	3.0503250
H	0.3464561	-1.0342651	4.0688974
C	0.6818369	-3.1786377	-1.1024012
H	0.4059171	-2.7875384	-2.0828862
H	-0.2390280	-3.4180502	-0.5677184
H	1.2825008	-4.0949869	-1.2127381
C	1.5605367	1.0866554	-3.1529722
H	1.2168926	2.0990274	-2.9267347
H	0.7159188	0.5164706	-3.5440685
H	2.3737482	1.1509517	-3.8935589
O	-2.0528980	-1.3313008	0.3430707
C	-2.3746317	-2.5378146	1.0969399
C	-3.1369108	-1.1269044	-0.6135819
C	-3.5022668	-2.5331033	-1.0508497
C	-3.4150252	-3.3197120	0.2706263
H	-1.4428991	-3.0921689	1.2323857
H	-2.7610707	-2.2471418	2.0792974
H	-3.9709489	-0.6239759	-0.1057449
H	-2.7204779	-0.5110776	-1.4134216
H	-4.4929279	-2.5891593	-1.5090201
H	-2.7574773	-2.8859381	-1.7714551
H	-4.3811622	-3.3177987	0.7826593
H	-3.1136994	-4.3591465	0.1207279
O	-1.6558997	1.7217910	-0.1931211
C	-2.0491047	2.4685493	-1.3927320
C	-3.1684086	3.4000345	-0.9406421
C	-3.8220968	2.6035050	0.1992255
C	-2.6130990	1.9751858	0.8803908
H	-2.3728925	1.7403184	-2.1424962
H	-1.1644012	2.9931983	-1.7639190
H	-3.8602562	3.6295600	-1.7541391

H	-2.7556232	4.3398327	-0.5592350
H	-4.3986046	3.2302021	0.8834900
H	-4.4805115	1.8278162	-0.2042263
H	-2.1512490	2.6632270	1.5986204
H	-2.8161571	1.0189160	1.3676253

1m$^+$•THF : cation **1m**$^+$ with one THF
59
Energy = -1604.322459658

Ca	0.2789719	0.1641078	-0.3038791
H	-0.7087164	0.0886280	-2.1818247
N	1.7907112	1.7057970	1.1357133
N	0.6547619	-1.0097720	1.9505034
N	1.3108044	-2.1243289	-0.7382585
N	2.4139285	0.5933881	-1.5434987
C	2.2853436	0.8745463	2.2638876
H	2.7175158	1.5180997	3.0456489
H	3.0986411	0.2469553	1.8918269
C	1.1985503	0.0073277	2.8892470
H	1.6025942	-0.4756181	3.7923666
H	0.3597058	0.6319951	3.2106206
C	1.6446472	-2.0966098	1.7294013
H	1.7001377	-2.7460333	2.6165172
H	2.6302676	-1.6411498	1.6079792
C	1.3125286	-2.9369809	0.5036403
H	2.0267593	-3.7711956	0.4300305
H	0.3179941	-3.3794706	0.6067918
C	2.7019236	-1.8742145	-1.1981173
H	3.1056441	-2.7741936	-1.6871308
H	3.3242259	-1.6895107	-0.3183790
C	2.7986278	-0.6996969	-2.1658826
H	3.8216043	-0.6476382	-2.5699022
H	2.1214171	-0.8566702	-3.0095189
C	3.4625442	1.0862114	-0.6205323
H	4.3245740	1.4783540	-1.1824395
H	3.8272726	0.2390422	-0.0333623
C	2.9338522	2.1743000	0.3070695
H	3.7529492	2.5417320	0.9438113
H	2.5806965	3.0272775	-0.2786387
C	1.0696758	2.8845850	1.6675028
H	0.2250142	2.5621647	2.2777294
H	0.6871980	3.4800318	0.8355109
H	1.7344656	3.5121600	2.2798593
C	-0.5874450	-1.5799229	2.5188869
H	-1.0424505	-2.2754243	1.8098136
H	-1.2953246	-0.7748303	2.7229208
H	-0.3869325	-2.1178830	3.4573940
C	0.5497721	-2.8332195	-1.7930778
H	0.5093711	-2.2185357	-2.6926483
H	-0.4753963	-2.9917342	-1.4499052
H	1.0096044	-3.8064428	-2.0242072
C	2.1502597	1.5839212	-2.6122053
H	1.8634704	2.5423120	-2.1723253
H	1.3185496	1.2215242	-3.2202557
H	3.0410635	1.7400497	-3.2391704
O	-1.5685182	1.5024344	0.4357105
C	-2.6679716	1.2818164	1.3790583
C	-1.9777333	2.5022147	-0.5602431
C	-3.4956816	2.5127923	-0.4955308
C	-3.7485662	2.2935885	1.0040720
H	-3.0126050	0.2501228	1.2530219
H	-2.2772431	1.4189424	2.3912916
H	-1.5486596	3.4690189	-0.2708210
H	-1.5710157	2.1661746	-1.5191743
H	-3.9177091	3.4499679	-0.8656209
H	-3.9034676	1.6839951	-1.0833905
H	-3.6040165	3.2288318	1.5541280
H	-4.7497245	1.9142432	1.2208362

1m$^+$: L4-stabilzed calcium hydride cation
46
Energy = -1371.697634832

Ca	0.9180032	-1.0609235	-0.0143347
H	2.2327144	-2.6290033	-0.0501404
N	1.2511227	0.9966016	-1.4047784
N	-1.0200334	-0.9666963	-1.5963428
N	-1.1464061	-1.1134148	1.4027712
N	1.1229417	0.8482318	1.5968263
C	-0.0541549	1.3013959	-2.0475050
H	0.0779844	2.0467585	-2.8462371
H	-0.7024609	1.7570635	-1.2942365
C	-0.7245753	0.0606262	-2.6309253
H	-1.6427836	0.3608399	-3.1571645
H	-0.0711716	-0.4058909	-3.3732449
C	-2.2169798	-0.5865986	-0.8010995
H	-3.1340474	-0.7567619	-1.3851099
H	-2.1631390	0.4874576	-0.6034672
C	-2.3146629	-1.3551340	0.5139062
H	-3.2534465	-1.0860252	1.0202435
H	-2.3559214	-2.4302555	0.3186316
C	-1.2547461	0.2115553	2.0675486
H	-2.0013071	0.1740450	2.8751857
H	-1.6230214	0.9294056	1.3297551
C	0.0758809	0.6897619	2.6418352
H	-0.0832940	1.6346023	3.1822075
H	0.4525066	-0.0333769	3.3704438
C	0.9074882	2.0973992	0.8203032
H	1.2120038	2.9730058	1.4131833
H	-0.1653816	2.1982483	0.6358089
C	1.6673608	2.1043256	-0.5032556
H	1.5294304	3.0796896	-0.9927893
H	2.7396243	1.9887372	-0.3221739
C	2.2956426	0.7668774	-2.4294613
H	2.0028767	-0.0574245	-3.0836532
H	3.2327684	0.4987183	-1.9348138
H	2.4576641	1.6637526	-3.0446690
C	-1.2203628	-2.2841709	-2.2431886
H	-1.4244587	-3.0450251	-1.4865168
H	-0.3092313	-2.5653942	-2.7776083
H	-2.0603247	-2.2587466	-2.9523365

C	-1.0517500	-2.1963340	2.4089695
H	-0.1862997	-2.0321536	3.0549299
H	-0.9230800	-3.1534267	1.8968301
H	-1.9544714	-2.2418511	3.0351797
C	2.4626287	0.8518447	2.2286688
H	3.2365845	0.9510607	1.4641551
H	2.6158129	-0.0942850	2.7540833
H	2.5664738	1.6808873	2.9435867

1^{2+}**•(**THF)$_2$: cation dimer with two THFs
118
Energy = -3208.662083857

Ca	-3.6231949	1.6730924	-1.7451178
N	-4.8470917	2.2804230	0.4869791
N	-4.1310102	4.2680475	-1.6153279
N	-5.2824777	2.3788971	-3.7510116
N	-5.9828959	0.3735729	-1.6513663
H	-3.0848149	0.0053588	-0.3295516
H	-1.4227779	1.4862177	-1.4991039
C	-5.4926388	3.6082296	0.3754124
C	-5.8372825	1.1979032	0.7090184
C	-3.9366422	2.2733765	1.6502219
C	-4.5413074	4.6283048	-0.2322749
C	-5.2286124	4.5987475	-2.5640077
C	-2.9380534	5.0715560	-1.9616420
C	-5.1273815	3.8494539	-3.8854690
C	-6.6714652	2.0275518	-3.3715083
C	-4.9888319	1.7713780	-5.0697994
C	-6.7734948	0.5897411	-2.8883292
C	-6.7260178	0.9278299	-0.4916514
C	-5.7931466	-1.0776756	-1.4495722
Ca	-0.8364973	-0.1311758	-0.1043765
H	-5.8336419	3.9657781	1.3595871
H	-6.3826118	3.5133075	-0.2508438
H	-6.4701947	1.4329216	1.5802804
H	-5.2577339	0.3011499	0.9472952
H	-3.2756251	3.1384916	1.6173947
H	-3.3386778	1.3610184	1.6091760
H	-4.5024402	2.3070019	2.5941691
H	-5.0061092	5.6258568	-0.2161510
H	-3.6295001	4.6969583	0.3687261
H	-5.2309684	5.6799717	-2.7702306
H	-6.1791270	4.3760932	-2.0753015
H	-2.6933268	4.9366443	-3.0145476
H	-2.0886142	4.7408945	-1.3581097
H	-3.1137899	6.1422079	-1.7789605
H	-5.8836204	4.2562767	-4.5767359
H	-4.1488606	4.0225921	-4.3429396
H	-7.3531866	2.1798048	-4.2233953
H	-7.0007270	2.7042377	-2.5799117
H	-5.0220687	0.6816045	-4.9998200
H	-3.9909675	2.0747071	-5.3831533
H	-5.7263276	2.0936503	-5.8212208
H	-7.8315943	0.3296538	-2.7280002
H	-6.3901911	-0.0945881	-3.6497761
H	-7.5237555	0.2299988	-0.1922628
H	-7.2235436	1.8472962	-0.8076827
H	-5.1523341	-1.2381549	-0.5800633
H	-5.3038887	-1.5001280	-2.3302592
H	-6.7568341	-1.5904997	-1.3033990
N	-1.7674102	-1.9007693	1.5571183
N	1.4137785	-1.0761748	-1.0657679
N	-1.2431684	-2.3733541	-1.4281072
N	0.9307108	-0.6733238	1.9558130
C	-2.8039286	-1.2888359	2.4144220
C	-2.4176700	-2.9296048	0.7088810
C	-0.7144815	-2.5061300	2.4048775
C	1.2406889	-2.4977726	-1.4598725
C	2.4579276	-0.9616281	-0.0128351
C	1.8478654	-0.3066085	-2.2520839
C	-1.5192017	-3.4242032	-0.4156049
C	-0.0492327	-2.7301351	-2.2304151
C	-2.4130676	-2.2595835	-2.3202850
C	0.2185827	-1.4601600	2.9940354
C	2.0030914	-1.4946147	1.3399150
C	1.5539408	0.4894675	2.6261670
H	-2.3673261	-0.5008547	3.0332655
H	-3.5597102	-0.8478226	1.7617372
H	-3.2657755	-2.0364682	3.0780665
H	-2.7399564	-3.7883886	1.3195208
H	-3.3127530	-2.4613265	0.2894327
H	-1.1663478	-3.0881012	3.2238833
H	-0.1448004	-3.2123566	1.7969529
H	2.0958205	-2.8291092	-2.0692832
H	1.2432877	-3.1092370	-0.5554488
H	3.3717071	-1.4943564	-0.3202658
H	2.7183935	0.0974413	0.0722675
H	1.0676191	-0.3270500	-3.0156264
H	2.0290178	0.7295643	-1.9678137
H	2.7716058	-0.7231818	-2.6814915
H	-1.9937034	-4.2929666	-0.8982622
H	-0.5667254	-3.7748712	-0.0122769
H	-0.0912126	-3.7804199	-2.5590962
H	-0.0676327	-2.1157760	-3.1357633
H	-3.2688936	-1.9243326	-1.7371660
H	-2.2078223	-1.5147784	-3.0904129
H	-2.6405229	-3.2203844	-2.8084006
H	0.9356307	-1.9559215	3.6682837
H	-0.3483263	-0.7503704	3.6036162
H	2.8748213	-1.5434991	2.0114459
H	1.6381405	-2.5184670	1.2346556
H	2.1303803	1.0725695	1.9090829
H	0.7749232	1.1299559	3.0448132
H	2.2188277	0.1577762	3.4393514
H	0.7596379	2.4840309	-1.5090191
C	1.1227471	2.7652182	-0.5157470
C	0.9573388	4.2682161	-0.2404166
O	0.3044512	2.0628610	0.4691708
H	2.1509657	2.4211338	-0.3723926
C	0.2687521	4.3129289	1.1347441

H	0.3160062	4.7234665	-1.0013085
H	1.9151246	4.7918563	-0.2499909
C	-0.5566286	3.0375006	1.0996906
H	1.0035392	4.2626458	1.9437208
H	-0.3435729	5.2075213	1.2745124
H	-1.4556673	3.1569753	0.4781012
H	-0.8336875	2.6434102	2.0806617
H	-1.2025198	0.2317661	-3.5866482
C	-1.6503571	0.7536969	-4.4449797
C	-0.5925727	1.1377482	-5.4666300
O	-2.1872684	2.0117868	-3.9729521
H	-2.4839169	0.1723666	-4.8477975
C	0.0528446	2.3550770	-4.7849195
H	-1.0645218	1.4210834	-6.4124423
H	0.1181960	0.3299758	-5.6603480
C	-1.1163506	3.0057324	-4.0337430
H	0.8198482	2.0271727	-4.0769905
H	0.5158532	3.0441097	-5.4942812
H	-0.8526628	3.2748098	-3.0075645
H	-1.5209537	3.8727462	-4.5636844

1^{2+}•THF : cation dimer with one THF
105
Energy = -2976.072868336

Ca	-1.7411574	-0.0216885	0.1275703
H	0.0124080	-0.3437867	-1.2671951
N	-1.6110930	-2.5615533	0.3614107
N	-2.7002087	-0.6691504	2.4331883
N	-4.3168397	0.7632066	0.3514812
N	-3.1729114	-1.0900137	-1.7535998
C	-2.4476852	-2.9798130	1.5103720
H	-2.2110279	-4.0141690	1.8057280
H	-3.4934998	-2.9738061	1.1936292
C	-2.2578749	-2.0579165	2.7112593
H	-2.7947010	-2.4735647	3.5782736
H	-1.1994430	-2.0077660	2.9818074
C	-4.1796141	-0.5860463	2.4598274
H	-4.5471327	-0.6118163	3.4977923
H	-4.5814170	-1.4727293	1.9638094
C	-4.7016290	0.6724397	1.7827628
H	-5.7969931	0.7133719	1.8950828
H	-4.2996381	1.5599077	2.2804471
C	-5.1112933	-0.1935430	-0.4585030
H	-6.1437483	0.1716645	-0.5778742
H	-5.1743419	-1.1370538	0.0886769
C	-4.5020953	-0.4334521	-1.8334042
H	-5.1985611	-1.0326985	-2.4403625
H	-4.3655780	0.5177798	-2.3556910
C	-3.3385299	-2.5331115	-1.4448930
H	-3.6645137	-3.0789019	-2.3442029
H	-4.1395828	-2.6360024	-0.7086163
C	-2.0624188	-3.1699416	-0.9129014
H	-2.2173815	-4.2546467	-0.8014183
H	-1.2502049	-3.0278582	-1.6320211
C	-0.2083452	-2.9341122	0.6032273
H	0.1479732	-2.4315435	1.5027293
H	0.3806793	-2.5925401	-0.2516599
H	-0.0854370	-4.0229376	0.7179251
C	-2.1219375	0.2385260	3.4499637
H	-2.4586776	1.2624896	3.2678317
H	-1.0339170	0.2126158	3.3572241
H	-2.4293334	-0.0527798	4.4661583
C	-4.6039631	2.1384916	-0.1086347
H	-4.3572077	2.2419576	-1.1662425
H	-4.0014580	2.8464391	0.4625138
H	-5.6685742	2.3856538	0.0251492
C	-2.4702701	-0.9306680	-3.0466859
H	-1.4684123	-1.3560242	-2.9752882
H	-2.3653213	0.1344439	-3.2680767
H	-3.0277127	-1.4130382	-3.8646822
Ca	1.8050663	-0.0799158	-0.0276407
H	0.1412802	0.1724027	1.3712827
N	3.3030216	1.4867325	1.3356069
N	3.0417562	1.4847609	-1.6264031
N	3.1340475	-1.5710888	-1.6414263
N	3.4155320	-1.5618380	1.3441292
C	4.2918973	2.1368747	0.4395719
H	4.7596415	2.9977853	0.9408024
H	5.0911133	1.4202659	0.2364138
C	3.6655955	2.6010333	-0.8692814
H	4.4285650	3.1105510	-1.4770416
H	2.8815173	3.3372937	-0.6678601
C	4.0883702	0.6605948	-2.2852849
H	4.5074110	1.2028830	-3.1468099
H	4.9069091	0.5183479	-1.5751399
C	3.5655060	-0.6897293	-2.7583438
H	4.3428554	-1.1840138	-3.3609914
H	2.6978058	-0.5495080	-3.4088620
C	4.3099596	-2.1316942	-0.9276565
H	4.7533237	-2.9571874	-1.5047710
H	5.0731077	-1.3528620	-0.8606977
C	3.9531272	-2.6355566	0.4661196
H	4.8403858	-3.1041827	0.9187097
H	3.1839324	-3.4105950	0.3977562
C	4.5109909	-0.6557768	1.7807380
H	5.1361596	-1.1589691	2.5341991
H	5.1547358	-0.4557086	0.9206717
C	3.9923596	0.6557494	2.3561628
H	4.8296531	1.2098322	2.8068921
H	3.2725702	0.4589881	3.1551919
C	2.4830592	2.5096741	2.0212051
H	2.0166728	3.1645469	1.2823091
H	1.6956507	2.0111200	2.5910715
H	3.0955459	3.1287190	2.6935575
C	2.1250757	2.0376019	-2.6473124
H	1.5843665	1.2298880	-3.1435395
H	1.3970184	2.6829000	-2.1528545
H	2.6702355	2.6284486	-3.3984542
C	2.3160833	-2.6685606	-2.2064390
H	2.0748129	-3.3935025	-1.4265583

H	1.3872772	-2.2493146	-2.6017877
H	2.8569655	-3.1934218	-3.0078936
C	2.7926041	-2.1765948	2.5392511
H	2.2617118	-1.4185994	3.1194666
H	2.0781472	-2.9372002	2.2219684
H	3.5487211	-2.6542053	3.1799095
O	-1.4638330	2.3095127	-0.3912529
C	-1.0938670	3.3305239	0.5798631
C	-0.3125419	4.3576903	-0.2240928
C	-1.0625390	4.3542789	-1.5663219
C	-1.4842949	2.8912272	-1.7339593
H	-0.5253591	2.8246931	1.3635813
H	-2.0050118	3.7673652	1.0076973
H	0.7228497	4.0268829	-0.3593041
H	-0.2987932	5.3399411	0.2526462
H	-1.9434508	4.9996227	-1.5045236
H	-0.4463078	4.6991625	-2.3997112
H	-2.4937494	2.7895107	-2.1414213
H	-0.7921434	2.3039160	-2.3431299

1^{2+} : L4-stabilized dimer of **1m**$^{+}$: two Ca-H-Ca
92
Energy = -2743.459630393

Ca	-1.1262716	1.3253763	0.0208447
H	1.0243308	0.8761373	-0.0700803
N	-0.8409973	3.3977678	-1.3753332
N	-3.0780712	1.4442053	-1.5753824
N	-3.1903580	1.2893972	1.4538635
N	-0.9607095	3.2469729	1.6511839
C	-2.1474808	3.7261287	-1.9988100
H	-2.0214186	4.4863684	-2.7848103
H	-2.7877678	4.1685007	-1.2309215
C	-2.8251033	2.4961411	-2.5951314
H	-3.7602516	2.7995657	-3.0888092
H	-2.1879544	2.0546810	-3.3661752
C	-4.2700937	1.7878090	-0.7558277
H	-5.1910028	1.6021541	-1.3294409
H	-4.2394022	2.8610068	-0.5488714
C	-4.3364493	1.0037878	0.5516257
H	-5.2920808	1.2215710	1.0521059
H	-4.3198567	-0.0696845	0.3441892
C	-3.3363137	2.6151658	2.1050030
H	-4.0928690	2.5735071	2.9034447
H	-3.7046701	3.3220399	1.3567371
C	-2.0182612	3.1157459	2.6881076
H	-2.1879166	4.0735922	3.2017649
H	-1.6502172	2.4129736	3.4406307
C	-1.1588250	4.4902438	0.8605779
H	-0.8447237	5.3668948	1.4474115
H	-2.2301382	4.6036656	0.6741335
C	-0.3959029	4.4818036	-0.4609335
H	-0.5009196	5.4663287	-0.9413153
H	0.6708724	4.3278798	-0.2761716
C	0.1912117	3.1728723	-2.4118430
H	-0.1218766	2.3690568	-3.0830980
H	1.1208456	2.8721679	-1.9232661
H	0.3635826	4.0794366	-3.0108336
C	-3.2715690	0.1357962	-2.2409374
H	-3.3966000	-0.6479236	-1.4916720
H	-2.3829297	-0.0984585	-2.8329439
H	-4.1491463	0.1493683	-2.9043649
C	-3.0846616	0.2171789	2.4686238
H	-2.2352085	0.4102842	3.1287284
H	-2.9158988	-0.7336495	1.9583835
H	-3.9955143	0.1535736	3.0823777
C	0.3742643	3.2500045	2.2913198
H	1.1535635	3.2793471	1.5275176
H	0.4962142	2.3284567	2.8667981
H	0.4941812	4.1106647	2.9660637
Ca	1.1285563	-1.3232950	-0.0217025
H	-1.0218011	-0.8738979	0.0680012
N	0.8388579	-3.3937144	1.3764413
N	3.0803179	-1.4450934	1.5743100
N	3.1927598	-1.2927264	-1.4548443
N	0.9589817	-3.2458744	-1.6503639
C	2.1445073	-3.7245827	2.0003769
H	2.0164772	-4.4835281	2.7873029
H	2.7836734	-4.1695647	1.2330662
C	2.8252821	-2.4955472	2.5951369
H	3.7599270	-2.8006588	3.0886844
H	2.1895262	-2.0519501	3.3661159
C	4.2713413	-1.7922681	0.7549223
H	5.1928466	-1.6088333	1.3282981
H	4.2377353	-2.8654795	0.5484925
C	4.3395729	-1.0090314	-0.5529031
H	5.2946976	-1.2291360	-1.0533374
H	4.3253333	0.0645776	-0.3459568
C	3.3359096	-2.6194971	-2.1046001
H	4.0926955	-2.5802674	-2.9029304
H	3.7025583	-3.3264007	-1.3554998
C	2.0168539	-3.1177683	-2.6873613
H	2.1844557	-4.0763855	-3.2002917
H	1.6503530	-2.4148078	-3.4403946
C	1.1545662	-4.4886820	-0.8583731
H	0.8391962	-5.3653704	-1.4444427
H	2.2256113	-4.6037291	-0.6713075
C	0.3911421	-4.4772801	0.4627637
H	0.4932831	-5.4617327	0.9439176
H	-0.6751239	-4.3205791	0.2773693
C	-0.1929729	-3.1656032	2.4126584
H	0.1219767	-2.3620073	3.0833261
H	-1.1217987	-2.8630653	1.9236875
H	-0.3675101	-4.0712709	3.0123513
C	3.2766683	-0.1364361	2.2385823
H	3.4030700	0.6464234	1.4886280
H	2.3886298	0.1000580	2.8306396
H	4.1544053	-0.1511255	2.9017527
C	3.0894295	-0.2212941	-2.4706068
H	2.2395552	-0.4131975	-3.1305229
H	2.9227379	0.7304135	-1.9613148

H	4.0004085	-0.1602636	-3.0844783
C	-0.3758544	-3.2470914	-2.2908028
H	-1.1554443	-3.2738633	-1.5271685
H	-0.4958076	-2.3260724	-2.8676098
H	-0.4972248	-4.1084051	-2.9644767

2m^{+} : L5-stabilized calcium hydride cation
57
Energy = -1545.106390942

Ca	0.3379845	-0.0042645	-0.3256992
H	-0.9562756	-0.1521108	-1.9746985
N	-0.1452838	-2.3161177	0.7159503
N	-0.0674462	0.2722714	2.2099956
N	-0.1699409	2.4762382	0.2146350
N	2.3955058	1.3570917	-0.8314048
N	2.2816553	-1.5642713	-0.9066481
C	-1.4601584	-2.7647706	0.2003789
C	0.9172729	-3.2736456	0.3099495
C	-0.1861986	-2.2125373	2.1965177
C	-0.8355494	-0.9169936	2.6587836
C	-0.8921605	1.4950836	2.3849682
C	1.1816894	0.3840383	2.9934103
C	-0.2808180	2.6965286	1.6786386
C	0.9242228	3.3042432	-0.3522384
C	-1.4541543	2.8099656	-0.4437276
C	2.2868451	2.6580095	-0.1242938
C	2.6120831	1.5869939	-2.2766336
C	3.4842053	0.5355467	-0.2479762
C	2.2720207	-2.5905674	0.1683831
C	2.0332371	-2.1896437	-2.2279161
C	3.5857433	-0.8361189	-0.9100923
H	-1.4398890	-2.7602852	-0.8914761
H	-1.7081817	-3.7729723	0.5641593
H	-2.2409060	-2.0738463	0.5276953
H	0.9997733	-4.0948461	1.0359693
H	0.6233109	-3.7240431	-0.6402483
H	-0.7309728	-3.0676603	2.6244363
H	0.8396742	-2.2730057	2.5688247
H	-0.9338441	-0.9260909	3.7553567
H	-1.8453002	-0.8330409	2.2449148
H	-1.8836879	1.2820428	1.9721923
H	-1.0269615	1.7256720	3.4532342
H	1.7544482	1.2542027	2.6696883
H	0.9675915	0.4860671	4.0679324
H	1.8010399	-0.5025367	2.8449230
H	0.7174666	2.8972100	2.0748243
H	-0.8884590	3.5893981	1.8876643
H	0.7327679	3.4146692	-1.4228687
H	0.9182057	4.3164230	0.0788449
H	-2.2591071	2.2199209	0.0016693
H	-1.6952059	3.8774382	-0.3284828
H	-1.3937402	2.5556518	-1.5040131
H	2.4404110	2.4688714	0.9433390
H	3.0838335	3.3460219	-0.4442289
H	1.8474733	2.2652949	-2.6570250
H	3.6032393	2.0263380	-2.4640840
H	2.5269544	0.6508378	-2.8294823
H	3.2746996	0.4262490	0.8233759
H	4.4576694	1.0440840	-0.3357758
H	3.0401413	-3.3553493	-0.0283034
H	2.5453150	-2.0928291	1.1065205
H	2.1545017	-1.4364506	-3.0080789
H	2.7295511	-3.0193883	-2.4199358
H	1.0081617	-2.5602912	-2.2860776
H	3.9161466	-0.7250581	-1.9448178
H	4.3576852	-1.4218928	-0.3957292

2m^{+}•THF : THF-ccordinated cation **2m**$^{+}$
70
Energy = -1777.709132926

Ca	0.4915680	-0.1222376	-0.5048312
H	-0.3712828	-0.3186871	-2.4162630
N	0.0820474	-2.4477625	0.5617900
N	-0.1441748	0.1854751	1.9851566
N	-0.1801752	2.3459417	-0.0650127
N	2.5239307	1.3520470	-0.8055519
N	2.6066231	-1.5680950	-0.8223059
C	-1.1390263	-3.0105112	-0.0619253
C	1.2438517	-3.3369166	0.3028337
C	-0.1166740	-2.3021800	2.0265107
C	-0.8897496	-1.0392796	2.3736391
C	-1.0562502	1.3563198	2.0414032
C	1.0035495	0.3869804	2.8936831
C	-0.4483960	2.5819619	1.3755549
C	0.9151665	3.2267437	-0.5385185
C	-1.4097229	2.5855345	-0.8539174
C	2.2805546	2.6647072	-0.1585354
C	2.8565044	1.5498477	-2.2333013
C	3.6010386	0.6175936	-0.0999180
C	2.5548745	-2.5623806	0.2802225
C	2.5478304	-2.2471612	-2.1387447
C	3.8543559	-0.7549844	-0.7159788
H	-1.0068311	-3.0382199	-1.1466677
H	-1.3428926	-4.0267056	0.3076718
H	-1.9922800	-2.3677340	0.1643549
H	1.3037866	-4.1303429	1.0616057
H	1.0846602	-3.8325490	-0.6568719
H	-0.6491617	-3.1786733	2.4262093
H	0.8673658	-2.2872850	2.5021241
H	-1.1142776	-1.0312451	3.4519373
H	-1.8397642	-1.0240708	1.8321655
H	-1.9727812	1.0692010	1.5186800
H	-1.3202532	1.5956447	3.0836943
H	1.5530245	1.2871935	2.6146093
H	0.6696062	0.4906785	3.9373409
H	1.6894957	-0.4597395	2.8307155
H	0.4917771	2.8508582	1.8627332
H	-1.1259518	3.4396319	1.5032063
H	0.8278801	3.3059735	-1.6252253
H	0.8082808	4.2447358	-0.1343560

H	-2.2066059	1.9382593	-0.4810540
H	-1.7327223	3.6355125	-0.7833340
H	-1.2210028	2.3277215	-1.8989564
H	2.3384998	2.5161343	0.9248593
H	3.0683980	3.3858047	-0.4247898
H	2.0959209	2.1752371	-2.7015086
H	3.8381280	2.0340018	-2.3483603
H	2.8637084	0.5947451	-2.7592612
H	3.2964889	0.5161454	0.9485856
H	4.5440296	1.1881014	-0.1093631
H	3.3911907	-3.2742240	0.1909663
H	2.6911411	-2.0209563	1.2238633
H	2.6888182	-1.5075333	-2.9281824
H	3.3240361	-3.0222771	-2.2279674
H	1.5670065	-2.7013369	-2.2890775
H	4.2759384	-0.6388221	-1.7164503
H	4.6090034	-1.2799330	-0.1167801
C	-3.5436031	-0.5486582	-1.4502044
O	-3.2182555	-0.3024453	-0.0590621
C	-4.4879647	-0.0209765	0.5680845
C	-5.2396449	0.8721108	-0.4309646
C	-4.6127013	0.4971780	-1.8038116
H	-2.6097757	-0.4652373	-2.0135343
H	-3.9430341	-1.5692318	-1.5443090
H	-5.0232866	-0.9659695	0.7404628
H	-4.2839252	0.4545058	1.5305428
H	-6.3165927	0.6888139	-0.3992822
H	-5.0705678	1.9283612	-0.2044505
H	-5.3515794	0.0899690	-2.4984689
H	-4.1575727	1.3732913	-2.2731563

$\mathbf{2}^{2+}$: L5-stabilized dimer of $\mathbf{2m}^{+}$: two Ca-H-Ca
114
Energy = -3090.237748892

C	1.9284644	-3.7794263	-0.4250921
H	3.6943070	-3.8564172	0.8030870
N	1.1754170	-2.8244149	-1.2729705
H	2.3639448	-4.5852240	-1.0352171
H	1.2205714	-4.2549010	0.2575947
C	3.0354091	-3.0915961	0.3606193
Ca	0.9911075	-0.4121431	-0.0633807
C	-0.1628450	-3.3753405	-1.5736846
C	1.9029866	-2.6215216	-2.5526795
N	2.5209328	-2.1921530	1.4268802
H	3.6553075	-2.4899707	-0.3144629
Ca	-2.5042960	0.4141745	0.2995751
N	2.1310268	-0.1449320	-2.4042992
N	1.1824710	2.1543668	-0.7439280
N	2.7721094	0.7629817	1.3685686
H	-1.0133286	-0.5973955	-0.9674830
H	-0.4802416	0.5716221	1.2552919
H	-0.7165303	-3.5127067	-0.6424924
H	-0.0863606	-4.3453816	-2.0898889
H	-0.7157358	-2.6676172	-2.1927006
C	1.5784566	-1.2800470	-3.1860322
H	1.6542785	-3.4292238	-3.2578333
H	2.9750177	-2.6982556	-2.3575918
C	1.8883185	-3.0029999	2.4923602
C	3.6726484	-1.4641128	2.0353021
N	-2.5949549	-1.4057958	2.1815030
N	-4.3657550	-1.3595198	-0.2465916
N	-3.4573811	0.7198630	-2.3031424
N	-4.0170502	2.5655426	0.0017252
N	-3.1023073	1.5860983	2.6410949
C	1.5749576	1.1167155	-2.9535389
C	3.6046195	-0.1289552	-2.5145358
C	1.8610766	2.3066173	-2.0525666
C	1.9176713	2.8560369	0.3323494
C	-0.1839440	2.6903575	-0.8289385
C	3.1418705	2.0703431	0.7774393
C	2.2484691	0.9632013	2.7364670
C	3.9575883	-0.1253720	1.3740790
H	1.9603620	-1.2565420	-4.2192863
H	0.4922477	-1.1454113	-3.2251470
H	1.5388512	-2.3399994	3.2863492
H	2.6009033	-3.7246689	2.9207507
H	1.0324569	-3.5532424	2.1014287
H	3.4488790	-1.3057289	3.0920674
H	4.5804573	-2.0831565	1.9974729
C	-1.3257577	-2.1512092	2.1497712
C	-2.7334724	-0.7397281	3.4985704
C	-3.7205513	-2.3396961	1.9445778
C	-3.9140731	-2.5858545	0.4575600
C	-4.2179960	-1.5760646	-1.7091116
C	-5.7828666	-1.1086010	0.0919795
C	-4.4992995	-0.3181777	-2.5135895
C	-4.0355112	2.0744061	-2.4468158
C	-2.3997961	0.5234842	-3.3127383
C	-4.8496278	2.4673182	-1.2237558
C	-3.2827349	3.8496726	-0.0209448
C	-4.9099573	2.5063446	1.1872149
C	-3.6592971	0.4650161	3.4385317
C	-1.9152289	2.1579624	3.3159003
C	-4.1463264	2.6370701	2.4948951
H	0.4934462	0.9738425	-3.0406674
H	1.9695716	1.3073960	-3.9643993
H	3.9227236	-0.0536449	-3.5658543
H	4.0285822	-1.0387480	-2.0871917
H	4.0139285	0.7199724	-1.9661444
H	2.9351288	2.4049496	-1.8822331
H	1.5375226	3.2321725	-2.5521042
H	1.2233822	2.9722217	1.1689929
H	2.2217195	3.8660878	0.0160229
H	-0.7495148	2.1296256	-1.5782742
H	-0.1914878	3.7572852	-1.1023521
H	-0.6541312	2.5563744	0.1488832
H	3.7957698	1.8733112	-0.0780517
H	3.7268746	2.6705416	1.4920358
H	1.8110771	0.0374287	3.1148829
H	1.4512966	1.7047710	2.7088071

H	3.0422552	1.2974514	3.4231734
H	4.2730872	-0.2622616	0.3333118
H	4.8004011	0.3456457	1.9067017
H	-0.5135395	-1.4337941	2.2855325
H	-1.2884404	-2.9213485	2.9358200
H	-1.2090964	-2.6368534	1.1778682
H	-3.1047576	-1.4472087	4.2553578
H	-1.7364150	-0.4258648	3.8148221
H	-3.5439240	-3.2955716	2.4610364
H	-4.6254875	-1.9092445	2.3794435
H	-4.6300990	-3.4078216	0.3003591
H	-2.9641468	-2.8885252	0.0047992
H	-3.1837205	-1.8945384	-1.8813338
H	-4.8865266	-2.3854121	-2.0445794
H	-6.4180411	-1.9530813	-0.2170774
H	-5.8924304	-0.9662435	1.1680871
H	-6.1413670	-0.2058437	-0.4023582
H	-5.4734635	0.0943892	-2.2439730
H	-4.5596622	-0.5801014	-3.5803406
H	-3.2059134	2.7732732	-2.5901745
H	-4.6702103	2.1474168	-3.3445259
H	-1.9345755	-0.4517714	-3.1617839
H	-2.8048060	0.5936921	-4.3351512
H	-1.6322444	1.2904086	-3.1897210
H	-5.6264371	1.7204869	-1.0355668
H	-5.3680770	3.4198587	-1.4139074
H	-2.7556020	3.9507196	-0.9702077
H	-3.9707684	4.7019746	0.0889228
H	-2.5456176	3.8853693	0.7823914
H	-5.4431886	1.5493466	1.1503976
H	-5.6700206	3.3032833	1.1391688
H	-3.8738410	0.8034591	4.4657161
H	-4.6182337	0.1804377	2.9895237
H	-2.1473097	2.4652114	4.3481178
H	-1.1016906	1.4330793	3.3060052
H	-1.5792410	3.0326691	2.7534245
H	-3.6618615	3.6141984	2.5453930
H	-4.8589181	2.5996538	3.3304805

CH_2=CH_2 : ethylene substrate
6
Energy = -78.63740833188

C	-1.4501755	0.4945139	0.0000003
C	-0.1176829	0.4944971	0.0000082
H	-2.0220005	1.4196004	-0.0000137
H	-2.0220211	-0.4305622	-0.0000101
H	0.4541445	-0.4305917	-0.0000351
H	0.4541646	1.4195756	0.0000356

CH_2=CHBu : 1-hexene substrate
18
Energy = -235.9904743754

C	3.0775964	-0.0981642	-0.4543711
C	2.0354411	-0.2856764	0.3591128
H	3.0842558	0.7028831	-1.1918822
H	3.9582970	-0.7339727	-0.4121633
H	2.0682586	-1.1031350	1.0823091
C	0.7772023	0.5329051	0.3551433
C	-0.4729617	-0.3045008	0.0244202
H	0.8720775	1.3559226	-0.3644722
H	0.6352748	0.9855549	1.3479433
C	-1.7670800	0.5150377	0.0757769
H	-0.3554014	-0.7451775	-0.9747527
H	-0.5442341	-1.1435361	0.7306328
C	-3.0092301	-0.3180264	-0.2568930
H	-1.8746638	0.9543025	1.0769082
H	-1.6888235	1.3561852	-0.6264183
H	-3.9199914	0.2894259	-0.2136517
H	-2.9357068	-0.7444314	-1.2644410
H	-3.1236968	-1.1485795	0.4498899

C$^+$•H_2 : loose H_2 complex of **C**$^+$
113
Energy = -2981.357143812

Ca	1.7808267	-0.0109845	0.1349972
H	0.6061597	-1.6558142	-0.7775996
N	3.6321356	-1.9596766	0.1452782
N	3.1238616	0.1038634	-2.0652432
N	3.0816522	2.3412423	-0.0409027
N	3.4970960	0.2683037	2.1373147
C	4.5660766	-1.6090141	-0.9473051
H	5.2239029	-2.4642981	-1.1774313
H	5.2139757	-0.7988941	-0.6023237
C	3.8403403	-1.1835454	-2.2187437
H	4.5631518	-1.1311735	-3.0498146
H	3.0909293	-1.9338055	-2.4846138
C	4.0662670	1.2438900	-2.0807860
H	4.3812987	1.4735794	-3.1120688
H	4.9688334	0.9493821	-1.5391019
C	3.4757407	2.5067698	-1.4611048
H	4.2009487	3.3303655	-1.5733346
H	2.5727795	2.7989499	-2.0062845
C	4.2693118	2.2497427	0.8381079
H	4.7361614	3.2414416	0.9582208
H	5.0100258	1.6092509	0.3532798
C	3.9209730	1.6857208	2.2109098
H	4.7854576	1.8023394	2.8848824
H	3.0964673	2.2529650	2.6517920
C	4.6832142	-0.6014290	1.9656738
H	5.2248460	-0.7035932	2.9208398
H	5.3685697	-0.1105130	1.2703988
C	4.3285484	-1.9890573	1.4506710
H	5.2490454	-2.5954134	1.3988806
H	3.6599424	-2.4879633	2.1583965
C	3.0499413	-3.2914339	-0.1117518
H	2.4077681	-3.2512887	-0.9920910
H	2.4286263	-3.5719800	0.7394810
H	3.8395309	-4.0505258	-0.2456151
C	2.1283834	0.2327984	-3.1498090
H	1.6360958	1.2043738	-3.0878511

H	1.3693455	-0.5419931	-3.0149536
H	2.6029350	0.1340578	-4.1399279
C	2.2739589	3.5111649	0.3609985
H	1.9191784	3.3902983	1.3866464
H	1.4071970	3.5908983	-0.2956337
H	2.8629338	4.4408034	0.2993478
C	2.7759374	-0.0970046	3.3749439
H	2.4688758	-1.1436065	3.3285250
H	1.8698595	0.5065095	3.4534587
H	3.4103845	0.0520722	4.2644684
Ca	-1.3049307	-0.7300188	-0.0299414
H	0.1652549	-0.3219570	1.6325326
N	-3.1617318	-0.3665241	1.8198261
N	-1.5893699	-2.9133488	1.3291032
N	-2.1460488	-2.5977441	-1.6355586
N	-3.7511152	-0.1049693	-1.1492607
C	-3.5201415	-1.7002940	2.3567969
H	-4.0669771	-1.5918241	3.3085087
H	-4.2059792	-2.1808628	1.6545446
C	-2.3058409	-2.5915020	2.5813933
H	-2.6261088	-3.5093459	3.1036333
H	-1.5871265	-2.0809988	3.2287222
C	-2.3211497	-3.8957549	0.5022100
H	-2.1972023	-4.9147108	0.9062622
H	-3.3892985	-3.6673850	0.5543765
C	-1.8506148	-3.8774409	-0.9500744
H	-2.3030207	-4.7255764	-1.4912615
H	-0.7646507	-4.0019129	-0.9852650
C	-3.5834913	-2.4780164	-1.9508026
H	-3.8516582	-3.1220642	-2.8051751
H	-4.1558709	-2.8415262	-1.0936411
C	-3.9848764	-1.0445674	-2.2730392
H	-5.0441703	-1.0272864	-2.5795108
H	-3.4047630	-0.6767536	-3.1245883
C	-4.7706650	-0.3051620	-0.0922319
H	-5.7330769	0.1351306	-0.4017298
H	-4.9420964	-1.3781745	0.0204822
C	-4.3554756	0.2942430	1.2457796
H	-5.2090004	0.2461987	1.9426954
H	-4.1073853	1.3530622	1.1190963
C	-2.6058168	0.4729908	2.9025431
H	-1.6607008	0.0483269	3.2441667
H	-2.3948115	1.4685466	2.5066378
H	-3.3146430	0.5583565	3.7430761
C	-0.2501164	-3.4299742	1.6651954
H	0.2845645	-3.6610261	0.7447546
H	0.2986522	-2.6377163	2.1805317
H	-0.3178960	-4.3324909	2.2972008
C	-1.3399086	-2.5173245	-2.8711508
H	-1.5426701	-1.5762585	-3.3882126
H	-0.2855748	-2.5296199	-2.5830110
H	-1.5719704	-3.3503368	-3.5553677
C	-3.8633842	1.2695652	-1.6766402
H	-3.7682898	1.9901073	-0.8637112
H	-3.0586019	1.4524070	-2.3914762
H	-4.8339840	1.4265302	-2.1749278
C	-0.2247950	1.4054368	-0.9394261
C	-1.0321514	2.3043689	0.0071661
H	-0.8121971	1.2530915	-1.8689764
H	0.6524840	1.9750127	-1.2943359
H	-1.9384488	1.7680794	0.3556857
H	-0.4621678	2.4744485	0.9327385
C	-1.5130511	3.6664353	-0.5259684
H	-0.6365466	4.2860593	-0.7651063
H	-1.1932627	0.9699359	-4.9437399
H	-0.9625817	1.1139198	-4.2500579
H	-2.0413503	3.5152355	-1.4773313
C	-2.4196034	4.4198256	0.4533643
C	-2.9230360	5.7676963	-0.0738131
H	-1.8820495	4.5761625	1.4000526
H	-3.2870073	3.7866819	0.7006048
C	-3.8415409	6.4924403	0.9156646
H	-2.0604297	6.4056559	-0.3102041
H	-3.4567079	5.6059173	-1.0205305
H	-4.1881143	7.4509478	0.5137679
H	-4.7253338	5.8847752	1.1453064
H	-3.3198506	6.6913765	1.8595147

CH_3CH_2Bu : hexane product of hydrogenation
20
Energy = -237.2242871135

C	-0.2276111	3.2158786	0.0001266
C	0.5418781	1.8907454	-0.0002351
H	-0.8692495	3.2948331	0.8860027
H	0.4532592	4.0743590	-0.0002730
H	-0.8702376	3.2948127	-0.8850399
H	1.1983779	1.8470603	-0.8801441
H	1.1989657	1.8469541	0.8792311
C	-0.3796112	0.6655071	-0.0000609
C	0.3795764	-0.6655059	0.0001416
H	-1.0375813	0.7078890	-0.8802460
H	-1.0376079	0.7080721	0.8800966
C	-0.5418894	-1.8907611	-0.0000818
H	1.0377823	-0.7080218	-0.8798602
H	1.0373394	-0.7079278	0.8804805
C	0.2276384	-3.2158732	0.0000474
H	-1.1988821	-1.8470904	0.8794641
H	-1.1984851	-1.8469895	-0.8799170
H	-0.4531953	-4.0743757	-0.0003510
H	0.8701443	-3.2946715	-0.8852170
H	0.8693888	-3.2948946	0.8858358

CH_3CH_3 : ethane product of hydrogenation
8
Energy = -79.87656483085

C	-1.0051159	-0.6409098	-0.0084068
C	0.5272731	-0.6408594	-0.0083934
H	-1.4008064	0.3809238	-0.0085347
H	-1.4007168	-1.1519760	-0.8932916
H	-1.4007267	-1.1517491	0.8766047

H	0.9229636	-1.6626930	-0.0084846
H	0.9228896	-0.1298188	-0.8932860
H	0.9228684	-0.1299943	0.8766103

C^{+} : adduct of 1-hexene to cation **3**$^{+}$
111
Energy = -2980.175494740

Ca	1.7787830	-0.0111637	0.1312606
H	0.6073521	-1.6584247	-0.7825673
N	3.6334287	-1.9582112	0.1502454
N	3.1279652	0.1011665	-2.0632807
N	3.0787192	2.3428380	-0.0442128
N	3.4898192	0.2730478	2.1382936
C	4.5698762	-1.6080835	-0.9403426
H	5.2292548	-2.4629620	-1.1675885
H	5.2158421	-0.7966267	-0.5948708
C	3.8467170	-1.1851855	-2.2141478
H	4.5713956	-1.1324981	-3.0435876
H	3.0991171	-1.9368774	-2.4810257
C	4.0688097	1.2424186	-2.0797284
H	4.3850429	1.4703494	-3.1110733
H	4.9708746	0.9498297	-1.5361567
C	3.4764626	2.5061848	-1.4636500
H	4.2022700	3.3294051	-1.5751233
H	2.5751626	2.7978127	-2.0118253
C	4.2640226	2.2529643	0.8380656
H	4.7302527	3.2450027	0.9579881
H	5.0062070	1.6119823	0.3561341
C	3.9121595	1.6909207	2.2108064
H	4.7745804	1.8094591	2.8871248
H	3.0858124	2.2581982	2.6482024
C	4.6773745	-0.5956886	1.9715257
H	5.2163468	-0.6955532	2.9284493
H	5.3641930	-0.1051423	1.2774308
C	4.3259827	-1.9846019	1.4577045
H	5.2474579	-2.5898180	1.4096950
H	3.6558598	-2.4831886	2.1642088
C	3.0531697	-3.2908273	-0.1065186
H	2.4121431	-3.2520150	-0.9877642
H	2.4309530	-3.5713075	0.7440446
H	3.8437852	-4.0491725	-0.2386726
C	2.1319824	0.2270584	-3.1479276
H	1.6344843	1.1959200	-3.0837209
H	1.3766218	-0.5515733	-3.0133938
H	2.6071352	0.1316331	-4.1380535
C	2.2697915	3.5131467	0.3538957
H	1.9109521	3.3929558	1.3782016
H	1.4055867	3.5926014	-0.3061267
H	2.8591883	4.4426623	0.2939515
C	2.7653801	-0.0906535	3.3744502
H	2.4601583	-1.1378759	3.3293565
H	1.8579697	0.5114846	3.4486506
H	3.3968422	0.0614423	4.2656112
Ca	-1.3057948	-0.7319814	-0.0366834
H	0.1633616	-0.3266353	1.6252506
N	-3.1570726	-0.3657464	1.8180232
N	-1.5871717	-2.9154783	1.3239322
N	-2.1526233	-2.5990183	-1.6373703
N	-3.7555637	-0.1071872	-1.1491372
C	-3.5140257	-1.6993301	2.3563434
H	-4.0581272	-1.5905298	3.3096195
H	-4.2020348	-2.1798937	1.6561684
C	-2.2994710	-2.5910060	2.5778934
H	-2.6186303	-3.5077840	3.1027658
H	-1.5780991	-2.0798064	3.2217197
C	-2.3217937	-3.8977944	0.4995734
H	-2.1970664	-4.9167962	0.9033150
H	-3.3896941	-3.6689702	0.5548359
C	-1.8551505	-3.8793141	-0.9540858
H	-2.3093434	-4.7270955	-1.4943687
H	-0.7693342	-4.0039187	-0.9922185
C	-3.5908140	-2.4803283	-1.9493902
H	-3.8604071	-3.1251577	-2.8028045
H	-4.1608595	-2.8438594	-1.0906379
C	-3.9935726	-1.0473114	-2.2714958
H	-5.0540548	-1.0302773	-2.5740189
H	-3.4160049	-0.6802083	-3.1250249
C	-4.7722558	-0.3048866	-0.0891330
H	-5.7349890	0.1365969	-0.3961068
H	-4.9448464	-1.3776281	0.0248160
C	-4.3524139	0.2949429	1.2472458
H	-5.2037105	0.2476883	1.9469859
H	-4.1042129	1.3535772	1.1190996
C	-2.5977657	0.4744179	2.8986465
H	-1.6515077	0.0500381	3.2373943
H	-2.3874419	1.4696252	2.5015858
H	-3.3042663	0.5602600	3.7411642
C	-0.2475660	-3.4329149	1.6569353
H	0.2840914	-3.6664890	0.7353837
H	0.3035883	-2.6401058	2.1689006
H	-0.3144368	-4.3341087	2.2909755
C	-1.3482131	-2.5135969	-2.8738005
H	-1.5495231	-1.5687079	-3.3851411
H	-0.2935238	-2.5299276	-2.5870802
H	-1.5829589	-3.3422610	-3.5624324
C	-3.8657737	1.2669805	-1.6779113
H	-3.7660836	1.9883982	-0.8662579
H	-3.0626791	1.4455273	-2.3959601
H	-4.8376691	1.4259320	-2.1732263
C	-0.2251411	1.4034268	-0.9514106
C	-1.0315540	2.3010879	-0.0027872
H	-0.8084193	1.2527727	-1.8843101
H	0.6560041	1.9711851	-1.2996998
H	-1.9376401	1.7650068	0.3467423
H	-0.4604050	2.4689952	0.9224888
C	-1.5127824	3.6651260	-0.5307598
H	-0.6365387	4.2844572	-0.7715378
H	-2.0453255	3.5171173	-1.4802511
C	-2.4146860	4.4165138	0.4544382
C	-2.9158346	5.7683760	-0.0646851

H	-1.8740444	4.5667380	1.4003568
H	-3.2831167	3.7845468	0.7010765
C	-3.8306706	6.4901102	0.9304000
H	-2.0520633	6.4053988	-0.2993202
H	-3.4517773	5.6129638	-1.0111793
H	-4.1752546	7.4518119	0.5344515
H	-4.7157208	5.8835567	1.1581195
H	-3.3069502	6.6822640	1.8745275

eA^{+} : ethylene adduct of cation **1m**$^{+}$•THF
65
Energy = -1682.990680386

Ca	0.1270306	0.2944884	-0.1146315
N	-1.6836805	0.2435658	-1.8660040
N	-0.4697189	-2.2050755	-0.6929428
N	-0.6166720	-0.8577826	2.0705677
N	-1.8777382	1.5672629	0.8736498
C	-2.3433640	-1.0819286	-1.9165113
H	-2.9703272	-1.1687224	-2.8179228
H	-3.0138705	-1.1630379	-1.0572644
C	-1.3354928	-2.2253195	-1.9008362
H	-1.8733954	-3.1827716	-1.9820965
H	-0.6772830	-2.1568147	-2.7713916
C	-1.2232784	-2.7085385	0.4844168
H	-1.3036188	-3.8058749	0.4374095
H	-2.2427185	-2.3196161	0.4290561
C	-0.5874260	-2.3217914	1.8152622
H	-1.0971200	-2.8663429	2.6255603
H	0.4618821	-2.6306680	1.8314324
C	-1.9953977	-0.4092078	2.3910563
H	-2.2685591	-0.7144953	3.4131562
H	-2.6866815	-0.9171971	1.7145669
C	-2.1569630	1.0987797	2.2539912
H	-3.1712288	1.3858270	2.5719362
H	-1.4587883	1.6143706	2.9196472
C	-3.0287868	1.2791279	-0.0199209
H	-3.8362513	2.0068810	0.1562530
H	-3.4295365	0.2968268	0.2440484
C	-2.6490203	1.3105776	-1.4971592
H	-3.5635545	1.2359186	-2.1061736
H	-2.1745275	2.2656132	-1.7388895
C	-1.0749454	0.5673701	-3.1760300
H	-0.3391924	-0.1945424	-3.4450971
H	-0.5649068	1.5291565	-3.1005468
H	-1.8367179	0.6111031	-3.9693156
C	0.7031855	-3.0749647	-0.9312236
H	1.3753120	-3.0384482	-0.0724456
H	1.2427929	-2.7183263	-1.8109196
H	0.3951266	-4.1176685	-1.1034412
C	0.2842335	-0.5457427	3.2012884
H	0.3347553	0.5346498	3.3571134
H	1.2860068	-0.9150881	2.9755866
H	-0.0633835	-1.0168363	4.1331841
C	-1.6161679	3.0236799	0.9002826
H	-1.3819664	3.3771262	-0.1039472
H	-0.7534371	3.2211578	1.5400628
H	-2.4867783	3.5737915	1.2898233
C	3.1282386	-0.6157959	-1.1092694
O	2.4031041	-0.5205288	0.1582071
C	3.3256289	-0.0481380	1.1974041
C	4.6826362	0.1363076	0.5151227
C	4.3067284	0.3311648	-0.9621199
H	3.4486265	-1.6546057	-1.2484064
H	2.4354080	-0.3318701	-1.9069167
H	2.9268217	0.8938177	1.5872955
H	3.3469896	-0.7958730	1.9941566
H	5.2310443	0.9845942	0.9307592
H	5.2942940	-0.7629690	0.6360603
H	3.9842920	1.3584937	-1.1514356
H	5.1237579	0.0819946	-1.6433563
C	1.1046912	2.3668230	-1.1110456
C	1.9268903	3.3248280	-0.2206284
H	0.2908086	2.9593062	-1.5788652
H	1.7406045	2.0725243	-1.9723603
H	1.3275786	3.7034712	0.6208180
H	2.7901753	2.8142025	0.2304448
H	2.3356280	4.2178722	-0.7290089

eA^{2+} : ethylene adduct of cation dimer **1**$^{2+}$
98
Energy = -2822.125662920

Ca	0.0558882	-0.6480947	1.9059034
Ca	0.2249039	-0.6522880	-1.6034287
H	-1.0655721	-0.1435179	0.0966104
N	0.9801353	-2.3138747	3.7363064
N	0.5682484	-2.4738353	-3.4339565
N	-1.0203321	1.4608878	2.9245798
N	-0.1221515	1.6844746	-2.5479881
N	1.8033917	0.5878339	3.2274060
N	2.2965306	0.0023265	-2.9309681
N	-1.8811054	-1.4402103	3.3189401
N	-1.8649614	-0.7775021	-2.9928674
C	0.0513329	2.3656767	3.4131265
C	1.1572294	2.2194421	-3.0808872
C	-0.1983906	-3.0011279	4.3299262
C	-0.7530257	-2.7423779	-4.0652664
C	1.1849911	1.6175277	4.1017252
C	1.9688213	1.1560720	-3.8078478
C	-1.3897878	-2.0830856	4.5648508
C	-1.5868465	-1.4826881	-4.2679987
C	1.9570671	-3.3603485	3.3531218
C	1.1446008	-3.7674173	-2.9965874
C	-1.8838251	2.2102378	1.9853755
C	-0.6397723	2.6067663	-1.5156876
C	1.6218343	-1.4028041	4.7208613
C	1.4917095	-1.8424815	-4.4152436
C	-1.8420639	0.9534719	4.0525136
C	-1.1247855	1.5315967	-3.6353319
C	2.5534382	-0.3985890	4.0495434
C	2.6897741	-1.1741674	-3.7496963

C	-2.6988904	-0.2423224	3.6516446
C	-2.2875114	0.6252870	-3.2412147
C	2.7070285	1.2191734	2.2402714
C	3.4012037	0.3606615	-2.0131405
C	-2.7085909	-2.3910710	2.5407988
C	-2.9226103	-1.4798364	-2.2312137
H	-0.3524342	3.1205327	4.1054924
H	0.9784777	3.0679848	-3.7582909
H	0.0722957	-3.4792041	5.2844676
H	-0.6237038	-3.2467470	-5.0351814
H	0.4337787	2.9074125	2.5429592
H	1.7241801	2.6077181	-2.2298839
H	-0.4740670	-3.8052849	3.6404369
H	-1.2828524	-3.4416898	-3.4121131
H	1.5143944	-4.0166445	2.6026474
H	0.4623659	-4.2501147	-2.2955989
H	-1.2628390	2.6277695	1.1929854
H	0.1037052	2.7004870	-0.7193145
H	0.8370754	-0.8714073	5.2651622
H	0.9275759	-1.1061302	-4.9929005
H	-1.1712175	0.6747280	4.8695926
H	-0.6142358	1.1271662	-4.5133180
H	0.8134638	1.1176076	4.9992631
H	1.4071786	0.7823847	-4.6672545
H	-1.1221547	-1.2932955	5.2706189
H	-1.0656237	-0.7902379	-4.9336268
H	2.2451398	-3.9606660	4.2291984
H	1.3040578	-4.4352361	-3.8562545
H	-2.4092565	3.0291858	2.5003717
H	-0.8408186	3.6049043	-1.9333265
H	2.1857885	-1.9839015	5.4661652
H	1.8513362	-2.5965377	-5.1315602
H	-2.4951522	1.7524095	4.4365523
H	-1.5213057	2.5165950	-3.9256365
H	1.9434943	2.3418190	4.4346140
H	2.8896943	1.6101269	-4.2041545
H	-2.1964092	-2.6643642	5.0366556
H	-2.5271502	-1.7556864	-4.7711400
H	2.8541772	-2.9043462	2.9324623
H	2.1008440	-3.6040213	-2.4960008
H	-2.6110708	1.5402328	1.5258077
H	-1.5540331	2.1995748	-1.0834769
H	2.1491892	1.9408282	1.6365201
H	3.1090096	1.2121619	-1.3932310
H	-2.1409712	-3.3023679	2.3406285
H	-2.6255374	-2.5159896	-2.0511279
H	3.2509919	-0.9167334	3.3860050
H	3.1938856	-1.8808613	-3.0840779
H	-3.2940076	0.0015292	2.7679252
H	-2.7489069	0.9875079	-2.3183717
H	3.1561803	0.1130071	4.8142423
H	3.4182918	-0.8842468	-4.5219314
H	-3.4052818	-0.4730772	4.4629996
H	-3.0574357	0.6669070	-4.0266395
H	3.1108130	0.4476183	1.5794834
H	3.6164064	-0.4874332	-1.3596358
H	-2.9803683	-1.9295637	1.5873718
H	-3.0464666	-0.9818244	-1.2666729
H	3.5376920	1.7480184	2.7295961
H	4.3119599	0.6305191	-2.5677917
H	-3.6239136	-2.6631102	3.0865965
H	-3.8767551	-1.4808800	-2.7791976
C	1.4567806	-1.9262742	0.1928562
C	0.4391538	-3.0868305	0.2884666
H	2.1961655	-2.0405925	1.0019448
H	2.0731975	-2.0869172	-0.7122412
H	-0.1202556	-3.0764863	1.2404641
H	-0.3454695	-3.0323748	-0.4864393
H	0.8616610	-4.1000166	0.2196327

eB$^+$•THF : ethylene adduct **eB**$^+$ with one THF
76
Energy = -1856.375702899

Ca	0.1935109	0.0334447	-0.5677993
C	-0.1008339	0.1612754	-3.0654747
C	-0.6801208	1.3901875	-3.7989291
H	-0.6181710	-0.7439460	-3.4452610
H	0.9353773	0.0308791	-3.4297631
H	-0.6598335	1.3397232	-4.9031469
H	-1.7306961	1.5775523	-3.5260782
H	-0.1330007	2.3070134	-3.5291343
H	2.0740793	2.3665858	-2.5799975
C	2.8044793	1.6880985	-2.1390411
N	2.4213384	1.3895736	-0.7423669
H	3.8013203	2.1555309	-2.1786901
H	2.8042665	0.7789166	-2.7372917
C	2.2774834	2.6652799	-0.0041204
C	3.4452593	0.5399486	-0.0922553
C	0.9495440	3.3410985	-0.3161660
H	2.3476269	2.4442740	1.0651738
H	3.1070882	3.3497944	-0.2415923
C	3.6178504	-0.7942936	-0.8056313
H	3.1326391	0.3802792	0.9456078
H	4.4226738	1.0505625	-0.0622124
N	-0.1968329	2.5414072	0.1784757
H	0.8283959	3.4520084	-1.3972619
H	0.9364660	4.3542680	0.1154159
N	2.3384126	-1.5509664	-0.9190932
H	4.0088214	-0.6269622	-1.8109515
H	4.3729272	-1.3881772	-0.2725511
C	-0.3278979	2.6902703	1.6500895
C	-1.4363650	3.0048184	-0.4826201
C	2.2776558	-2.5641362	0.1610169
C	2.2664702	-2.2131655	-2.2419466
C	-0.9389194	1.4564086	2.2949024
H	0.6610920	2.8813533	2.0718386
H	-0.9449270	3.5685944	1.8914216
H	-2.2896420	2.4573477	-0.0809178
H	-1.5955040	4.0821882	-0.3208447
H	-1.3711793	2.8037741	-1.5537350

C	0.9446132	-3.2929287	0.2033909
H	3.0851730	-3.3042665	0.0336307
H	2.4606305	-2.0509989	1.1120319
H	2.4322965	-1.4689176	-3.0207958
H	3.0197694	-3.0115879	-2.3336381
H	1.2746075	-2.6389194	-2.4026464
N	-0.1044567	0.2466599	2.0982835
H	-1.9146610	1.2537004	1.8475950
H	-1.1043921	1.6470214	3.3679646
N	-0.1739342	-2.3871158	0.5661658
H	1.0089913	-4.1310658	0.9140183
H	0.7232298	-3.7274355	-0.7740112
C	-0.8871309	-0.9521251	2.4835290
C	1.1070359	0.3353885	2.9372307
C	-1.4437528	-2.9841253	0.0967324
C	-0.2156236	-2.2419491	2.0446096
H	-1.0478703	-0.9767547	3.5738108
H	-1.8684322	-0.8692384	2.0102499
H	1.6765080	1.2302063	2.6843121
H	0.8466555	0.3778021	4.0067765
H	1.7496708	-0.5304524	2.7697345
H	-1.4399537	-3.0302766	-0.9952076
H	-1.5770295	-4.0004149	0.4983713
H	-2.2803679	-2.3614542	0.4145701
H	-0.7438579	-3.0983956	2.4909420
H	0.8090466	-2.2767375	2.4211985
H	-2.3871422	-0.1539882	-2.5231713
C	-3.1433583	-0.4642065	-1.8057285
O	-2.6404547	-0.0833544	-0.4880322
C	-4.4876224	0.2328012	-1.9163288
H	-3.2604079	-1.5562542	-1.8428528
C	-3.7934432	0.1393588	0.3809323
C	-5.0459191	0.0328036	-0.4995097
H	-5.1247025	-0.2040767	-2.6894555
H	-4.3453912	1.2963431	-2.1362553
H	-3.7860149	-0.6026606	1.1870942
H	-3.6903515	1.1369443	0.8206413
H	-5.4914921	-0.9625842	-0.4087115
H	-5.7997655	0.7728030	-0.2209590

eB^{+} : ethylene adduct of L5-stabilized **2m**$^{+}$
63
Energy = -1623.773845882

Ca	0.4557305	0.1149964	-0.4838462
C	-0.7822726	0.1150924	-2.6146575
C	-2.3197235	0.1638465	-2.7297376
H	-0.4249147	-0.7706860	-3.1798443
H	-0.3651599	0.9744555	-3.1830783
H	-2.7183751	0.1845703	-3.7609448
H	-2.7832461	-0.7063000	-2.2420074
H	-2.7334797	1.0501442	-2.2265014
H	2.2845241	2.4291937	-2.5166062
C	2.9786944	1.6995496	-2.0981471
N	2.6123050	1.4187987	-0.6935232
H	4.0017325	2.0987098	-2.1715177
H	2.9061743	0.7923935	-2.6995088
C	2.4818482	2.6891181	0.0616567
C	3.6043106	0.5290820	-0.0437928
C	1.1742746	3.4034489	-0.2637634
H	2.5215959	2.4453549	1.1284898
H	3.3318394	3.3576311	-0.1445267
C	3.7340339	-0.8113000	-0.7616065
H	3.2786862	0.3777247	0.9923732
H	4.5985600	1.0032993	-0.0039611
N	-0.0026717	2.6010956	0.1505721
H	1.0944989	3.5705754	-1.3412353
H	1.1668661	4.3948131	0.2142559
N	2.4212904	-1.5019449	-0.9289134
H	4.1688367	-0.6581021	-1.7514876
H	4.4373381	-1.4447158	-0.2056612
C	-0.2516275	2.7503980	1.6054216
C	-1.1985630	3.0273372	-0.6094370
C	2.2599659	-2.5483604	0.1136812
C	2.3302172	-2.0950030	-2.2835070
C	-0.9840837	1.5453386	2.1798050
H	0.7086349	2.8816334	2.1097037
H	-0.8386316	3.6598905	1.8037849
H	-2.0660470	2.4478979	-0.2844421
H	-1.4116792	4.0958784	-0.4524686
H	-1.0379398	2.8352121	-1.6717540
C	0.8803180	-3.1964002	0.0880295
H	3.0257582	-3.3311285	-0.0100996
H	2.4359635	-2.0769747	1.0881462
H	2.5168024	-1.3171384	-3.0261752
H	3.0624156	-2.9050630	-2.4210112
H	1.3275122	-2.4860982	-2.4602466
N	-0.1985103	0.2953952	2.0275714
H	-1.9340738	1.4002855	1.6548396
H	-1.2227383	1.7319146	3.2389099
N	-0.1957179	-2.2258846	0.4155882
H	0.8672337	-4.0445643	0.7875205
H	0.6770919	-3.6062631	-0.9033929
C	-1.0552923	-0.8775272	2.3279263
C	0.9661709	0.3124449	2.9367018
C	-1.4535928	-2.6043967	-0.2669434
C	-0.4050870	-2.1798687	1.8838980
H	-1.2831152	-0.9310057	3.4042769
H	-2.0056270	-0.7342224	1.8037974
H	1.6025935	1.1713233	2.7189517
H	0.6486687	0.3702385	3.9891270
H	1.5635818	-0.5912952	2.8028171
H	-1.3022778	-2.5654224	-1.3474283
H	-1.7804916	-3.6135928	0.0258929
H	-2.2426970	-1.8940024	-0.0085190
H	-1.0279072	-3.0275243	2.2085860
H	0.5672764	-2.2988437	2.3688864

eC^{+} : L5-stabilized cation, deprotonated THF
68
Energy = -1776.506839807

Ca	-0.0691174	-0.3669152	-3.4692836
H	-0.4122844	1.8859840	-6.1726879
C	0.3249613	1.1229346	-6.4257338
N	1.2133793	0.8920169	-5.2665639
H	0.8933966	1.4580373	-7.3069977
H	-0.2209907	0.2105663	-6.6709006
C	1.7363145	2.1792981	-4.7523987
C	2.3396724	-0.0047372	-5.6192390
C	0.6756061	2.9569666	-3.9831120
H	2.5841631	1.9506772	-4.0981055
H	2.1263544	2.8022484	-5.5727176
C	1.8616467	-1.3505966	-6.1518110
H	2.9437846	-0.1417562	-4.7144420
H	2.9920913	0.4598883	-6.3772713
N	0.1958663	2.2087853	-2.7967044
H	-0.1907573	3.1477129	-4.6223441
H	1.0799826	3.9403264	-3.6962333
N	0.9205293	-2.0320996	-5.2194576
H	1.3523289	-1.2063615	-7.1069322
H	2.7365488	-1.9828849	-6.3557220
C	1.1683953	2.3246451	-1.6813565
C	-1.1062444	2.7571634	-2.3620152
C	1.6557634	-3.0296863	-4.4036210
C	-0.1680339	-2.6809257	-5.9851853
C	1.0900745	1.1366577	-0.7332230
H	2.1732795	2.4041160	-2.1015130
H	0.9907460	3.2519654	-1.1157744
H	-1.4419150	2.2333162	-1.4656389
H	-1.0299871	3.8326172	-2.1374534
H	-1.8476279	2.6043301	-3.1482060
C	0.7737691	-3.6960858	-3.3556568
H	2.0884218	-3.8092050	-5.0526290
H	2.4937116	-2.5135226	-3.9193166
H	-0.6335629	-1.9380719	-6.6345450
H	0.2107771	-3.5146483	-6.5978245
H	-0.9410300	-3.0451170	-5.3086937
N	1.4926400	-0.1309605	-1.3908132
H	0.0608616	1.0088098	-0.3817771
H	1.7177438	1.3320159	0.1513349
N	0.2765826	-2.7279419	-2.3462311
H	1.3372193	-4.5085624	-2.8736400
H	-0.0907569	-4.1577736	-3.8365004
C	1.1383238	-1.2744122	-0.5143947
C	2.9466614	-0.1277521	-1.6505573
C	-1.0239469	-3.1862125	-1.8078429
C	1.2593304	-2.6052141	-1.2413622
H	1.7759101	-1.2874611	0.3845159
H	0.1063027	-1.1243955	-0.1811472
H	3.2140802	0.7130726	-2.2919995
H	3.5187176	-0.0485224	-0.7128698
H	3.2422233	-1.0451861	-2.1620717
H	-1.7604770	-3.2024300	-2.6134110
H	-0.9368538	-4.1905505	-1.3647277
H	-1.3731251	-2.4867141	-1.0469664
H	1.1282734	-3.4276499	-0.5211126
H	2.2622537	-2.7137574	-1.6619263
C	-2.4270581	-0.6660237	-3.9640144
O	-2.1661666	-0.3260471	-2.5107805
C	-3.4453809	0.4102053	-4.3216115
H	-2.9607070	-1.6378934	-3.9363604
C	-3.4145729	0.0667320	-1.8969168
C	-4.2955894	0.6310307	-3.0339646
H	-4.0687757	0.1095397	-5.1713287
H	-2.9565436	1.3550492	-4.6014370
H	-3.8693796	-0.8218570	-1.4384515
H	-3.1956413	0.7962360	-1.1097825
H	-5.2383395	0.0785382	-3.0917779
H	-4.5369032	1.6869919	-2.8702088

eTS10$^+$: ethylene addition to **1m**$^+$

52

Energy = -1450.343967361

Ca	0.1012070	-0.9407521	0.0059721
N	0.1943202	0.5485700	-2.0414020
N	-2.1998298	0.0371656	-0.3115643
N	-0.4378381	0.2313185	2.1429901
N	1.9552839	0.7570835	0.4280315
H	-0.6172114	-2.7656274	0.7607321
C	-0.9845307	1.4524138	-1.9746441
C	1.4582623	1.3290875	-1.9654052
C	0.1703403	-0.2203363	-3.3068046
C	-2.2703387	0.7061338	-1.6370713
C	-2.3903478	1.0246565	0.7834004
C	-3.2382931	-1.0170062	-0.2338913
C	-1.8986677	0.5086871	2.1322780
C	0.3456973	1.4895615	2.2052679
C	-0.1134890	-0.6412975	3.2951907
C	1.8048405	1.2799773	1.8136570
C	1.7490456	1.8484694	-0.5611663
C	3.3129932	0.1860535	0.2712403
H	-1.1124066	1.9821574	-2.9307887
H	-0.7871766	2.2136144	-1.2156298
H	1.4325263	2.1797045	-2.6634155
H	2.2651026	0.6688847	-2.2962950
H	-0.7258305	-0.8436161	-3.3507205
H	1.0485148	-0.8678377	-3.3508599
H	0.1752247	0.4482320	-4.1804512
H	-3.1177063	1.4068072	-1.6791998
H	-2.4626183	-0.0697301	-2.3833667
H	-3.4538257	1.2947991	0.8698212
H	-1.8549361	1.9390727	0.5135015
H	-3.1727196	-1.5302605	0.7264563
H	-3.0644851	-1.7510933	-1.0244706
H	-4.2458196	-0.5905127	-0.3528569
H	-2.1629937	1.2364448	2.9144867
H	-2.4030958	-0.4291933	2.3798505
H	0.3042901	1.9205431	3.2173141
H	-0.1215993	2.2150903	1.5340892
H	0.9554904	-0.8669642	3.3045508
H	-0.6628611	-1.5792191	3.1905341

H	-0.3773354	-0.1584529	4.2480079
H	2.3523499	2.2267780	1.9334650
H	2.2732940	0.5551272	2.4851095
H	2.6370384	2.4975360	-0.5981485
H	0.9209538	2.4701577	-0.2118702
H	3.4430962	-0.1952780	-0.7433998
H	3.4409465	-0.6407389	0.9742603
H	4.0872020	0.9429166	0.4660458
C	0.6952348	-3.6793665	-0.1410054
C	1.5655245	-2.9191420	-0.8953994
H	0.9936381	-4.0649261	0.8274654
H	-0.1090874	-4.2309396	-0.6145697
H	2.5207122	-2.5915936	-0.4920490
H	1.3972962	-2.7641126	-1.9588100

eTS1a^{2+} : second ethylene addition to **1**$^{2+}$

104

Energy = -2900.738747157

Ca	0.0923098	-0.2089386	1.8459294
Ca	-0.2234610	-0.2952330	-1.7320950
H	-1.0081685	0.7029624	0.1178326
N	1.6395882	-1.4155261	3.5980499
N	0.3467972	-1.9339165	-3.6655526
N	-1.0573907	1.9441078	2.8738891
N	-0.9704358	2.0268112	-2.6823983
N	1.8891659	1.4014088	2.5002164
N	1.7252679	0.7237317	-3.1296529
N	-1.3389673	-0.9043736	3.8824030
N	-2.3887351	-0.6162790	-3.2589623
C	-0.0012690	2.9899249	2.9048429
C	0.2337998	2.7213197	-3.2094432
C	0.7597281	-2.0822374	4.5941047
C	-0.9230244	-2.3346768	-4.3310679
C	1.3515287	2.4752108	3.3734399
C	1.1421773	1.7854941	-3.9913008
C	-0.4265735	-1.2301212	5.0126500
C	-1.8677385	-1.1605275	-4.5417689
C	2.5874146	-2.4365887	3.0908256
C	1.0384254	-3.1640421	-3.2099665
C	-2.2218182	2.5201880	2.1614708
C	-1.5931738	2.8943117	-1.6580345
C	2.4164340	-0.3164504	4.2315692
C	1.2197706	-1.2205687	-4.6376533
C	-1.4746818	1.5691107	4.2493372
C	-1.9419664	1.8077149	-3.7860854
C	2.9716976	0.6592005	3.2014458
C	2.2890718	-0.3682895	-3.9691313
C	-2.2123609	0.2372379	4.2713831
C	-2.9726069	0.7362348	-3.4693695
C	2.4145947	1.9824390	1.2458346
C	2.8271424	1.3176796	-2.3376030
C	-2.1967672	-2.0798881	3.5963820
C	-3.4902158	-1.4963399	-2.7967215
H	-0.3063708	3.8268369	3.5521157
H	-0.0576230	3.5677332	-3.8495558
H	1.3339576	-2.3578903	5.4923348
H	-0.7131970	-2.8106153	-5.3009032
H	0.0785313	3.3884450	1.8889016
H	0.7689341	3.1410456	-2.3533099
H	0.4143277	-3.0159067	4.1400883
H	-1.3952039	-3.0928953	-3.6999401
H	2.0339039	-3.2229046	2.5733257
H	0.4138549	-3.6835997	-2.4797578
H	-1.9626445	2.6601484	1.1113804
H	-0.8890581	3.0346378	-0.8355228
H	1.7633751	0.2135780	4.9285118
H	0.5880152	-0.5962003	-5.2724085
H	-0.5846797	1.5158464	4.8804628
H	-1.3849850	1.5397255	-4.6859759
H	1.2737947	2.0805635	4.3890083
H	0.5800996	1.3088994	-4.7971702
H	-0.0775898	-0.2932190	5.4519978
H	-1.3499899	-0.3606999	-5.0751994
H	3.1546846	-2.8832723	3.9208643
H	1.2340751	-3.8381297	-4.0569949
H	-2.5109109	3.4854774	2.6024530
H	-1.8611403	3.8745499	-2.0806045
H	3.2443325	-0.7294964	4.8269554
H	1.7099810	-1.9462847	-5.3035834
H	-2.1193840	2.3493462	4.6812036
H	-2.4683763	2.7467653	-4.0145387
H	2.0578469	3.3180007	3.4156454
H	1.9446727	2.3686554	-4.4677537
H	-0.9841647	-1.7574651	5.8011082
H	-2.7034142	-1.4765681	-5.1842654
H	3.2875378	-1.9841038	2.3893947
H	1.9885617	-2.9065681	-2.7389525
H	-3.0773597	1.8462443	2.2253334
H	-2.4970413	2.4233954	-1.2670908
H	1.6158522	2.5150450	0.7229323
H	2.4466905	2.1372693	-1.7262082
H	-1.5768848	-2.9352670	3.3178871
H	-3.1358717	-2.5235318	-2.6977433
H	3.5450551	0.1198439	2.4421650
H	2.9148451	-0.9868795	-3.3197967
H	-3.0525745	0.2590490	3.5706839
H	-3.5181519	0.9946325	-2.5562410
H	3.6640638	1.3589456	3.6919024
H	2.9478839	0.0479968	-4.7469354
H	-2.6367749	0.0695987	5.2726534
H	-3.7132521	0.7081399	-4.2839986
H	2.7732147	1.1750247	0.6051627
H	3.2573181	0.5600049	-1.6832827
H	-2.8678105	-1.8493929	2.7658077
H	-3.8514915	-1.1586478	-1.8241034
H	3.2380545	2.6853486	1.4394956
H	3.6148202	1.7117861	-2.9968131
H	-2.7953445	-2.3540015	4.4773327
H	-4.3218745	-1.4811636	-3.5168234
C	1.2579853	-1.5986690	-0.0147729

C	2.7838658	-1.6464312	-0.2394833
H	0.8167717	-2.3206049	-0.7285935
H	1.0653367	-2.1570796	0.9261547
H	3.0389671	-1.4110896	-1.2791071
H	3.3275805	-0.9152186	0.3742197
H	3.2352703	-2.6287284	-0.0321418
C	-2.1668478	-0.2653302	0.2663234
C	-1.9000942	-1.6285354	0.1025189
H	-2.7812514	0.2452080	-0.4760871
H	-2.4204625	0.0909540	1.2670315
H	-1.5435502	-2.2526617	0.9160451
H	-1.9632608	-2.1222728	-0.8612392

eTS1^{+} : ethylene addition to **1m**$^{+}$•THF
65
Energy = -1682.957632556

Ca	0.0450190	-0.4206145	-0.2472965
N	0.3009199	1.7102936	1.3352636
N	-0.6782436	-1.0342084	2.1742097
N	-1.3692601	1.5241049	-1.1680072
H	0.0135357	-1.1369039	-2.2554285
C	0.1781596	1.2498485	2.7409156
C	1.5870669	2.4237137	1.1826883
C	-0.8111025	2.6349740	1.0006320
C	-0.9290097	0.2214618	2.9258714
C	-1.9322890	-1.8269973	2.0711588
C	0.3407376	-1.8339115	2.8912873
C	-2.7867046	1.1865736	-0.8817385
C	-1.0001692	2.7972648	-0.5010277
C	-1.1750540	1.6733333	-2.6283920
H	-0.0059779	2.1004098	3.4164833
H	1.1424179	0.8208315	3.0284285
H	2.4077162	1.7494438	1.4315025
H	1.6324368	3.3021125	1.8452398
H	1.7130626	2.7554858	0.1510099
H	-1.7278047	2.2473496	1.4508580
H	-0.6325966	3.6239657	1.4517102
H	-1.8821529	0.6371457	2.5912314
H	-1.0396938	0.0028119	3.9996418
C	-2.9250647	-1.2373685	1.0737685
H	-1.6488618	-2.8365206	1.7609925
H	-2.4173503	-1.9154304	3.0561424
H	1.2936235	-1.2992094	2.9111879
H	0.4860117	-2.7830149	2.3748450
H	0.0309602	-2.0325536	3.9288087
C	-3.1203526	-0.2705034	-1.1736171
H	-2.9870457	1.4114803	0.1689812
H	-3.4551060	1.8309919	-1.4747731
H	-0.0708623	3.1463827	-0.9603104
H	-1.7600364	3.5726704	-0.6882347
H	-1.3773950	0.7237048	-3.1233218
H	-0.1332982	1.9369053	-2.8240594
H	-1.8268648	2.4634536	-3.0343572
N	-2.3842372	-1.2236567	-0.3055761
H	-3.1778483	-0.2122848	1.3560015
H	-3.8613709	-1.8157668	1.1183005
H	-4.2083415	-0.4149834	-1.0751562
H	-2.8493317	-0.5138425	-2.2042854
C	-2.4926128	-2.5719944	-0.9037622
H	-2.0137461	-2.5540402	-1.8843660
H	-3.5465719	-2.8736238	-1.0076760
H	-1.9818425	-3.3034734	-0.2754706
C	0.9398445	-2.7192440	-1.6725494
C	1.0093455	-2.9291977	-0.3189748
H	0.2090621	-3.2358144	-2.2808449
H	1.7883752	-2.3246214	-2.2192753
H	0.2527797	-3.5092972	0.2017157
H	1.8699438	-2.6119297	0.2651766
H	2.4336891	1.7806477	-2.0464649
C	2.5918836	0.6980219	-2.1219046
O	2.3441571	0.1185781	-0.7962240
C	4.0426545	0.3630297	-2.4466832
H	1.8562591	0.2576448	-2.8007087
C	3.6070373	-0.3387385	-0.2225102
C	4.6896747	0.3360169	-1.0532169
H	4.4928116	1.1006351	-3.1148881
H	4.1119662	-0.6228469	-2.9178809
H	3.6541082	-1.4302165	-0.3102815
H	3.6134093	-0.0602284	0.8346881
H	4.8702926	1.3543949	-0.6933339
H	5.6312321	-0.2172993	-1.0268651

eTS1^{2+} : ethylene addition to cation dimer **1**$^{2+}$
98
Energy = -2822.085589660

Ca	0.1012797	-0.0451337	1.7639793
Ca	0.0048553	-0.0118235	-1.7661852
H	-0.7479074	0.9028020	0.0301585
N	2.0911426	-0.3823236	3.4838470
N	1.8994564	-0.3169587	-3.5967413
N	-2.1129155	0.4007358	2.8979291
N	-2.2673400	0.4476240	-2.7721140
N	0.3700852	2.0932515	3.0827872
N	0.1994987	2.1468602	-3.0653032
N	-0.4409810	-2.0655285	3.2205216
N	-0.6114857	-2.0111197	-3.2225669
C	-2.1063922	1.8167840	3.3484819
C	-2.2877787	1.8705099	-3.2000284
C	1.8954765	-1.7313296	4.0827274
C	1.6749831	-1.6580726	-4.2034191
C	-0.7916235	2.2242550	4.0011428
C	-1.0108342	2.2909700	-3.9163200
C	0.4458015	-2.0580104	4.4123882
C	0.2105332	-1.9844525	-4.4598811
C	3.5025225	-0.3018080	3.0355275
C	3.3326131	-0.2388697	-3.2234147
C	-3.2373621	0.2070083	1.9574866
C	-3.3394061	0.2354145	-1.7760954
C	1.8585600	0.6818729	4.4984631
C	1.6100314	0.7608629	-4.5817632

C	-2.2399010	-0.5349154	4.0414861
C	-2.4530420	-0.4702759	-3.9221365
C	1.6357313	2.0471361	3.8588356
C	1.4203723	2.1161058	-3.9108536
C	-1.8615560	-1.9595453	3.6505174
C	-2.0529796	-1.9002596	-3.5747027
C	0.4003069	3.2286577	2.1323890
C	0.2809621	3.2657146	-2.0984933
C	-0.2526617	-3.3217453	2.4583725
C	-0.3826839	-3.2792302	-2.4916274
H	-2.9317147	2.0091566	4.0510887
H	-3.1499963	2.0713863	-3.8542367
H	2.4950385	-1.8318901	5.0010298
H	2.2250664	-1.7441715	-5.1535889
H	-2.2877417	2.4309059	2.4621166
H	-2.4223780	2.4705965	-2.2958881
H	2.2919815	-2.4574625	3.3662375
H	2.1106482	-2.3931540	-3.5193363
H	3.6819260	-1.0306885	2.2431487
H	3.5549834	-0.9773907	-2.4512469
H	-3.1210925	0.9127451	1.1354288
H	-3.1816117	0.9289230	-0.9504828
H	0.9846669	0.4076858	5.0940639
H	0.7051690	0.4931341	-5.1323503
H	-1.5909533	-0.1824453	4.8474926
H	-1.8484004	-0.1034955	-4.7557304
H	-0.6020241	1.6042138	4.8808214
H	-0.8687096	1.6856704	-4.8149400
H	0.0489597	-1.3313710	5.1250589
H	-0.2254061	-1.2494425	-5.1404694
H	4.1857204	-0.5078567	3.8729004
H	3.9705684	-0.4322095	-4.0986037
H	-4.2058938	0.3696360	2.4541948
H	-4.3335796	0.4020115	-2.2177575
H	2.7100929	0.7351065	5.1933240
H	2.4220559	0.8261899	-5.3214924
H	-3.2683340	-0.5277843	4.4341851
H	-3.5008473	-0.4592362	-4.2597513
H	-0.8768217	3.2616724	4.3589648
H	-1.1162827	3.3339278	-4.2519064
H	0.4128909	-3.0375808	4.9133582
H	0.1531217	-2.9571418	-4.9718816
H	3.7202975	0.6941497	2.6475897
H	3.5691581	0.7524974	-2.8345732
H	-3.2187568	-0.8052763	1.5472028
H	-3.2958070	-0.7828473	-1.3828594
H	-0.5111010	3.2307598	1.5318171
H	-0.5950170	3.2544332	-1.4474583
H	0.7924425	-3.4215201	2.1574501
H	0.6768198	-3.3821950	-2.2475136
H	2.4522838	2.2786135	3.1688736
H	2.2734479	2.3398407	-3.2639361
H	-2.4811613	-2.2973477	2.8156415
H	-2.6271869	-2.2526671	-2.7138371
H	1.6455418	2.8234049	4.6386703
H	1.3851960	2.9037196	-4.6783675
H	-2.0635343	-2.6369180	4.4936022
H	-2.2989715	-2.5642236	-4.4168009
H	1.2512424	3.1070660	1.4574763
H	1.1688835	3.1348899	-1.4748832
H	-0.8679647	-3.2909617	1.5563989
H	-0.9499471	-3.2638373	-1.5583418
H	0.4918778	4.1894365	2.6607301
H	0.3407324	4.2358927	-2.6140561
H	-0.5295481	-4.1986866	3.0622787
H	-0.6903545	-4.1466494	-3.0944458
C	2.2257746	0.2961019	-0.0570348
C	1.9185596	-1.0584150	-0.0629142
H	2.4353014	0.8387234	0.8591097
H	2.3854262	0.8575730	-0.9717234
H	2.0989459	-1.6673020	0.8214750
H	0.2917011	-1.4235982	-0.0213105
H	2.0493416	-1.6470350	-0.9694903

eTS20$^+$: hydrogenolysis of adduct L4CaEt$^+$

54

Energy = -1451.548898942

C	3.2615510	-1.7895965	1.0677858
C	2.8900035	-1.6061647	-0.4087700
Ca	0.8918230	-0.1133790	-0.2093982
N	0.0446343	1.0548654	1.8339962
C	1.1591081	1.6004086	2.6446842
C	-0.7365732	0.0861165	2.6496548
C	-1.5997168	-0.8329935	1.7912229
N	-0.7867667	-1.6821229	0.8802644
C	-0.1872644	-2.8048632	1.6396857
C	-1.6292817	-2.2477718	-0.2087570
C	-2.0050254	-1.2207703	-1.2704165
N	-0.8210653	-0.6437433	-1.9600288
C	-0.2695936	-1.6124257	-2.9361890
C	-1.1944969	0.6154701	-2.6602230
C	-1.3094158	1.7937817	-1.6985677
N	-0.0215589	2.0823667	-1.0166023
C	0.8993824	2.7790728	-1.9455798
C	-0.8313216	2.1595527	1.3584811
C	-0.2306606	2.9301928	0.1873406
H	-2.6763775	-1.6950079	-2.0020726
H	-2.5647343	-0.3986228	-0.8171120
H	-2.5494432	-2.6886525	0.2035689
H	-1.0607530	-3.0653063	-0.6611957
H	-2.2212201	-1.4617941	2.4463301
H	-2.2855142	-0.2389245	1.1817052
H	-1.3756395	0.6123925	3.3741853
H	-0.0192386	-0.5035981	3.2270142
H	-1.0339349	2.8615679	2.1812783
H	-1.7934004	1.7283236	1.0697766
H	-0.8804984	3.7868771	-0.0474834
H	0.7462936	3.3346158	0.4658296
H	-1.6595203	2.6791835	-2.2505097
H	-2.0615265	1.5800018	-0.9343723

H	-2.1426426	0.4917753	-3.2036040
H	-0.4219928	0.8139644	-3.4080271
H	0.0098027	-2.5376368	-2.4276353
H	0.6241534	-1.1874789	-3.4004410
H	-1.0015943	-1.8499329	-3.7215073
H	0.4090958	-2.4217018	2.4692700
H	0.4642755	-3.3812326	0.9789477
H	-0.9666412	-3.4672911	2.0438211
H	1.7515132	2.2923116	2.0431033
H	1.8066921	0.7803973	2.9649155
H	0.7831463	2.1280901	3.5336294
H	1.0906823	2.1547612	-2.8211560
H	1.8483393	2.9564002	-1.4363310
H	0.4727368	3.7357945	-2.2822623
H	3.7449730	-1.8255935	-1.0574795
H	2.0966021	-2.3249308	-0.7043968
H	4.1375672	-1.1786065	1.3172485
H	2.4591109	-1.4520812	1.7496011
H	2.9453590	-0.0301150	-0.4893959
H	2.8673463	0.9123802	-0.3413510
H	3.4840615	-2.8240312	1.3628986

eTS2^{+} : hydrogenolysis of **eA**$^{+}$ with H_2
67
Energy = -1684.157379838

Ca	0.4377507	-0.0346986	-0.1110306
N	1.3406886	0.5650240	2.1980165
N	-2.2199652	0.1196598	-0.1135824
N	-0.2924015	2.3771643	0.3704521
C	1.0260807	-0.5175238	3.1653521
C	2.8107895	0.7448579	2.1476608
C	0.6975848	1.8380443	2.6160537
C	-2.5783627	1.5602020	-0.1696220
C	-2.8261786	-0.5625277	-1.2781947
C	-2.7538788	-0.4784974	1.1333598
C	-1.7218761	2.4079475	0.7619880
C	0.5835322	2.8430172	1.4769555
C	-0.0844782	3.2737598	-0.7895317
C	-0.4025986	-1.0247707	3.0312990
H	1.1935551	-0.1752192	4.1985972
H	1.7333103	-1.3314397	2.9807530
H	3.2741064	-0.1830102	1.8070256
H	3.2073729	1.0071555	3.1403374
H	3.0582171	1.5343035	1.4366959
H	-0.2937954	1.6080450	3.0141312
H	1.2674194	2.2960608	3.4392676
H	-3.6419426	1.7051092	0.0772922
H	-2.4440634	1.8876811	-1.2042285
H	-2.4363177	-0.1171954	-2.1941162
H	-3.9232544	-0.4710413	-1.2637602
H	-2.5675157	-1.6238275	-1.2667744
C	-2.1054583	-1.8100351	1.4723425
H	-2.6014152	0.2321267	1.9485994
H	-3.8421301	-0.6265176	1.0473523
H	-1.8085319	2.0420318	1.7879084
H	-2.1004437	3.4420076	0.7622001
H	1.5691894	3.0196664	1.0370143
H	0.2199108	3.8020478	1.8795269
H	-0.7247961	2.9679510	-1.6193607
H	0.9588431	3.2000450	-1.1036441
H	-0.3264717	4.3155512	-0.5281974
N	-0.6449073	-1.7115519	1.7341606
H	-1.1000281	-0.1888836	3.1155362
H	-0.6263655	-1.7066216	3.8664537
H	-2.2356496	-2.5149664	0.6455395
H	-2.6199126	-2.2441960	2.3447156
C	-0.1343533	-3.0993050	1.8359048
H	0.9240116	-3.0960707	2.0955331
H	-0.2582377	-3.6067359	0.8768990
H	-0.6859211	-3.6568073	2.6084598
H	-1.0932676	-1.7032906	-3.4556525
C	-0.0469329	-1.5454514	-3.1716049
C	0.8620544	-1.4594611	-4.3889729
H	0.2526254	-2.3102176	-2.4506067
O	0.0550216	-0.2406404	-2.5131024
C	0.7082538	0.0139004	-4.7943134
H	0.5585344	-2.1542453	-5.1755359
H	1.8976520	-1.6752181	-4.1086531
C	0.6606451	0.7219193	-3.4449970
H	-0.2282790	0.1633822	-5.3412829
H	1.5335681	0.3753084	-5.4120128
H	1.6573139	0.9660925	-3.0666263
H	0.0357830	1.6174952	-3.4419564
H	2.4812727	-2.3792235	0.7723053
C	2.3327351	-2.3663655	-0.3148834
C	2.7721681	-1.0272614	-0.9178588
H	1.2630668	-2.5868817	-0.4861801
H	3.8098194	-0.8247823	-0.6145337
H	2.7778144	-1.0935604	-2.0161542
H	2.3760984	0.4865830	-0.7462125
H	2.0998683	1.3848070	-0.6835999
H	2.8642290	-3.2465572	-0.7116165

eTS2^{2+} : hydrogenolysis of **eA**$^{2+}$ with H_2
100
Energy = -2823.290577514

Ca	-0.0700134	-0.2927173	1.8061569
Ca	0.0675927	-0.1291389	-1.7829178
H	-1.0134890	0.3900237	0.0026057
N	1.9490129	-0.8054148	3.2581869
N	0.9302736	-1.8101236	-3.5449749
N	-1.6654149	1.3398767	2.9352483
N	-1.1773409	1.8501328	-2.8078297
N	1.1983865	1.8962524	2.1482300
N	1.6882727	1.1231194	-3.3340920
N	-0.9418007	-1.3166240	4.1401779
N	-1.9396871	-1.0837881	-2.9821031
C	-1.0667708	2.6933233	2.8058620
C	-0.1948344	2.7694677	-3.4383923
C	1.5236239	-1.6405842	4.4140040

C	-0.2373290	-2.6122251	-4.0002791
C	0.4228645	2.6943791	3.1312060
C	0.8913080	2.0285043	-4.2054513
C	0.2271059	-1.1480631	5.0443986
C	-1.4905627	-1.7722130	-4.2200133
C	2.9670138	-1.5442548	2.4748609
C	1.9863225	-2.7355302	-3.0705223
C	-2.9607935	1.3062155	2.2176244
C	-1.9670567	2.6089448	-1.8122463
C	2.5194674	0.4808013	3.7412940
C	1.4685783	-0.9873880	-4.6587910
C	-1.8694496	1.0113958	4.3669952
C	-2.0880038	1.2713427	-3.8294928
C	2.5501985	1.5539852	2.6619077
C	2.3977592	0.1141476	-4.1664735
C	-2.0803044	-0.4757525	4.5996805
C	-2.8415577	0.0528438	-3.3105384
C	1.3563231	2.6775250	0.9043552
C	2.6926475	1.9136577	-2.5859556
C	-1.3958800	-2.7267578	4.2089652
C	-2.6698815	-2.0289843	-2.1059876
H	-1.5859877	3.4120425	3.4577311
H	-0.6975016	3.4762035	-4.1163621
H	2.3136304	-1.6690073	5.1794221
H	-0.0003568	-3.1523443	-4.9297373
H	-1.2267356	3.0203319	1.7748583
H	0.2509004	3.3634442	-2.6349169
H	1.3990836	-2.6640969	4.0478744
H	-0.4233208	-3.3678033	-3.2308616
H	2.5325109	-2.4778480	2.1098852
H	1.5794237	-3.3746755	-2.2861477
H	-2.7751016	1.4854612	1.1569832
H	-1.2926170	3.0174330	-1.0574205
H	1.9220646	0.8216776	4.5901584
H	0.6274557	-0.5501272	-5.2023552
H	-0.9978958	1.3605856	4.9256384
H	-1.4925809	0.9968927	-4.7042748
H	0.5894796	2.2746878	4.1263653
H	0.4442420	1.4286935	-5.0013273
H	0.3166460	-0.0893632	5.2989774
H	-1.3060393	-1.0123908	-4.9834643
H	3.8491855	-1.7770258	3.0891793
H	2.3566916	-3.3645470	-3.8933680
H	-3.6562853	2.0596950	2.6165283
H	-2.5175085	3.4331359	-2.2908374
H	3.5410502	0.3228733	4.1185742
H	2.0105038	-1.6248896	-5.3738224
H	-2.7374919	1.5594172	4.7648292
H	-2.8137466	2.0278109	-4.1651055
H	0.7907650	3.7312907	3.1582881
H	1.5510313	2.7602394	-4.6953242
H	0.0563529	-1.6851959	5.9897906
H	-2.2901540	-2.4205075	-4.6086564
H	3.2785130	-0.9460318	1.6162341
H	2.8246877	-2.1701998	-2.6572654
H	-3.4184586	0.3196464	2.3234792
H	-2.6697920	1.9443153	-1.3095828
H	0.3767593	2.8955769	0.4755401
H	2.1960411	2.7037582	-2.0196003
H	-0.5724009	-3.4043091	3.9780762
H	-2.0532284	-2.9074095	-1.9068267
H	3.1320811	1.2039560	1.8038754
H	3.1973280	-0.3109913	-3.5535630
H	-2.9676522	-0.8199565	4.0601922
H	-3.3821854	0.3082144	-2.3952864
H	3.0576025	2.4473992	3.0575201
H	2.8789453	0.5978847	-5.0295586
H	-2.2733178	-0.6448973	5.6706588
H	-3.5929166	-0.2530158	-4.0537793
H	1.9225000	2.0771386	0.1908241
H	3.2238547	1.2592990	-1.8897885
H	-2.1980524	-2.8882425	3.4859090
H	-2.8911271	-1.5326670	-1.1574672
H	1.8848500	3.6242728	1.0933327
H	3.4191664	2.3801853	-3.2673678
H	-1.7676128	-2.9639080	5.2169680
H	-3.6088741	-2.3590667	-2.5736610
C	0.3586298	-2.3145500	-0.0689053
C	-0.6059152	-2.8878498	0.9727148
H	1.2871136	-2.9013998	-0.0561398
H	-0.0765343	-2.4881240	-1.0673960
H	-0.1484762	-2.8930401	1.9725766
H	-1.5468872	-2.3139098	1.0374224
H	1.1957755	-0.9035147	-0.0009287
H	1.6405799	-0.0882486	-0.0301560
H	-0.9070598	-3.9290654	0.7924551

eTS3$^+$: ethylene addition of L5-stabilized **2m$^+$**
63
Energy = -1623.740427345

Ca	0.5817925	0.0209227	0.0310451
H	2.1210824	-0.1190746	1.4917836
N	0.1949392	2.3280926	1.2258569
N	-0.9470626	-0.3865638	2.0286572
N	0.4296548	-2.5764867	0.4525322
N	-0.6673604	-1.1371920	-1.9068195
N	-0.4367479	1.8048764	-1.6774082
C	1.4760967	2.7977232	1.7990641
C	-0.3478416	3.3462591	0.2936635
C	-0.7821563	2.0838180	2.3184152
C	-0.6003489	0.7176637	2.9607440
C	-0.4544119	-1.6681646	2.5983494
C	-2.4098792	-0.4424349	1.8326444
C	-0.5044402	-2.8020366	1.5859795
C	-0.0633109	-3.2624369	-0.7644956
C	1.7701597	-3.0872860	0.8114243
C	-1.1622456	-2.4574379	-1.4449025
C	0.1359305	-1.3079041	-3.1366166
C	-1.8170563	-0.2330246	-2.1488215
C	-1.2086739	2.7115375	-0.7879633

C	0.5743671	2.5713740	-2.4403259
C	-1.3767822	1.1430800	-2.6273785
H	2.1954374	2.9547322	0.9910753
H	1.3436674	3.7423577	2.3492785
H	1.8751514	2.0307155	2.4646709
H	-0.9404489	4.0990036	0.8338747
H	0.4924530	3.8774394	-0.1585394
H	-0.6885838	2.8653301	3.0881283
H	-1.7882683	2.1739632	1.9000982
H	-1.2139444	0.6563628	3.8740100
H	0.4483191	0.5775791	3.2403927
H	0.5819083	-1.4961957	2.9059880
H	-1.0412724	-1.9438635	3.4895388
H	-2.6697907	-1.2537645	1.1508008
H	-2.9326610	-0.6072275	2.7874890
H	-2.7694948	0.4911182	1.3959259
H	-1.5157350	-2.9082980	1.1861415
H	-0.2653608	-3.7504197	2.0904333
H	0.7870657	-3.3878824	-1.4403676
H	-0.4364442	-4.2718784	-0.5317904
H	2.1360236	-2.5486243	1.6873905
H	1.7413106	-4.1674422	1.0243137
H	2.4581544	-2.9076177	-0.0178757
H	-1.9826181	-2.2744721	-0.7430533
H	-1.5830649	-3.0328373	-2.2841730
H	0.8925227	-2.0761180	-2.9739912
H	-0.4964240	-1.6093378	-3.9861032
H	0.6531848	-0.3807015	-3.3874959
H	-2.3775607	-0.1525159	-1.2093203
H	-2.5034327	-0.6611821	-2.8978504
H	-1.6871491	3.5062547	-1.3831264
H	-2.0135122	2.1232622	-0.3310472
H	1.0609723	1.9045511	-3.1546763
H	0.1189175	3.4093682	-2.9901273
H	1.3399166	2.9673642	-1.7708224
H	-0.8798598	1.0538929	-3.5953830
H	-2.2664177	1.7667764	-2.7889260
C	2.9480531	-0.0253969	-1.1903079
C	3.4265107	-0.0169853	0.0963910
H	2.8241851	-0.9534440	-1.7431952
H	2.7724162	0.8989661	-1.7346855
H	3.7476427	0.9070015	0.5637589
H	3.8235776	-0.9160144	0.5506733

eTS3^{2+} : ethylene addition of L5-stabilized **2^{2+}**
120
Energy = -3168.840012613

C	3.7114556	-2.3870229	1.9104004
H	5.8308272	-1.9849567	1.9633763
N	2.5710992	-2.2404090	0.9685830
H	4.0001888	-3.4469898	1.9811073
H	3.3600183	-2.0860731	2.8997961
C	4.9214608	-1.5453100	1.5156966
Ca	1.8627019	0.1957847	0.2079278
C	1.3388317	-2.7142101	1.6382715
C	2.8252901	-3.0806276	-0.2327474
N	4.7744292	-0.1380096	1.9186684
H	5.0588778	-1.5787019	0.4298107
Ca	-2.1586729	-0.0217495	0.0096422
N	2.5848049	-1.2513467	-1.9032552
N	1.5827127	1.5805699	-2.0073727
N	4.0133916	1.9117594	-0.2022456
H	-0.1362905	-0.7841061	-0.3261803
H	0.0255016	1.3945889	0.8455512
H	1.1670397	-2.1056866	2.5296173
H	1.4253913	-3.7702708	1.9378979
H	0.4895814	-2.5893773	0.9602280
C	2.0799512	-2.5741043	-1.4548647
H	2.5318073	-4.1216138	-0.0292533
H	3.9006815	-3.0921989	-0.4222358
C	4.9515509	-0.0033264	3.3736638
C	5.7449040	0.7266996	1.2264231
N	-2.4862286	-0.0076990	2.5649642
N	-3.9519704	-1.8963375	0.8367588
N	-2.7725073	-1.8884440	-1.9179284
N	-3.6951806	0.9874734	-1.8668638
N	-3.1584929	2.3564486	0.6932298
C	1.6566778	-0.7099710	-2.9240749
C	3.9310174	-1.4027698	-2.4893825
C	1.9100366	0.7602160	-3.2042604
C	2.4335524	2.7906106	-1.9356977
C	0.1677050	1.9753318	-2.0860716
C	3.8688988	2.4383816	-1.5844628
C	4.0735994	3.0696504	0.7180249
C	5.2981185	1.1572028	-0.1620742
H	2.1569564	-3.3139605	-2.2682317
H	1.0222591	-2.4359037	-1.2070348
H	4.8115483	1.0420571	3.6599370
H	5.9552418	-0.3299736	3.6956180
H	4.2066456	-0.5990459	3.9048545
H	5.9063991	1.6110479	1.8477462
H	6.7280491	0.2343590	1.1289501
C	-1.1878386	-0.4546799	3.1141013
C	-2.7641703	1.3913548	2.9737256
C	-3.5585744	-0.9006062	3.0684333
C	-3.6162779	-2.1788275	2.2540555
C	-3.6813844	-3.1154263	0.0353994
C	-5.3900335	-1.5576241	0.7542931
C	-3.8059304	-2.8502648	-1.4528372
C	-3.2616920	-1.1353756	-3.0968295
C	-1.5721834	-2.6600378	-2.2958646
C	-4.2918598	-0.0902913	-2.6994646
C	-3.0625285	1.9756539	-2.7699523
C	-4.7723913	1.6387082	-1.0759629
C	-3.7457132	2.0711689	2.0304862
C	-2.1741515	3.4585663	0.8052956
C	-4.2492058	2.7865448	-0.2304771
H	0.6513394	-0.8153765	-2.5055611
H	1.7140343	-1.2932836	-3.8572949
H	3.9162850	-2.1142940	-3.3298431

H	4.6349008	-1.7655289	-1.7395133
H	4.2953757	-0.4423426	-2.8553195
H	2.9515185	0.9257844	-3.4820720
H	1.3035690	1.0846044	-4.0623603
H	2.0027447	3.4501406	-1.1750737
H	2.4131787	3.3424361	-2.8888915
H	-0.4374869	1.0667351	-2.1639468
H	-0.0275064	2.6080564	-2.9660138
H	-0.1141168	2.5000459	-1.1710284
H	4.2428359	1.6738642	-2.2703854
H	4.5103308	3.3224247	-1.7234722
H	4.0619407	2.7407716	1.7568857
H	3.2027000	3.7068866	0.5543785
H	4.9799037	3.6689670	0.5376266
H	5.1814945	0.2883314	-0.8161334
H	6.1042776	1.7773696	-0.5894179
H	-0.3816755	0.0567361	2.5876410
H	-1.1159909	-0.2521304	4.1927551
H	-1.0814505	-1.5295162	2.9587509
H	-3.1577344	1.4215791	4.0001665
H	-1.8175920	1.9343427	2.9829141
H	-3.3910946	-1.1372882	4.1294464
H	-4.5104797	-0.3684096	3.0093214
H	-4.3464092	-2.8743484	2.6959474
H	-2.6437232	-2.6810281	2.2710151
H	-2.6634038	-3.4446674	0.2717080
H	-4.3643941	-3.9294476	0.3258685
H	-6.0099739	-2.3832688	1.1355405
H	-5.6009536	-0.6645909	1.3442570
H	-5.6785293	-1.3569369	-0.2771711
H	-4.7959389	-2.4536996	-1.6829489
H	-3.7151013	-3.7979989	-2.0029777
H	-2.3958171	-0.6541751	-3.5635700
H	-3.6940982	-1.8130982	-3.8494899
H	-1.1959549	-3.2046950	-1.4272057
H	-1.7989594	-3.3791299	-3.0986635
H	-0.7956737	-1.9771336	-2.6346084
H	-5.0944171	-0.5539891	-2.1203240
H	-4.7592943	0.3361130	-3.5990586
H	-2.4010314	1.4588340	-3.4664330
H	-3.8223891	2.5205308	-3.3495435
H	-2.4700875	2.6938724	-2.2021280
H	-5.2257881	0.8708162	-0.4408960
H	-5.5635945	2.0198988	-1.7408252
H	-4.1038988	3.0065116	2.4881756
H	-4.6229941	1.4321705	1.8795726
H	-2.6323972	4.3575156	1.2443391
H	-1.3256055	3.1450396	1.4103005
H	-1.8027151	3.7052201	-0.1914334
H	-3.8594042	3.5752050	-0.8768196
H	-5.0817259	3.2283898	0.3328239
C	0.9445850	2.0460295	2.1048167
C	1.8903361	1.2174630	2.6865352
H	1.2672549	2.8865258	1.4929967
H	0.0185725	2.2482238	2.6306701
H	1.6239713	0.5178506	3.4736341
H	2.9266713	1.2171455	2.3750928

eTS4a^{+} : THF deprotonation with **eB**$^{+}$

76

Energy = -1856.343077868

Ca	0.0464131	0.0300345	-0.3077860
C	0.1647154	1.2685493	-2.7114140
C	-0.2721717	2.7180464	-2.9616851
H	0.1535681	0.7140556	-3.6634131
H	1.2158471	1.2556476	-2.3911680
H	0.3130273	3.2352480	-3.7377549
H	-1.3240303	2.7633361	-3.2728678
H	-0.1895902	3.3294713	-2.0526259
H	2.0673762	3.2900348	-1.1073597
C	2.7922674	2.5042310	-0.8938561
N	2.2477828	1.5923311	0.1368967
H	3.7283355	2.9662826	-0.5420451
H	2.9857769	1.9733289	-1.8251137
C	1.9234642	2.4077694	1.3310835
C	3.2519719	0.5585928	0.4796660
C	0.5764624	3.0976467	1.1748504
H	1.9060687	1.7441300	2.1999370
H	2.7105779	3.1547876	1.5183689
C	3.5309401	-0.3729173	-0.6907945
H	2.8657404	-0.0122203	1.3320248
H	4.2003595	1.0203013	0.8021911
N	-0.5326416	2.1150724	1.2283948
H	0.5239792	3.6056036	0.2077011
H	0.4531701	3.8686337	1.9512059
N	2.3291504	-1.1571016	-1.0942657
H	3.8673825	0.2049771	-1.5532980
H	4.3572969	-1.0470640	-0.4251717
C	-0.7723016	1.7083853	2.6391001
C	-1.7620354	2.7297026	0.6825030
C	2.3773680	-2.4710783	-0.4060780
C	2.3387579	-1.3572186	-2.5627599
C	-1.2846653	0.2829607	2.7447692
H	0.1605454	1.8141394	3.1965858
H	-1.4949925	2.3901565	3.1106370
H	-2.5919879	2.0304962	0.7859696
H	-2.0106454	3.6619099	1.2125833
H	-1.6209377	2.9492782	-0.3780462
C	1.1426908	-3.3163773	-0.6648785
H	3.2701682	-3.0311639	-0.7293334
H	2.4895377	-2.2809745	0.6676535
H	2.4371869	-0.3882466	-3.0526736
H	3.1699522	-2.0086855	-2.8741331
H	1.3985652	-1.8034017	-2.8927334
N	-0.3051040	-0.7083530	2.2325627
H	-2.1943177	0.1737253	2.1505736
H	-1.5426715	0.0637910	3.7936601
N	-0.0766126	-2.6981619	-0.0928778
H	1.2993984	-4.3251442	-0.2523910
H	0.9884803	-3.4383123	-1.7394064

C	-0.9804653	-2.0276449	2.1340689
C	0.8286217	-0.8176614	3.1745044
C	-1.2633536	-3.2588484	-0.7744488
C	-0.1531212	-3.0322305	1.3531510
H	-1.1875486	-2.4246770	3.1410424
H	-1.9382407	-1.8698545	1.6330471
H	1.3134092	0.1504894	3.3008689
H	0.4886363	-1.1680536	4.1617486
H	1.5729490	-1.5188528	2.7938035
H	-1.2247041	-3.0083004	-1.8363894
H	-1.2991963	-4.3541019	-0.6654463
H	-2.1681684	-2.8257061	-0.3470454
H	-0.5808499	-4.0386187	1.4808723
H	0.8629860	-3.0720644	1.7521421
H	-0.9663229	0.5330234	-2.1351567
C	-2.1833021	-0.1469756	-1.7543309
O	-2.3405503	-0.3382406	-0.2964674
C	-3.3831064	0.7173663	-2.0966054
H	-2.2968721	-1.1328064	-2.2361699
C	-3.7656512	-0.3713774	0.0380548
C	-4.5073539	0.1243081	-1.2142380
H	-3.6330692	0.6809152	-3.1612000
H	-3.2048104	1.7647459	-1.8250576
H	-4.0395672	-1.3970151	0.3126702
H	-3.9199370	0.2771872	0.9074341
H	-4.9826662	-0.7152287	-1.7300072
H	-5.2839149	0.8518075	-0.9593066

eTS4$^+$: hydrogenolysis of **eB**$^+$ with H_2
65
Energy = -1624.943241540

Ca	0.0313900	0.2083618	-0.5477047
H	1.7173261	1.8026770	-3.9303990
N	2.4123477	-0.7604050	-0.4628563
N	-0.1189478	-2.1729380	0.6041532
N	-2.2570351	-0.7402092	-1.0252802
N	-1.6779138	1.4500696	0.9097597
N	1.2612666	1.3375024	1.3368281
C	3.0063142	-0.8033869	-1.8193378
C	3.2301380	0.1181701	0.4106407
C	2.3339947	-2.1449956	0.0792006
C	0.9803638	-2.7948533	-0.1871477
C	-1.4278471	-2.7637590	0.2073418
C	0.0707200	-2.4720875	2.0406687
C	-2.0040549	-2.2017317	-1.0812737
C	-3.2094487	-0.3567270	0.0551093
C	-2.7704410	-0.3126013	-2.3478296
C	-2.5281559	0.2857827	1.2650901
C	-2.4767233	2.5162590	0.2672992
C	-1.0189862	1.9919695	2.1300244
C	2.4713067	0.5414242	1.6632344
C	1.6443164	2.7291513	1.0157156
C	0.2944205	1.2913126	2.4611481
H	2.9876005	0.1960599	-2.2582652
H	4.0450384	-1.1655441	-1.7834318
H	2.4122675	-1.4662767	-2.4509564
H	4.1694579	-0.3756514	0.7028729
H	3.5048457	0.9974324	-0.1795933
H	3.1207990	-2.7669190	-0.3690766
H	2.5361925	-2.1168975	1.1516464
H	1.0412966	-3.8716874	0.0396701
H	0.7315671	-2.6916360	-1.2498099
H	-1.3372708	-3.8565804	0.1089428
H	-2.1259285	-2.5812372	1.0294839
H	-0.7494387	-2.0322748	2.6132256
H	0.0789471	-3.5578429	2.2217292
H	1.0086594	-2.0552842	2.4070396
H	-2.9379195	-2.7452960	-1.3000333
H	-1.3216932	-2.3684293	-1.9222333
H	-3.9373297	0.3406075	-0.3653985
H	-3.7814803	-1.2282787	0.3987910
H	-2.0352311	-0.5785716	-3.1104903
H	-3.7300362	-0.7978476	-2.5808020
H	-2.9079539	0.7706741	-2.3559695
H	-1.8862712	-0.4435403	1.7698374
H	-3.3075392	0.5853076	1.9848316
H	-2.8758007	2.1681032	-0.6860915
H	-3.3125952	2.8394388	0.9062719
H	-1.8283481	3.3720559	0.0693134
H	-1.6885004	1.9209865	2.9996067
H	-0.8357097	3.0551007	1.9611850
H	3.1431482	1.1044221	2.3286815
H	2.1443560	-0.3426557	2.2184990
H	0.7846988	3.2831063	0.6349175
H	2.0419909	3.2463798	1.9019044
H	2.4098676	2.7259357	0.2380912
H	0.7174935	1.7550115	3.3667413
H	0.1173926	0.2358487	2.6937339
C	0.1549208	1.9792725	-2.4205508
C	1.4516318	2.4539164	-3.0852596
H	-0.7023818	2.1593733	-3.0858503
H	-0.0434225	2.5956542	-1.5198865
H	2.2980482	2.4203461	-2.3849931
H	0.1974411	0.4047278	-2.6235928
H	0.2184830	-0.5425608	-2.6767528
H	1.4103053	3.4819402	-3.4755222

H_2 : dihydrogen
2
Energy = -1.180087137796

H	0.0000000	0.0000000	0.0279143
H	0.0000000	0.0000000	0.7720857

LCaEt$^+$: ethylene adduct of cation **1m**$^+$
52
Energy = -1450.375961959

Ca	0.0341402	0.2293782	-0.1865753
N	-1.7739085	0.2693617	-1.9149582
N	-0.4735341	-2.1643659	-0.7062408
N	-0.5222968	-0.8452586	2.0075434

N	-1.8208603	1.5893823	0.7947871
C	-2.3785759	-1.0887421	-1.9331732
H	-3.0184331	-1.2097690	-2.8202272
H	-3.0295783	-1.1800419	-1.0596796
C	-1.3307838	-2.1989645	-1.9220694
H	-1.8362850	-3.1715017	-2.0161363
H	-0.6692112	-2.0998470	-2.7871526
C	-1.2029718	-2.7083354	0.4695204
H	-1.2595513	-3.8056554	0.4091220
H	-2.2302050	-2.3366880	0.4302097
C	-0.5571569	-2.3182359	1.7963544
H	-1.0934437	-2.8171960	2.6170452
H	0.4764170	-2.6739033	1.8300031
C	-1.8638113	-0.3367346	2.3992475
H	-2.0745259	-0.5872737	3.4497549
H	-2.6118512	-0.8566949	1.7949460
C	-1.9978656	1.1721752	2.2119299
H	-2.9761408	1.4970793	2.5960328
H	-1.2365627	1.6924892	2.7996348
C	-3.0386851	1.2825174	-0.0013608
H	-3.8355016	2.0064954	0.2263245
H	-3.4057635	0.3007048	0.3090207
C	-2.7764198	1.2895813	-1.5050760
H	-3.7275407	1.1386091	-2.0367906
H	-2.3908019	2.2648941	-1.8148234
C	-1.2277200	0.6137633	-3.2477704
H	-0.4776577	-0.1222124	-3.5461298
H	-0.7495600	1.5954624	-3.1984616
H	-2.0184222	0.6382182	-4.0115207
C	0.7689160	-2.9328004	-0.9493257
H	1.4115232	-2.8939982	-0.0667691
H	1.3081241	-2.4893768	-1.7904475
H	0.5513350	-3.9859360	-1.1788283
C	0.4791939	-0.5185892	3.0491294
H	0.5238170	0.5622083	3.2002874
H	1.4634428	-0.8644795	2.7232718
H	0.2312390	-0.9975894	4.0073490
C	-1.5121886	3.0366674	0.7347293
H	-1.3463441	3.3415969	-0.3011491
H	-0.6007540	3.2343529	1.3044504
H	-2.3319144	3.6396691	1.1513456
C	2.1985557	1.2931670	-0.5554176
C	2.6728497	2.0155540	0.7286881
H	2.1401462	2.0397286	-1.3717843
H	2.9960006	0.5963178	-0.8760988
H	1.9294737	2.7489693	1.0805793
H	2.8181795	1.3071794	1.5586696
H	3.6226084	2.5729747	0.6358138

$(LCaH_2)_2$: dimer of $LCaH_2$ with L = L4 ligand
94
Energy = -2744.792443033

Ca	-1.4276389	0.0024823	-0.0084188
H	0.0179775	0.2031757	-1.8612247
N	-2.9774856	-1.1311244	-1.8001329
N	-2.9691120	-1.7632473	1.1378892
N	-2.9615481	1.1504751	1.7880235
N	-2.9569839	1.7820085	-1.1496228
C	-4.0308485	-1.9074482	-1.1222252
H	-4.4955394	-2.6308276	-1.8164396
H	-4.8211865	-1.2166114	-0.8132052
C	-3.4908851	-2.6609476	0.0901648
H	-4.2771582	-3.3290029	0.4852925
H	-2.6497759	-3.2859637	-0.2209570
C	-4.0484768	-1.0958960	1.8850001
H	-4.5359538	-1.7970954	2.5866738
H	-4.8153030	-0.7791021	1.1716756
C	-3.5363881	0.1122128	2.6649767
H	-4.3545596	0.5181106	3.2862492
H	-2.7394360	-0.2034362	3.3432562
C	-4.0103181	1.9359572	1.1136401
H	-4.4664010	2.6633355	1.8093840
H	-4.8076781	1.2520880	0.8071362
C	-3.4675160	2.6845910	-0.1004944
H	-4.2489198	3.3595533	-0.4935246
H	-2.6200130	3.3022697	0.2079501
C	-4.0447429	1.1248343	-1.8935154
H	-4.5278452	1.8304480	-2.5938226
H	-4.8124111	0.8152362	-1.1779473
C	-3.5462130	-0.0878443	-2.6751555
H	-4.3701210	-0.4865438	-3.2934902
H	-2.7489400	0.2206780	-3.3563234
C	-2.0861472	-2.0074032	-2.5834151
H	-1.6289162	-2.7468869	-1.9241353
H	-1.2709135	-1.3890701	-2.9698275
H	-2.6336330	-2.5090354	-3.4021754
C	-2.0799664	-2.5183753	2.0406086
H	-1.6105645	-1.8334574	2.7484493
H	-1.2720396	-2.9365138	1.4341878
H	-2.6315093	-3.3125259	2.5764096
C	-2.0600496	2.0189272	2.5684705
H	-1.5987408	2.7546083	1.9078224
H	-1.2488075	1.3934185	2.9517947
H	-2.6005099	2.5249106	3.3892407
C	-2.0635396	2.5291554	-2.0547167
H	-1.6047413	1.8406126	-2.7659392
H	-1.2483879	2.9372250	-1.4510454
H	-2.6087940	3.3301916	-2.5866984
Ca	1.4286059	-0.0060634	-0.0111092
H	-0.0124711	1.8491368	0.1964380
N	2.9708678	1.1332773	1.7587602
N	2.9747957	1.7823060	-1.1547376
N	2.9559542	-1.1554897	-1.7863233
N	2.9645958	-1.8045185	1.1282215
C	4.0557407	1.8753447	1.0946710
H	4.5440534	2.5751265	1.7971590
H	4.8204397	1.1584325	0.7808456
C	3.5511858	2.6570652	-0.1154682
H	4.3737507	3.2737618	-0.5194316
H	2.7568682	3.3396870	0.1974547

C	4.0224836	1.1018703	-1.9364861
H	4.4850334	1.7949990	-2.6622838
H	4.8157185	0.7909831	-1.2497912
C	3.4758462	-0.1094058	-2.6870203
H	4.2578465	-0.5066764	-3.3587855
H	2.6325551	0.2036862	-3.3080900
C	4.0369451	-1.9058149	-1.1252048
H	4.5177122	-2.6094806	-1.8290245
H	4.8081709	-1.1949273	-0.8136150
C	3.5297537	-2.6835898	0.0863655
H	4.3481841	-3.3077229	0.4873045
H	2.7282064	-3.3588924	-0.2240341
C	4.0203656	-1.1322515	1.9061129
H	4.4799677	-1.8286458	2.6306595
H	4.8136355	-0.8278155	1.2166052
C	3.4854269	0.0833777	2.6580907
H	4.2724900	0.4747084	3.3274201
H	2.6416490	-0.2230842	3.2818010
C	2.0834387	2.0398551	2.5112019
H	1.6218144	2.7519092	1.8253895
H	1.2697431	1.4375102	2.9241433
H	2.6340819	2.5708714	3.3091661
C	2.0799391	2.5665373	-2.0265395
H	1.6155080	1.9072401	-2.7615052
H	1.2701398	2.9566883	-1.4034856
H	2.6263767	3.3827357	-2.5334798
C	2.0597613	-2.0557064	-2.5361107
H	1.5908839	-2.7608087	-1.8479887
H	1.2519029	-1.4468845	-2.9508499
H	2.6054234	-2.5944038	-3.3323702
C	2.0672169	-2.5826760	2.0029934
H	1.6157494	-1.9216754	2.7443792
H	1.2485497	-2.9606731	1.3838403
H	2.6083999	-3.4070061	2.5023157
H	0.0214224	-0.2093558	1.8421342
H	-0.0270786	-1.8564686	-0.2155101

$LCaH_2$: ligand L4 stabilized CaH_2 molecule
47
Energy = -1372.391424598

Ca	-0.0276110	-0.0875855	1.4120334
H	-1.5668050	-0.8969084	2.7256022
N	-2.0366360	0.5155845	-0.0750043
N	0.4688769	2.0665512	0.0201703
N	2.0318094	-0.5653155	-0.0497650
N	-0.4720725	-2.1161746	-0.1737164
C	-1.6362073	1.4388881	-1.1584639
H	-2.5211202	1.8631122	-1.6609637
H	-1.0877463	0.8672593	-1.9118936
C	-0.7615306	2.5726926	-0.6349875
H	-0.5173396	3.2591564	-1.4615077
H	-1.3109005	3.1555884	0.1090250
C	1.4763213	1.6888605	-1.0005557
H	1.9581123	2.5926200	-1.4083658
H	0.9532516	1.2107982	-1.8331416
C	2.5562535	0.7600619	-0.4565476
H	3.3498343	0.6504214	-1.2143130
H	3.0109256	1.2029942	0.4338101
C	1.6716236	-1.3935491	-1.2203945
H	2.5745526	-1.7749060	-1.7251496
H	1.1491950	-0.7597291	-1.9419769
C	0.7813094	-2.5664320	-0.8254050
H	0.5679659	-3.1807333	-1.7146654
H	1.3054535	-3.2101706	-0.1139761
C	-1.4440080	-1.6511517	-1.1927016
H	-1.9101112	-2.5159282	-1.6925878
H	-0.8929919	-1.1043464	-1.9625379
C	-2.5438356	-0.7703806	-0.6093838
H	-3.3114302	-0.5969109	-1.3819250
H	-3.0276043	-1.2862289	0.2245861
C	-3.0816620	1.1290019	0.7719072
H	-2.7218396	2.0738642	1.1860983
H	-3.2932212	0.4500108	1.6008155
H	-3.9989672	1.3263142	0.1938269
C	1.0202498	3.1083444	0.9137903
H	1.8903166	2.7148742	1.4405505
H	0.2684078	3.3717256	1.6619635
H	1.2986394	4.0120203	0.3471828
C	3.0481035	-1.2480485	0.7791450
H	2.6766420	-2.2242024	1.0999382
H	3.2281540	-0.6406902	1.6689018
H	3.9857510	-1.3962214	0.2193587
C	-1.0527748	-3.2284768	0.6093563
H	-1.9411584	-2.8792706	1.1368790
H	-0.3269759	-3.5549118	1.3584733
H	-1.3103330	-4.0808921	-0.0402857
H	1.4645124	0.6033190	2.8424593

mA$^+$: 1-hexene adduct of monomer **1m**$^+$•THF
77
Energy = -1840.340597004

Ca	0.0425079	-0.3806853	0.4349919
N	2.0566771	0.4536086	1.9622082
N	0.0129201	-1.5996426	2.6303939
N	-0.1990899	2.1811223	0.7790838
H	-2.8079526	-1.8749718	-1.7011158
C	2.1743537	-0.4623755	3.1266289
C	3.3690825	0.5206391	1.2837532
C	1.6722871	1.8142563	2.4164324
C	0.8191122	-0.9078777	3.6620552
C	-1.4148851	-1.6867072	3.0303482
C	0.5362761	-2.9636332	2.3917075
C	-1.2777371	2.4199670	1.7726968
C	1.1009007	2.6808468	1.2989616
C	-0.5224952	2.8905097	-0.4782485
H	2.7473227	0.0095048	3.9401555
H	2.7509265	-1.3310220	2.7964365
H	3.6429651	-0.4730146	0.9232202
H	4.1516890	0.8778699	1.9707800
H	3.3154012	1.1973225	0.4302593

H	0.9414752	1.7117836	3.2219519
H	2.5445580	2.3283608	2.8496927
H	0.2535834	-0.0413834	4.0141631
H	0.9754249	-1.5609006	4.5351379
C	-2.1399777	-0.3486852	2.9229419
H	-1.8847293	-2.4175829	2.3667950
H	-1.5080370	-2.0669644	4.0597429
H	1.5794419	-2.9135351	2.0690982
H	-0.0499480	-3.4299656	1.5979765
H	0.4840843	-3.5761641	3.3047168
C	-2.5209443	1.5901366	1.4869150
H	-0.8908095	2.1728783	2.7638713
H	-1.5463510	3.4875478	1.7928206
H	1.7972191	2.7114668	0.4555116
H	0.9983125	3.7140811	1.6663176
H	-1.4492143	2.4978170	-0.9036609
H	0.2833650	2.7397136	-1.1982508
H	-0.6464314	3.9708021	-0.3092545
N	-2.2388952	0.1348241	1.5221717
H	-1.6189989	0.4102033	3.5125365
H	-3.1458387	-0.4517043	3.3594175
H	-3.3104991	1.8600839	2.2053187
H	-2.9080619	1.8217895	0.4904999
C	-3.3248996	-0.5863662	0.8227766
H	-3.3833691	-0.2312907	-0.2081927
H	-4.2937853	-0.4158386	1.3178057
H	-3.1048980	-1.6535374	0.8011651
C	-1.7638043	-2.0389817	-2.0067617
C	-0.9061454	-2.3718716	-0.7648493
H	-1.8081507	-2.8508045	-2.7573465
H	-1.4625839	-3.1015881	-0.1443494
H	-0.0123200	-2.9366062	-1.1030802
H	2.5404686	1.6510290	-1.7667380
C	2.0214769	0.8396791	-2.2893876
C	2.9039770	0.1854058	-3.3499734
H	1.0724407	1.2074961	-2.6875005
O	1.7285021	-0.2051890	-1.3081221
C	3.5927033	-0.9403022	-2.5639830
H	3.6060120	0.8967984	-3.7901229
H	2.2873638	-0.2356709	-4.1505318
C	2.4629374	-1.4202706	-1.6675495
H	4.4191945	-0.5446040	-1.9644456
H	3.9739738	-1.7373114	-3.2062224
H	1.7787041	-2.0961096	-2.1901270
H	2.7926096	-1.8910462	-0.7375833
C	-1.2877690	-0.7732831	-2.7343708
C	-2.1053936	-0.3763658	-3.9662502
H	-0.2363176	-0.9014197	-3.0257119
H	-1.3034690	0.0745198	-2.0239728
C	-1.5954854	0.9033694	-4.6393456
H	-2.0868980	-1.2008552	-4.6927755
H	-3.1580764	-0.2414968	-3.6779087
C	-2.4121248	1.3046759	-5.8717731
H	-1.6091423	1.7246124	-3.9079452
H	-0.5438077	0.7630191	-4.9265657
H	-2.0243070	2.2218812	-6.3286994
H	-2.3871071	0.5131951	-6.6300081
H	-3.4614264	1.4780568	-5.6049407

mB$^+$: 1-hexene adduct of monomer **2m$^+$**

75

Energy = -1781.122878642

Ca	-0.7658194	0.0154857	-0.3932530
H	2.7463768	-0.9182255	-1.0067343
N	-0.3941613	2.3598087	0.6407307
N	-0.8740490	-0.1495764	2.2020762
N	-0.5260699	-2.4689488	0.3633915
N	-2.7707897	-1.2831001	-1.2192810
N	-2.5042765	1.6347325	-1.4079178
C	1.0091272	2.7288462	0.3481004
C	-1.3236900	3.3312606	0.0083714
C	-0.6192288	2.3244250	2.1069032
C	-0.1337552	1.0229063	2.7292441
C	-0.1747444	-1.4013689	2.5842355
C	-2.2527521	-0.1541253	2.7336936
C	-0.7152974	-2.6076869	1.8282831
C	-1.5353580	-3.2697416	-0.3721669
C	0.8362838	-2.9087846	-0.0106678
C	-2.8760514	-2.5472502	-0.4503093
C	-2.7047579	-1.5735387	-2.6673556
C	-3.9056475	-0.3853040	-0.8978392
C	-2.6525485	2.6866786	-0.3692986
C	-2.0143899	2.2187854	-2.6782283
C	-3.8112906	0.9493120	-1.6317219
H	1.1776770	2.6739522	-0.7292948
H	1.2414130	3.7417040	0.7101868
H	1.6860528	2.0214996	0.8330138
H	-1.5130302	4.1827263	0.6774244
H	-0.8374691	3.7355882	-0.8816712
H	-0.1118295	3.1717442	2.5929791
H	-1.6895520	2.4512776	2.2886483
H	-0.2264101	1.0848412	3.8250934
H	0.9269827	0.8714856	2.5040167
H	0.8880522	-1.2646147	2.3583859
H	-0.2561877	-1.5811516	3.6679429
H	-2.8032879	-1.0130917	2.3474082
H	-2.2549183	-0.2030253	3.8333772
H	-2.7802704	0.7500992	2.4246347
H	-1.7820227	-2.7297035	2.0295926
H	-0.2186962	-3.5183410	2.1958289
H	-1.1439339	-3.4473296	-1.3774759
H	-1.6750722	-4.2568313	0.0942650
H	1.5749547	-2.3368663	0.5558573
H	0.9833843	-3.9789154	0.2015823
H	0.9980493	-2.7165902	-1.0730149
H	-3.2257085	-2.2938878	0.5560998
H	-3.6322172	-3.2143946	-0.8920011
H	-1.9180735	-2.3048789	-2.8565639
H	-3.6603233	-1.9739664	-3.0386299
H	-2.4561195	-0.6701369	-3.2261853

H	-3.8989274	-0.2258156	0.1873246
H	-4.8699859	-0.8564986	-1.1488700
H	-3.3447831	3.4705771	-0.7169474
H	-3.1094014	2.2217498	0.5126714
H	-1.9824883	1.4378141	-3.4401012
H	-2.6663139	3.0338219	-3.0274615
H	-1.0000401	2.6004204	-2.5548105
H	-3.9344562	0.7889730	-2.7048019
H	-4.6445921	1.5889198	-1.3131938
C	1.0589038	-0.0106618	-2.0452192
C	2.5495595	-0.0621805	-1.6735831
H	0.8405498	-0.8871574	-2.6951612
H	0.9096289	0.8612904	-2.7172774
H	2.8183804	0.8284417	-1.0819693
C	3.5438958	-0.1565218	-2.8466106
C	5.0152965	-0.2062030	-2.4182413
H	3.3860210	0.7036312	-3.5147205
H	3.3057795	-1.0528700	-3.4390767
C	5.9985987	-0.3057501	-3.5905144
H	5.2510460	0.6911271	-1.8264013
H	5.1677853	-1.0647463	-1.7466996
C	7.4635362	-0.3562399	-3.1429600
H	5.7641347	-1.2022736	-4.1810356
H	5.8485542	0.5529867	-4.2595769
H	8.1436901	-0.4271977	-3.9992928
H	7.7298366	0.5436010	-2.5752046
H	7.6447736	-1.2234984	-2.4964190

mTS1$^+$: 1-hexene addition to **1m**$^+$•THF
77
Energy = -1840.308684854

Ca	-0.1413122	-0.1873858	0.3640573
N	1.9565861	0.5715373	1.8723206
N	0.0232925	-1.6816249	2.4967788
N	-0.3771587	2.2931107	1.0223127
H	-1.4241732	0.3134534	-1.2416225
C	2.1896597	-0.4853028	2.8873777
C	3.2158172	0.7904556	1.1296495
C	1.5611200	1.8406615	2.5323381
C	0.8986438	-1.0158554	3.4938444
C	-1.3525487	-1.8218151	3.0425450
C	0.5650881	-3.0264237	2.1975831
C	-1.4407219	2.3692071	2.0557056
C	0.9006858	2.8142947	1.5665897
C	-0.7656482	3.1052955	-0.1535637
H	2.8382007	-0.1134858	3.6970170
H	2.7342897	-1.2952827	2.3934270
H	3.5200192	-0.1390367	0.6472113
H	4.0199248	1.1206239	1.8058752
H	3.0723976	1.5507806	0.3605793
H	0.8751957	1.6052033	3.3490493
H	2.4403145	2.3228455	2.9884937
H	0.3380875	-0.1968458	3.9498658
H	1.1473140	-1.7175606	4.3056903
C	-2.1146819	-0.5003551	3.0671028
H	-1.8782263	-2.5427226	2.4098567
H	-1.3252065	-2.2446685	4.0592787
H	1.5548841	-2.9397290	1.7425984
H	-0.0952810	-3.5367490	1.4959230
H	0.6544059	-3.6311384	3.1131831
C	-2.6432580	1.4928833	1.7342568
H	-1.0089214	2.0735458	3.0152614
H	-1.7802052	3.4109254	2.1713561
H	1.5619694	3.0063715	0.7165777
H	0.7452108	3.7795302	2.0744647
H	-1.6770825	2.7001456	-0.5933027
H	0.0241104	3.0448188	-0.9056678
H	-0.9138746	4.1602723	0.1279698
N	-2.3233947	0.0435795	1.7051900
H	-1.5652707	0.2393169	3.6548934
H	-3.0792958	-0.6531881	3.5765159
H	-3.4428134	1.7010326	2.4636047
H	-3.0294392	1.7444211	0.7428628
C	-3.4494743	-0.6567186	1.0518143
H	-3.5435792	-0.2796366	0.0321039
H	-4.3898349	-0.4867030	1.5993822
H	-3.2570783	-1.7304046	1.0156556
C	-1.6120110	-1.5736463	-1.7421669
C	-0.9993701	-2.4219574	-0.8513432
H	-2.6713162	-1.3762775	-1.6091248
H	-1.5464593	-2.8343105	-0.0076572
H	-0.0078308	-2.8284819	-1.0429752
H	2.1622705	1.6720149	-1.8028851
C	1.7057852	0.8082242	-2.3003811
C	2.5952883	0.2667473	-3.4147753
H	0.6874845	1.0566642	-2.6094670
O	1.6059685	-0.2725143	-1.3151577
C	3.4943628	-0.7299324	-2.6660912
H	3.1560321	1.0610962	-3.9121227
H	1.9946299	-0.2569850	-4.1649562
C	2.5129985	-1.3544724	-1.6847366
H	4.2908724	-0.2035115	-2.1298845
H	3.9474768	-1.4747091	-3.3241730
H	1.9283683	-2.1561735	-2.1503911
H	2.9718373	-1.7320645	-0.7669752
C	-1.1034360	-1.3556710	-3.1485589
C	-1.7200143	-0.1767243	-3.9037376
H	-1.3250945	-2.2786256	-3.7081357
H	-0.0109986	-1.2706251	-3.1368053
C	-1.2053329	-0.0534656	-5.3414356
H	-2.8133435	-0.2934002	-3.9187873
H	-1.5174731	0.7435188	-3.3429640
C	-1.8133101	1.1392532	-6.0862598
H	-0.1107826	0.0464752	-5.3267618
H	-1.4213340	-0.9797601	-5.8912856
H	-1.4269554	1.2132089	-7.1087121
H	-2.9045145	1.0482652	-6.1435179
H	-1.5844632	2.0794091	-5.5699887

mTS2$^+$: hydrogenolysis of **mA**$^+$ with H_2

79

Energy = -1841.507891323

Ca	0.2345697	0.5059643	-0.1286292
N	1.2520528	1.0869170	2.1346441
N	-1.6117678	2.3229815	-0.7807842
N	1.3214300	2.8226483	-0.3724735
C	0.2657988	0.8360411	3.2167107
C	2.4538609	0.2571217	2.3847395
C	1.6359892	2.5226298	2.1024808
C	-0.8972298	3.5453538	-1.2294089
C	-2.4951130	1.8643164	-1.8753859
C	-2.4358675	2.6384757	0.4105181
C	0.2880671	3.8842169	-0.3351409
C	2.2586277	2.9379992	0.7751072
C	2.1041399	2.9470228	-1.6232982
C	-1.1249558	1.3511408	2.8737063
H	0.5998372	1.2944919	4.1606762
H	0.2356641	-0.2452468	3.3807538
H	2.1680913	-0.7965735	2.3813778
H	2.9085226	0.5082040	3.3551511
H	3.1821398	0.4187835	1.5888227
H	0.7439493	3.1212042	2.3018221
H	2.3482597	2.7406589	2.9133398
H	-1.5851357	4.4047532	-1.2675028
H	-0.5558809	3.3651801	-2.2525099
H	-1.8876391	1.6369383	-2.7521748
H	-3.2379048	2.6346749	-2.1342016
H	-3.0264819	0.9587376	-1.5735706
C	-2.8717291	1.3979602	1.1714485
H	-1.8586688	3.2963270	1.0638680
H	-3.3332005	3.2035958	0.1119582
H	-0.0451809	4.0080207	0.6980823
H	0.7142139	4.8503868	-0.6476551
H	3.1101274	2.2911912	0.5452172
H	2.6363270	3.9692952	0.8636538
H	1.4428903	2.8692519	-2.4888886
H	2.8303405	2.1324402	-1.6615178
H	2.6232661	3.9170049	-1.6668731
N	-1.7399247	0.6164469	1.7361163
H	-1.0725845	2.4102573	2.6133610
H	-1.7692442	1.2775703	3.7636518
H	-3.4258867	0.7252866	0.5099075
H	-3.5651220	1.6981198	1.9735697
C	-2.3096463	-0.6521189	2.2485570
H	-1.5365519	-1.2442588	2.7380902
H	-2.7206810	-1.2308146	1.4186094
H	-3.1117037	-0.4492354	2.9747245
H	-2.0001380	-0.7434748	-3.2936721
C	-1.1642230	-1.2385710	-2.7866889
C	-0.4248183	-2.1872676	-3.7173581
H	-1.5243062	-1.7065607	-1.8674279
O	-0.1824064	-0.2192910	-2.4141855
C	0.5037040	-1.2236097	-4.4712754
H	-1.1065994	-2.7292427	-4.3769300
H	0.1580240	-2.9114035	-3.1397143
C	0.9285098	-0.2482648	-3.3773392
H	-0.0491802	-0.7021265	-5.2592137
H	1.3634308	-1.7236688	-4.9228589
H	1.8190470	-0.5850809	-2.8396961
H	1.0796270	0.7720861	-3.7370320
H	0.0853395	-2.1111285	1.6451512
C	0.0112525	-2.3352189	0.5703076
C	1.3071353	-1.9214168	-0.1245190
H	-0.8541374	-1.7370897	0.2104209
H	2.1654840	-2.3393626	0.4184148
H	1.3371336	-2.3460845	-1.1402491
H	2.0383167	-0.5525118	-0.4088330
H	2.4321827	0.2774374	-0.6148996
C	-0.4191617	-3.8100624	0.4172355
C	0.5688265	-4.7952881	1.0458958
H	-0.5223193	-4.0335425	-0.6543430
H	-1.4125118	-3.9553257	0.8682395
C	0.1544856	-6.2605718	0.8714314
H	1.5618574	-4.6435250	0.6030591
H	0.6687788	-4.5708684	2.1184906
C	1.1515833	-7.2388642	1.5012744
H	-0.8396676	-6.4124440	1.3139299
H	0.0530274	-6.4791757	-0.2006146
H	0.8358436	-8.2784497	1.3603946
H	2.1461418	-7.1247881	1.0536500
H	1.2480211	-7.0589921	2.5787513

mTS3$^+$: 1-hexene addition to **2m$^+$**

75

Energy = -1781.088856634

Ca	-0.2635467	0.0753527	-0.2495361
H	1.7338752	0.5807786	0.1747727
N	-0.6451854	2.4564561	0.8318525
N	-0.3884459	-0.2203032	2.2911340
N	0.5232115	-2.3629106	0.3623365
N	-1.9510246	-1.6776244	-1.1083089
N	-2.4765619	1.2377861	-1.2405519
C	0.5481628	3.2981449	0.5941246
C	-1.8558236	3.1206904	0.2913698
C	-0.8118727	2.2339286	2.2909623
C	0.0596570	1.0995363	2.8044610
C	0.6695443	-1.2249745	2.5706842
C	-1.6544455	-0.6144052	2.9410219
C	0.4241349	-2.5321602	1.8335384
C	-0.3064508	-3.3735531	-0.3325093
C	1.9338692	-2.4828706	-0.0569052
C	-1.7682796	-2.9492667	-0.3670387
C	-1.8387200	-1.9209129	-2.5619147
C	-3.2694066	-1.0932351	-0.7660104
C	-2.9138892	2.1062368	-0.1164832
C	-2.2717009	2.0459970	-2.4635442
C	-3.5302173	0.2150156	-1.4984602
H	0.6529511	3.4769816	-0.4789654
H	0.4617144	4.2677064	1.1094414
H	1.4363775	2.7652465	0.9391762

H	-2.2861775	3.8169566	1.0262528
H	-1.5601394	3.7221376	-0.5709306
H	-0.5728497	3.1576144	2.8406176
H	-1.8662510	2.0181292	2.4836039
H	0.0587277	1.1034532	3.9065885
H	1.0881972	1.2386925	2.4564282
H	1.6129359	-0.7832420	2.2327017
H	0.7411902	-1.4187991	3.6533770
H	-1.9863248	-1.5840019	2.5666639
H	-1.5363649	-0.6826238	4.0337990
H	-2.4364341	0.1146435	2.7206978
H	-0.5691399	-2.9209229	2.0711470
H	1.1476077	-3.2869389	2.1781190
H	0.0813867	-3.4794591	-1.3491289
H	-0.2204637	-4.3616446	0.1452959
H	2.5141008	-1.6836749	0.4089746
H	2.3553327	-3.4624831	0.2189396
H	1.9932739	-2.3634796	-1.1406544
H	-2.1355133	-2.7904088	0.6524448
H	-2.3817503	-3.7512428	-0.8069366
H	-0.9123988	-2.4566966	-2.7705122
H	-2.6855658	-2.5184054	-2.9342918
H	-1.8027247	-0.9753037	-3.1049236
H	-3.2885083	-0.9380941	0.3196467
H	-4.0853353	-1.7951765	-1.0055460
H	-3.8395354	2.6384609	-0.3903475
H	-3.1568578	1.4552754	0.7323054
H	-2.0503099	1.3774905	-3.2980406
H	-3.1648915	2.6394675	-2.7128920
H	-1.4257592	2.7241558	-2.3379883
H	-3.5722951	0.0314823	-2.5738206
H	-4.5190438	0.5961366	-1.2073459
C	1.0656786	0.3546374	-2.5434161
C	2.0658820	0.8675574	-1.7515837
H	1.1420378	-0.6410697	-2.9761816
H	0.2422431	0.9775212	-2.8855818
H	2.0182280	1.9230934	-1.4932373
C	3.4544706	0.2766342	-1.6958991
C	4.3128189	0.6574872	-0.4881671
H	3.9665742	0.6315511	-2.6052240
H	3.4007901	-0.8136953	-1.7955148
C	5.7352226	0.0943541	-0.5722501
H	4.3563855	1.7533574	-0.4080660
H	3.8151525	0.3071902	0.4235680
C	6.5868612	0.4614994	0.6473021
H	5.6838595	-0.9995463	-0.6670026
H	6.2243790	0.4607066	-1.4854100
H	7.5974173	0.0452257	0.5715111
H	6.6772188	1.5498539	0.7469920
H	6.1328755	0.0793188	1.5696184

mTS4$^+$: hydrogenolysis of **mB$^+$** with H_2
77
Energy = -1782.289112911

Ca	-0.5637026	-0.1656132	-0.5938189
H	2.6336118	-1.3788079	-1.2675664
N	0.2995326	2.2419652	0.4635444
N	-0.1091184	-0.3780781	1.9906219
N	-0.4474216	-2.6792374	0.1132473
N	-2.9431452	-1.1840148	-0.7069739
N	-2.3272405	1.6743034	-0.9681057
C	1.6491126	2.7191183	0.0883035
C	-0.6810410	3.2768920	0.0500041
C	0.2620284	2.0793364	1.9399681
C	0.8065668	0.7275361	2.3710918
C	0.6056436	-1.6741870	2.1295761
C	-1.2792660	-0.3541260	2.8964873
C	-0.2052416	-2.8336819	1.5713608
C	-1.7366108	-3.3072666	-0.2716260
C	0.6481785	-3.3322383	-0.6385246
C	-2.9279040	-2.4313300	0.0933399
C	-3.4710342	-1.4884321	-2.0563920
C	-3.7762114	-0.1488044	-0.0550565
C	-2.1112926	2.7640228	0.0166237
C	-2.0952425	2.1537274	-2.3510367
C	-3.7252831	1.1660044	-0.8230200
H	1.7257528	2.7915650	-0.9971946
H	1.8516206	3.7056301	0.5329579
H	2.4036459	2.0159665	0.4436556
H	-0.6327554	4.1459018	0.7249448
H	-0.3864508	3.6337509	-0.9399842
H	0.8390102	2.8826687	2.4226952
H	-0.7704477	2.1910918	2.2779854
H	0.9809096	0.7235751	3.4583265
H	1.7711762	0.5419794	1.8863880
H	1.5522335	-1.5866015	1.5839664
H	0.8513169	-1.8694670	3.1854684
H	-1.9740402	-1.1546148	2.6452540
H	-0.9660038	-0.4783667	3.9442265
H	-1.8101911	0.5941244	2.8020227
H	-1.1680981	-2.8995818	2.0820638
H	0.3216182	-3.7768058	1.7748040
H	-1.7066435	-3.4633453	-1.3529327
H	-1.8426726	-4.2962571	0.2001685
H	1.6091606	-2.9506795	-0.2876707
H	0.6329629	-4.4229628	-0.4934003
H	0.5403370	-3.1034604	-1.7015026
H	-2.8863348	-2.1544497	1.1510464
H	-3.8609673	-2.9985519	-0.0486623
H	-2.9255144	-2.3323377	-2.4762250
H	-4.5445134	-1.7322000	-2.0058915
H	-3.3219753	-0.6434266	-2.7279721
H	-3.3993132	-0.0064089	0.9646010
H	-4.8266413	-0.4735104	0.0286408
H	-2.7796222	3.6114799	-0.2051270
H	-2.4032807	2.3773203	0.9992520
H	-2.3149306	1.3425059	-3.0486105
H	-2.7284973	3.0195611	-2.5949394
H	-1.0484961	2.4354923	-2.4830859
H	-4.1527111	1.0340930	-1.8188176

H	-4.3494978	1.9081001	-0.3102351
C	1.4178891	0.1085743	-2.2551721
C	2.4825482	-0.2920738	-1.2310253
H	1.7576225	-0.1827838	-3.2591964
H	1.3062446	1.2064291	-2.2884553
H	2.1330666	-0.0858311	-0.1977414
C	3.8631767	0.3738187	-1.3786342
C	4.8200175	0.0422702	-0.2285563
H	3.7376706	1.4622392	-1.4499478
H	4.3029862	0.0526934	-2.3325700
C	6.1966577	0.7024037	-0.3640077
H	4.3637308	0.3579693	0.7236009
H	4.9440128	-1.0481089	-0.1588329
C	7.1347844	0.3733730	0.8018528
H	6.6562523	0.3815770	-1.3084916
H	6.0671266	1.7910088	-0.4365105
H	8.1094753	0.8582340	0.6797628
H	6.7083951	0.7104434	1.7545365
H	7.3026953	-0.7077112	0.8754612
H	0.1148750	-0.7124609	-2.5051950
H	-0.7198682	-1.1859047	-2.5847562

mTS5a^{+} : H_2 exchange via cation **1m**$^{+}$
48
Energy = -1372.873147233

Ca	-1.2354968	0.0015701	0.0002796
H	-3.1693212	1.0399799	-0.0278310
N	0.0774705	1.6593122	1.3248891
N	0.0767366	-1.3148603	1.6707094
N	0.0738297	-1.6588191	-1.3245230
N	0.0791405	1.3153037	-1.6703210
C	1.1663190	0.9129693	2.0091634
H	1.6398978	1.5451922	2.7749987
H	1.9365941	0.6828757	1.2683792
C	0.6675700	-0.3731393	2.6603877
H	1.4958216	-0.8487702	3.2057053
H	-0.1090773	-0.1462336	3.3957617
C	1.1476265	-2.0153448	0.9119668
H	1.6116679	-2.7909976	1.5392366
H	1.9294480	-1.2870462	0.6806434
C	0.6330897	-2.6571054	-0.3728772
H	1.4479669	-3.2281526	-0.8418904
H	-0.1655356	-3.3680701	-0.1447006
C	1.1640811	-0.9147424	-2.0089946
H	1.6361811	-1.5478844	-2.7750018
H	1.9350000	-0.6862618	-1.2683802
C	0.6678584	0.3723938	-2.6601297
H	1.4969822	0.8463181	-3.2056087
H	-0.1093959	0.1470762	-3.3953479
C	1.1516155	2.0135598	-0.9117803
H	1.6171468	2.7882490	-1.5391349
H	1.9319697	1.2836554	-0.6805644
C	0.6385913	2.6564039	0.3731263
H	1.4547147	3.2258630	0.8419339
H	-0.1586075	3.3689925	0.1450544
C	-0.7778919	2.3505285	2.3186142
H	-1.2110699	1.6227618	3.0083725
H	-1.5910704	2.8611006	1.7976966
H	-0.2005286	3.0842938	2.8997197
C	-0.7835639	-2.2973523	2.3722834
H	-1.2407427	-2.9763335	1.6498579
H	-1.5801198	-1.7660537	2.8988394
H	-0.2045704	-2.8867497	3.0980736
C	-0.7831435	-2.3482840	-2.3180873
H	-1.2148630	-1.6196557	-3.0078458
H	-1.5973562	-2.8570530	-1.7970294
H	-0.2074055	-3.0833271	-2.8991905
C	-0.7792500	2.2996057	-2.3717099
H	-1.2353868	2.9790941	-1.6491185
H	-1.5766669	1.7699287	-2.8985775
H	-0.1990142	2.8882003	-3.0971633
H	-3.3340958	0.0048023	-0.0035780
H	-3.1728051	-1.0306594	0.0213304

mTS5^{+} : H_2 exchange via cation **1m**$^{+}$•THF
61
Energy = -1605.491394244

Ca	0.2150446	-0.2583944	-0.3177775
N	-0.1435846	1.5481475	1.4993699
C	-0.0141177	0.8857268	2.8236358
C	-1.5196622	2.0736491	1.3106125
C	0.8301617	2.6604530	1.4246103
C	-0.6801397	-0.4851058	2.8667561
H	-0.4382840	1.5172621	3.6196044
H	1.0552722	0.7893322	3.0316065
H	-1.6663473	2.9862145	1.9088895
H	-2.2242881	1.3305119	1.6924150
C	-1.8268879	2.3786290	-0.1508132
H	0.7592555	3.1563848	0.4543138
H	1.8399706	2.2622193	1.5338575
H	0.6422081	3.4046071	2.2133466
N	-0.0945516	-1.4315756	1.8849753
H	-0.5973630	-0.8908879	3.8868841
H	-1.7476203	-0.3901092	2.6521038
N	-1.7721821	1.1641831	-1.0058799
H	-2.8135581	2.8613374	-0.2216048
H	-1.0965748	3.0894531	-0.5475191
C	-1.0050401	-2.5849172	1.6559182
C	1.2133640	-1.9292972	2.3682057
C	-2.9941251	0.3415173	-0.8033324
C	-1.6666938	1.5696830	-2.4267124
C	-2.1922542	-2.2244220	0.7683325
H	-1.3723506	-2.9857659	2.6130877
H	-0.4109149	-3.3699233	1.1805202
H	1.8940811	-1.0906841	2.5349078
H	1.6432586	-2.5838952	1.6063102
H	1.1070481	-2.4831522	3.3131708
H	-3.8515021	0.8083698	-1.3124006
H	-3.2268274	0.3383829	0.2646087
C	-2.8364114	-1.0879914	-1.3056433

H	-1.5881653	0.6845334	-3.0593631
H	-0.7632438	2.1697603	-2.5632761
H	-2.5386548	2.1659657	-2.7363875
N	-1.7661944	-1.8345235	-0.5979720
H	-2.8835100	-3.0806448	0.7272768
H	-2.7493440	-1.3919332	1.2066145
H	-3.8037315	-1.6070069	-1.2144271
H	-2.5715034	-1.0847881	-2.3666136
C	-1.4315128	-3.0423214	-1.3864077
H	-0.6454078	-3.6068969	-0.8842957
H	-1.0606112	-2.7306686	-2.3650409
H	-2.3160749	-3.6849856	-1.5147682
H	3.1624002	2.5147746	-1.2871979
C	2.6766714	1.6230477	-1.7012276
C	3.6068349	0.8396432	-2.6365044
O	2.3801763	0.7149793	-0.6031899
H	1.7254153	1.9056853	-2.1574102
C	4.2093067	-0.2745147	-1.7347004
H	4.3754502	1.4927210	-3.0559656
H	3.0402500	0.3996099	-3.4592103
C	3.6279254	0.0083485	-0.3476385
H	3.8879154	-1.2589647	-2.0812295
H	5.3012761	-0.2514429	-1.7182431
H	3.3784020	-0.8886549	0.2207866
H	4.2759771	0.6636179	0.2475562
H	1.3665748	-2.0536844	-0.9641267
H	1.0242974	-1.5362245	-1.8069245
H	0.5595979	-0.8528827	-2.4380872

mTS6$^+$: H_2 exchange via L5-stabilized **2m$^+$**
59
Energy = -1546.271105633

Ca	-0.0427174	-0.0144745	-0.7218305
N	0.8870661	2.3942960	-0.4353128
N	1.9703479	-0.1591117	0.8046833
N	0.6836938	-2.5163963	-0.5745388
N	-1.8762000	-1.3692382	0.3890249
N	-1.9466765	1.5497452	0.0863020
C	1.4467570	2.8716513	-1.7215145
C	-0.1791038	3.3209629	0.0252955
C	1.9640541	2.3236977	0.5863940
C	2.7790506	1.0438849	0.4761127
C	2.6959420	-1.3770576	0.3561445
C	1.7221037	-0.2172589	2.2607524
C	1.8103391	-2.6139418	0.3892917
C	-0.4573279	-3.3463817	-0.1160699
C	1.1299304	-2.9732016	-1.9108363
C	-1.2870064	-2.6176440	0.9332000
C	-3.0066107	-1.6902705	-0.5097832
C	-2.3098756	-0.4863969	1.4985189
C	-1.2331674	2.6026461	0.8556692
C	-2.6856240	2.1399063	-1.0547236
C	-2.8902369	0.8279495	0.9901291
H	0.6617311	2.8632666	-2.4820300
H	1.8456383	3.8929463	-1.6255858
H	2.2412729	2.1980429	-2.0456953
H	0.2465794	4.1453923	0.6149203
H	-0.6407218	3.7730771	-0.8548511
H	2.6331291	3.1924534	0.4912630
H	1.4988472	2.3908116	1.5735814
H	3.6569752	1.1101702	1.1377729
H	3.1399815	0.9189424	-0.5492613
H	3.0299996	-1.1889678	-0.6689745
H	3.5873387	-1.5464503	0.9806009
H	1.1154470	-1.0901401	2.5070682
H	2.6672407	-0.2778552	2.8214181
H	1.1802037	0.6723980	2.5873456
H	1.3961791	-2.7552051	1.3905674
H	2.4203927	-3.5034838	0.1722093
H	-1.0708114	-3.5770740	-0.9907136
H	-0.1104045	-4.3081196	0.2908588
H	1.9880816	-2.3789669	-2.2285075
H	1.4074792	-4.0382312	-1.8941603
H	0.3222171	-2.8237421	-2.6318488
H	-0.6562127	-2.3395767	1.7845603
H	-2.0711130	-3.2861841	1.3208067
H	-2.6877571	-2.4258280	-1.2487373
H	-3.8597535	-2.0985394	0.0531617
H	-3.3286315	-0.7990504	-1.0500314
H	-1.4329152	-0.2987053	2.1310981
H	-3.0622222	-0.9846499	2.1312834
H	-1.9513980	3.3429802	1.2429216
H	-0.7674974	2.1210448	1.7244277
H	-3.2690036	1.3565941	-1.5420221
H	-3.3644438	2.9425015	-0.7287528
H	-1.9891072	2.5423480	-1.7921589
H	-3.8149515	0.6371915	0.4420279
H	-3.1589039	1.4555572	1.8496975
H	0.3970008	-0.0527659	-2.7901756
H	-0.6002985	-0.0598388	-2.8732126
H	1.4144115	-0.0445366	-2.3871279

THF : tetrahydrofuran solvent molecule
13
Energy = -232.5925783441

O	-0.0015557	0.0014218	-1.2568307
C	0.1578025	1.1738437	-0.4164078
C	-0.1578691	-1.1728329	-0.4183286
C	0.2220753	-0.7358684	0.9960709
C	-0.2222255	0.7348547	0.9972812
H	1.2051482	1.5036723	-0.4640137
H	-0.4821435	1.9672156	-0.8154063
H	-1.2045003	-1.5048433	-0.4660472
H	0.4835715	-1.9641002	-0.8191326
H	-0.2703177	-1.3371085	1.7652312
H	1.3060902	-0.8054752	1.1400205
H	-1.3062581	0.8043259	1.1412623
H	0.2701820	1.3348946	1.7673744

TS5a^{2+} : H_2 exchange via L4 dimer **1^{2+}**•THF

107
Energy = -2977.229501668

Ca	-1.7533576	-0.0129145	-0.0544912
H	0.1105691	0.0093218	-1.2637408
N	-1.8480483	-2.5625570	-0.6921506
N	-2.5632893	-1.3676906	2.0013784
N	-4.2410638	0.7879089	0.7414283
N	-3.4246985	-0.3518488	-1.9732139
C	-2.5859500	-3.2764168	0.3778862
H	-2.4019288	-4.3602044	0.3123451
H	-3.6565970	-3.1332169	0.2140806
C	-2.1941067	-2.7887758	1.7671911
H	-2.6584485	-3.4383046	2.5259132
H	-1.1090196	-2.8543917	1.8907048
C	-4.0323265	-1.2501223	2.1773614
H	-4.3308135	-1.6578809	3.1559224
H	-4.5178179	-1.8680853	1.4190490
C	-4.5248216	0.1835946	2.0672178
H	-5.6042305	0.2084944	2.2865221
H	-4.0351506	0.8094201	2.8188960
C	-5.1690327	0.2466876	-0.2836057
H	-6.1547934	0.7288807	-0.1928820
H	-5.3245273	-0.8158362	-0.0851180
C	-4.6500302	0.4435829	-1.7023620
H	-5.4455903	0.1861815	-2.4190345
H	-4.3971810	1.4954029	-1.8644926
C	-3.7677047	-1.7917052	-2.0902835
H	-4.2830155	-1.9805213	-3.0449455
H	-4.4732135	-2.0441150	-1.2960141
C	-2.5448278	-2.6889685	-1.9958244
H	-2.8462871	-3.7321635	-2.1800936
H	-1.8225385	-2.4234169	-2.7726299
C	-0.5022147	-3.1495601	-0.8388582
H	0.0224077	-3.0989542	0.1165521
H	0.0522282	-2.5548533	-1.5655448
H	-0.5580534	-4.1976427	-1.1738977
C	-1.8803291	-0.8960540	3.2273393
H	-2.0883670	0.1635728	3.3908637
H	-0.8041203	-1.0284842	3.1001320
H	-2.2142525	-1.4605505	4.1111794
C	-4.4611170	2.2461728	0.8508976
H	-4.2243169	2.7329868	-0.0955536
H	-3.8091875	2.6510451	1.6264602
H	-5.5065855	2.4651925	1.1185989
C	-2.8102578	0.1068137	-3.2399746
H	-1.8482172	-0.3874791	-3.3896572
H	-2.6394617	1.1824994	-3.1893253
H	-3.4647050	-0.1060715	-4.0987053
Ca	1.8666454	-0.0591923	0.0173255
H	0.0751693	-0.1072639	1.2764078
N	3.1537101	1.1639839	1.8882690
N	3.3601235	1.6900084	-1.0709905
N	3.4120939	-1.2704813	-1.6298940
N	3.1999528	-1.8232137	1.3266338
C	4.2772830	1.8956688	1.2453455
H	4.7103988	2.6220045	1.9493883
H	5.0662629	1.1766586	1.0126935
C	3.8498026	2.6234590	-0.0233865
H	4.6912078	3.2258801	-0.3975507
H	3.0356552	3.3198865	0.1985991
C	4.5002314	0.9829758	-1.7093903
H	5.0389711	1.6652695	-2.3844874
H	5.2049462	0.6926400	-0.9264756
C	4.0546602	-0.2440279	-2.4925138
H	4.9200491	-0.6702746	-3.0219968
H	3.3237992	0.0403340	-3.2543495
C	4.4377986	-2.0081143	-0.8476219
H	4.9598302	-2.7336331	-1.4896022
H	5.1900160	-1.2911577	-0.5103429
C	3.8408407	-2.7411735	0.3471478
H	4.6265275	-3.3401631	0.8324152
H	3.0701923	-3.4411504	0.0113762
C	4.2358281	-1.0890806	2.1007566
H	4.7011178	-1.7592661	2.8395457
H	5.0272203	-0.7852516	1.4115541
C	3.6768574	0.1308839	2.8218425
H	4.4590785	0.5542040	3.4701566
H	2.8493197	-0.1632628	3.4731806
C	2.3206749	2.1179508	2.6562290
H	1.9137109	2.8781378	1.9871617
H	1.4840111	1.5839907	3.1099556
H	2.9114462	2.6142623	3.4408803
C	2.6066537	2.4450980	-2.0962533
H	2.1897954	1.7597699	-2.8364846
H	1.7809571	2.9693049	-1.6141529
H	3.2482536	3.1786496	-2.6065740
C	2.6702530	-2.2128421	-2.4982578
H	2.2633549	-3.0288020	-1.8985768
H	1.8453957	-1.6822131	-2.9805939
H	3.3274116	-2.6436645	-3.2680809
C	2.3720674	-2.6263343	2.2554966
H	1.8751808	-1.9738132	2.9753514
H	1.6046629	-3.1553970	1.6875633
H	2.9873426	-3.3576967	2.8010268
O	-1.6795590	2.4540917	-0.4361659
C	-1.3390581	3.3869711	0.6302620
C	-0.1855440	4.2015146	0.0747429
C	-0.6086178	4.3745884	-1.3911633
C	-1.2728201	3.0334204	-1.7179243
H	-1.0823668	2.7876065	1.5072495
H	-2.2068735	4.0217196	0.8436956
H	0.7398540	3.6191999	0.1486180
H	-0.0470547	5.1504594	0.5975028
H	-1.3314748	5.1913007	-1.4759741
H	0.2256782	4.5924433	-2.0623774
H	-2.1644233	3.1512953	-2.3403123
H	-0.5882224	2.3161414	-2.1820777
H	0.1373612	-1.1094963	1.0811350
H	0.0429712	0.9273590	1.2201137

TS5^{2+} : H_2 exchange via L4 dimer **1**$^{2+}$

94

Energy = -2744.626240437

Ca	0.1319398	0.0308974	1.7607364
Ca	0.1285622	0.0341257	-1.7608576
H	1.3341265	0.2748690	-0.0010021
H	-0.8386600	-1.2086795	-0.0004439
N	0.2968007	-2.0956590	3.0670502
N	0.2911977	-2.0902143	-3.0710211
N	-0.2666073	2.0990269	3.1230947
N	-0.2721932	2.1046365	-3.1192440
N	2.1120326	0.2738424	3.2990689
N	2.1058860	0.2796523	-3.3020455
N	-2.1141682	-0.2806964	2.9169521
N	-2.1194938	-0.2754431	-2.9139199
C	0.9951794	2.4474695	3.8310641
C	0.9884408	2.4542339	-3.8286998
C	-1.0697250	-2.4739103	3.5143833
C	-1.0760735	-2.4678155	-3.5166792
C	1.7148046	1.2173168	4.3753584
C	1.7072505	1.2249965	-4.3761577
C	-1.8718947	-1.2704320	3.9992823
C	-1.8790923	-1.2635079	-3.9982145
C	0.8535630	-3.1574062	2.1984862
C	0.8497049	-3.1531544	-2.2050810
C	-0.6657485	3.2201643	2.2415567
C	-0.6695811	3.2243997	-2.2352056
C	1.1868529	-1.8577399	4.2331933
C	1.1792562	-1.8502591	-4.2382797
C	-1.3440150	1.7972345	4.1013795
C	-1.3511425	1.8042904	-4.0962558
C	2.4348678	-1.0644716	3.8603529
C	2.4278814	-1.0577416	-3.8659940
C	-2.5110120	1.0372585	3.4802144
C	-2.5172558	1.0434308	-3.4744651
C	3.2899848	0.7943917	2.5670547
C	3.2848795	0.7986659	-2.5706892
C	-3.1941023	-0.7633245	2.0266602
C	-3.1979553	-0.7592418	-2.0224996
H	0.7987169	3.1477941	4.6563241
H	0.7906368	3.1558969	-4.6525065
H	-1.0247218	-3.2279253	4.3142151
H	-1.0323267	-3.2203856	-4.3179473
H	1.6353388	2.9703295	3.1153297
H	1.6297414	2.9759619	-3.1131711
H	-1.5713430	-2.9389295	2.6614765
H	-1.5763125	-2.9344189	-2.6638058
H	0.1900294	-3.2957460	1.3415332
H	0.1874818	-3.2930489	-1.3473972
H	0.1252774	3.3955690	1.5072727
H	0.1229009	3.3984036	-1.5021266
H	0.6135170	-1.3189042	4.9920535
H	0.6047064	-1.3100047	-4.9952044
H	-0.9058651	1.2135049	4.9150276
H	-0.9142802	1.2217187	-4.9114256
H	1.0651413	0.6822331	5.0731120
H	1.0566033	0.6911680	-5.0739549
H	-1.3361894	-0.7675861	4.8087394
H	-1.3448182	-0.7593921	-4.8078295
H	0.9538009	-4.1095347	2.7399131
H	0.9499380	-4.1043070	-2.7482203
H	-0.8311480	4.1431623	2.8166734
H	-0.8360605	4.1483262	-2.8085250
H	1.4901546	-2.8146522	4.6843355
H	1.4816989	-2.8063919	-4.6916481
H	-1.7234770	2.7283832	4.5490216
H	-1.7312223	2.7360982	-4.5420069
H	2.5978262	1.5407223	4.9474708
H	2.5895034	1.5493118	-4.9489319
H	-2.8275243	-1.6183970	4.4200796
H	-2.8354654	-1.6107707	-4.4179067
H	1.8385478	-2.8600568	1.8299890
H	1.8349550	-2.8557677	-1.8373095
H	-1.5816932	2.9637861	1.7059765
H	-1.5845697	2.9670840	-1.6984836
H	3.0598088	1.7692434	2.1328017
H	3.0553551	1.7727555	-2.1344334
H	-2.9100704	-1.7166907	1.5783655
H	-2.9133763	-1.7133950	-1.5762224
H	3.0086997	-1.6027021	3.1007397
H	3.0028137	-1.5972620	-3.1081382
H	-2.9396545	1.6177996	2.6586014
H	-2.9448968	1.6226974	-2.6514071
H	3.0826503	-0.9733520	4.7452341
H	3.0743787	-0.9651737	-4.7516591
H	-3.3015431	0.9169332	4.2366843
H	-3.3088288	0.9241779	-4.2300207
H	3.5338109	0.1105969	1.7514863
H	3.5297286	0.1133889	-1.7566532
H	-3.3432084	-0.0339512	1.2279020
H	-3.3455407	-0.0310376	-1.2223500
H	4.1584712	0.8978674	3.2345540
H	4.1524939	0.9033169	-3.2391263
H	-4.1365548	-0.8928091	2.5794323
H	-4.1414102	-0.8876448	-2.5738408
H	-1.1846633	-0.2246479	0.0009176
H	-1.2550870	0.8029220	0.0020247

TS6^{2+} : H_2 exchange via L5 dimer **2**$^{2+}$

116

Energy = -3091.399044713

C	3.5133353	-1.9979582	1.7984549
H	4.8870082	-0.6655355	2.7879882
N	2.8733628	-2.1944576	0.4750192
H	4.2156158	-2.8170512	2.0157787
H	2.7304047	-2.0492775	2.5587085
C	4.2489499	-0.6666881	1.8892495
Ca	1.8332531	0.1064749	-0.3571153
C	1.7850528	-3.1902012	0.5923080
C	3.8797326	-2.7024711	-0.4910124

N	3.3276571	0.4979487	1.9145160
H	4.9145595	-0.5478482	1.0268717
Ca	-1.8020583	-0.2181968	0.3115769
N	3.5181956	-0.9136275	-2.1782252
N	1.6309376	1.3123734	-2.7531068
N	2.8575085	2.4559179	-0.2540112
H	0.0105131	-1.0554559	-0.5261089
H	0.0317055	1.0899729	0.5564418
H	1.0046172	-2.7929935	1.2393614
H	2.1634203	-4.1413651	0.9989489
H	1.3419110	-3.3667779	-0.3889620
C	3.4962196	-2.3751448	-1.9228120
H	3.9943709	-3.7913367	-0.3797114
H	4.8487995	-2.2601276	-0.2493728
C	2.6162637	0.5403762	3.2110126
C	4.1125636	1.7599077	1.7873735
N	-1.8262166	-0.9360820	2.8626919
N	-3.6972619	-2.1458097	0.8710168
N	-2.7961110	-1.4460190	-1.9040892
N	-3.4300651	1.3551278	-0.9583089
N	-2.4415141	1.9204305	1.7934262
C	2.9109477	-0.6565557	-3.5077732
C	4.9174122	-0.4361043	-2.1805647
C	2.6359698	0.8215089	-3.7261828
C	1.7980068	2.7586901	-2.4856332
C	0.2841299	1.0760771	-3.3039352
C	3.0127539	3.0328726	-1.6114574
C	2.0120439	3.3498847	0.5648514
C	4.1951926	2.2752425	0.3598026
H	4.1729267	-2.8992958	-2.6166411
H	2.4800598	-2.7290369	-2.1271631
H	1.8907199	1.3560086	3.1956926
H	3.3191441	0.6914452	4.0451508
H	2.0751559	-0.3890588	3.3799994
H	3.6377583	2.5152747	2.4169167
H	5.1303686	1.6229177	2.1786691
C	-0.5721839	-1.6446564	3.1894828
C	-1.8847303	0.3246088	3.6410374
C	-2.9698890	-1.8114525	3.2169018
C	-3.2391905	-2.8162562	2.1117729
C	-3.6017253	-3.1097865	-0.2525002
C	-5.1049389	-1.7323722	1.0534307
C	-3.8506171	-2.4442384	-1.5938688
C	-3.3437202	-0.3656680	-2.7568056
C	-1.6982264	-2.1377169	-2.6125061
C	-4.2196389	0.5894053	-1.9562139
C	-2.8001291	2.5133517	-1.6325766
C	-4.3209731	1.8192075	0.1341555
C	-2.8963034	1.3081977	3.0681852
C	-1.3592279	2.8909630	2.0699941
C	-3.5781508	2.6538937	1.1649920
H	1.9716100	-1.2183336	-3.5516635
H	3.5595319	-1.0412420	-4.3109990
H	5.5174746	-0.9746071	-2.9304205
H	5.3725542	-0.5835176	-1.2002338

H	4.9515314	0.6293560	-2.4100387
H	3.5554919	1.3985928	-3.6138611
H	2.2882795	0.9843399	-4.7571412
H	0.8867856	3.0943436	-1.9803658
H	1.8830410	3.3277299	-3.4253529
H	0.2003205	0.0359768	-3.6228345
H	0.0864873	1.7227890	-4.1736655
H	-0.4466478	1.2646969	-2.5155511
H	3.9078216	2.5909891	-2.0591608
H	3.1918823	4.1172803	-1.5528444
H	1.7276961	2.8530597	1.4936267
H	1.0981165	3.5726249	0.0142081
H	2.5322653	4.2913346	0.7990359
H	4.7565159	1.5792664	-0.2728993
H	4.7532955	3.2259696	0.3690630
H	0.2611858	-1.0618959	2.7930816
H	-0.4603785	-1.7799671	4.2762814
H	-0.5729480	-2.6300798	2.7191479
H	-2.1301535	0.1159808	4.6939622
H	-0.8836246	0.7634601	3.6216515
H	-2.7701267	-2.3375313	4.1626016
H	-3.8507077	-1.1877981	3.3837105
H	-3.9805366	-3.5576822	2.4493951
H	-2.3221611	-3.3645899	1.8738457
H	-2.5931448	-3.5369913	-0.2330056
H	-4.3131924	-3.9398870	-0.1126946
H	-5.7477300	-2.6026707	1.2586995
H	-5.1872544	-1.0362367	1.8898718
H	-5.4774018	-1.2311999	0.1599751
H	-4.8238715	-1.9501339	-1.5938248
H	-3.8915403	-3.2114459	-2.3811623
H	-2.4966181	0.1752945	-3.1893512
H	-3.9223176	-0.7792203	-3.5982837
H	-1.3456573	-2.9738482	-2.0051903
H	-2.0369990	-2.5187872	-3.5892448
H	-0.8620074	-1.4527219	-2.7398852
H	-4.9887874	0.0301442	-1.4167414
H	-4.7477492	1.2726202	-2.6380184
H	-2.3604684	2.1827509	-2.5736312
H	-3.5451787	3.2931323	-1.8532009
H	-2.0002689	2.9260231	-1.0156181
H	-4.7628933	0.9330822	0.6023083
H	-5.1512276	2.4213846	-0.2697067
H	-3.1011991	2.0954815	3.8113078
H	-3.8473626	0.7998736	2.8743911
H	-1.7039235	3.6907241	2.7430997
H	-0.5088326	2.3806815	2.5224204
H	-1.0310263	3.3277062	1.1258701
H	-3.1794526	3.5515169	0.6883319
H	-4.2879700	2.9948260	1.9313158
H	0.2757627	0.5273347	1.3747898
H	-0.2111563	1.4388454	-0.3861345

TS7^{+} : 1-hexene addition to L4 dimer **3**$^{+}$
111

Energy = -2980.129371377

Ca	1.9336855	-0.4799322	0.0365841
H	0.2981587	-1.2532525	-1.2634845
N	3.3992721	-2.5765240	-0.5815219
N	3.2893498	0.0724690	-2.0771601
N	3.4491860	1.5377845	0.5320613
N	3.5400527	-1.0926109	2.0085486
C	4.4145398	-2.0977070	-1.5491825
H	4.9121257	-2.9547503	-2.0323401
H	5.1862882	-1.5560262	-0.9960697
C	3.8215998	-1.1960114	-2.6249198
H	4.5860171	-1.0015437	-3.3951537
H	2.9892463	-1.7043321	-3.1192224
C	4.3788589	1.0136903	-1.7354895
H	4.7767997	1.4976042	-2.6423785
H	5.2021505	0.4416412	-1.2989196
C	3.9203322	2.0929913	-0.7587423
H	4.7389744	2.8148444	-0.6049205
H	3.0810859	2.6471583	-1.1892835
C	4.5806195	1.0958557	1.3766999
H	5.1031102	1.9641913	1.8106822
H	5.3026278	0.5799145	0.7380195
C	4.1322234	0.1724142	2.5056918
H	4.9885521	-0.0296770	3.1693042
H	3.3700959	0.6688684	3.1126888
C	4.5992273	-2.0149863	1.5393755
H	5.1159037	-2.4792028	2.3952629
H	5.3484080	-1.4277431	1.0022845
C	4.0542524	-3.1140704	0.6342660
H	4.8734047	-3.8040488	0.3729235
H	3.3047135	-3.7018029	1.1721421
C	2.5721498	-3.6301888	-1.2058361
H	1.9636147	-3.2009848	-2.0035055
H	1.8975041	-4.0427309	-0.4539277
H	3.1971914	-4.4441443	-1.6079631
C	2.3654652	0.6713322	-3.0622643
H	2.0339925	1.6478698	-2.7088811
H	1.4866511	0.0276993	-3.1523554
H	2.8525740	0.7962743	-4.0429272
C	2.6440111	2.5538447	1.2391588
H	2.2335198	2.1328121	2.1593778
H	1.8054401	2.8337755	0.5998476
H	3.2455068	3.4433537	1.4875612
C	2.7535202	-1.7210272	3.0905629
H	2.3519421	-2.6776575	2.7492127
H	1.9114597	-1.0710878	3.3385948
H	3.3726888	-1.9011628	3.9841734
Ca	-1.3093280	-0.1369079	-0.1575318
H	0.2779700	-0.4601632	1.4497607
N	-2.7279051	0.2192636	2.1014476
N	-1.7070528	-2.4503723	1.0199388
N	-2.9340020	-1.6247244	-1.6283070
N	-4.1683695	0.9553643	-0.5084006
C	-3.0626085	-1.1147030	2.6574248
H	-3.2598165	-1.0295567	3.7394695
H	-3.9961520	-1.4500162	2.1992119
C	-1.9757489	-2.1569915	2.4436193
H	-2.2568191	-3.0748375	2.9899324
H	-1.0288633	-1.7907249	2.8521607
C	-2.8170712	-3.1531343	0.3477849
H	-2.8305492	-4.2225837	0.6185294
H	-3.7632126	-2.7296863	0.6939930
C	-2.7070978	-3.0160576	-1.1671821
H	-3.4146590	-3.7058368	-1.6574673
H	-1.6998015	-3.2944479	-1.4892691
C	-4.3803456	-1.3168679	-1.5912882
H	-4.8924878	-1.8065101	-2.4370338
H	-4.7999681	-1.7565335	-0.6836091
C	-4.7009977	0.1705926	-1.6428186
H	-5.7984035	0.2803907	-1.7112673
H	-4.2852157	0.6109497	-2.5551925
C	-4.8085925	0.5853235	0.7688816
H	-5.8141959	1.0332380	0.8458883
H	-4.9468270	-0.4974655	0.7912480
C	-3.9698915	1.0156499	1.9653697
H	-4.5763191	0.9471461	2.8842127
H	-3.6772641	2.0645600	1.8574926
C	-1.8022076	0.9202280	3.0184068
H	-0.8601136	0.3696388	3.0609938
H	-1.5904136	1.9148323	2.6166862
H	-2.2426482	1.0260181	4.0241082
C	-0.4886069	-3.2732589	0.9483349
H	-0.2270137	-3.4536954	-0.0938820
H	0.3197014	-2.7040604	1.4142120
H	-0.6269569	-4.2354745	1.4719938
C	-2.4272382	-1.5022139	-3.0126885
H	-2.6548287	-0.5094740	-3.4033279
H	-1.3409961	-1.6274003	-2.9932592
H	-2.8885543	-2.2580839	-3.6704674
C	-4.4470396	2.3777475	-0.7829026
H	-4.1012442	2.9970690	0.0469216
H	-3.9220508	2.6905907	-1.6879673
H	-5.5284430	2.5504877	-0.9173240
C	-1.2722055	1.6857186	-2.2686945
C	-0.4331406	2.2862271	-1.3453015
H	-2.3204072	1.5227769	-2.0408351
H	-0.8905006	1.2560248	-3.1888037
H	0.4372147	1.2128579	-0.3783362
H	0.5288486	2.6551097	-1.6924765
C	-1.0176658	3.0817410	-0.1923827
C	-1.4461364	4.5052481	-0.5821435
H	-0.2971205	3.1341178	0.6310898
H	-1.9007253	2.5543898	0.2029115
C	-1.9915698	5.2959526	0.6121152
H	-0.5823009	5.0352483	-1.0074096
H	-2.2019198	4.4555860	-1.3747176
C	-2.4472479	6.7090372	0.2358727
H	-2.8343846	4.7458046	1.0546703
H	-1.2196202	5.3512866	1.3919807
H	-2.8339746	7.2494587	1.1067710

H	-1.6152259	7.2895394	-0.1798178
H	-3.2405403	6.6748491	-0.5201899

TS8$^+$: hydrogenolysis of **C**$^+$ with H_2

113

Energy = -2981.333807819

Ca	1.8846064	0.0044404	-0.0040358
H	0.6279056	-1.4906469	-0.9307761
N	3.7416982	-1.8247799	-0.2088983
N	3.2676913	0.5243295	-2.1036844
N	2.9782034	2.3810893	0.2468759
N	3.4119253	0.0136448	2.1191985
C	4.7328021	-1.2825285	-1.1686082
H	5.4338623	-2.0738176	-1.4808537
H	5.3263018	-0.5202384	-0.6566044
C	4.0777712	-0.6814540	-2.4063595
H	4.8559706	-0.4484324	-3.1506990
H	3.4051507	-1.4118559	-2.8636944
C	4.1388032	1.7019921	-1.8760994
H	4.4923061	2.1121900	-2.8354143
H	5.0276781	1.3687197	-1.3342109
C	3.4367396	2.8117773	-1.0983443
H	4.1128659	3.6790806	-1.0237298
H	2.5468591	3.1412736	-1.6440025
C	4.1261630	2.2114998	1.1690623
H	4.5258502	3.1949659	1.4651262
H	4.9261848	1.6945670	0.6330345
C	3.7527458	1.4258025	2.4199613
H	4.5809433	1.4789836	3.1444898
H	2.8800136	1.8762233	2.9002420
C	4.6450869	-0.7720519	1.8739127
H	5.1430264	-1.0150984	2.8261346
H	5.3410571	-0.1475787	1.3086576
C	4.3671222	-2.0624133	1.1139813
H	5.3063943	-2.6304039	1.0119722
H	3.6773724	-2.6904857	1.6859411
C	3.2027536	-3.1033064	-0.7195478
H	2.6593333	-2.9350640	-1.6492187
H	2.4954433	-3.5053121	0.0072525
H	4.0110605	-3.8350117	-0.8828224
C	2.3458032	0.7698715	-3.2358755
H	1.8283786	1.7182105	-3.0912619
H	1.5975331	-0.0271912	-3.2638089
H	2.8950306	0.8047097	-4.1899390
C	2.0591198	3.4029585	0.7958178
H	1.6249960	3.0515278	1.7339702
H	1.2472885	3.5688205	0.0860782
H	2.5853368	4.3536308	0.9762083
C	2.6683855	-0.5539230	3.2646185
H	2.5020046	-1.6210454	3.1044161
H	1.6968263	-0.0585316	3.3386500
H	3.2277537	-0.4281675	4.2049373
Ca	-1.3828127	-0.7069684	-0.3430451
H	0.0760564	0.1298922	1.2281030
N	-3.2073911	-0.1868720	1.5930917
N	-1.3685055	-2.6153301	1.4694881
N	-2.0601841	-2.9985611	-1.4162885
N	-3.9225685	-0.5757835	-1.2958704
C	-3.3612085	-1.4244849	2.3911310
H	-3.8692979	-1.1991139	3.3440677
H	-4.0132900	-2.1097009	1.8440874
C	-2.0307817	-2.1013224	2.6870374
H	-2.1921148	-2.9104735	3.4201284
H	-1.3416732	-1.3809208	3.1368741
C	-2.0436599	-3.8192615	0.9435817
H	-1.8006010	-4.7033719	1.5571798
H	-3.1240345	-3.6723333	1.0192232
C	-1.6555842	-4.0905819	-0.5041117
H	-2.0880033	-5.0537311	-0.8247880
H	-0.5685892	-4.1801526	-0.5849547
C	-3.5160142	-3.0320890	-1.6693399
H	-3.7671015	-3.8433476	-2.3737687
H	-4.0239336	-3.2678816	-0.7302096
C	-4.0405338	-1.7180414	-2.2333305
H	-5.0903433	-1.8546143	-2.5455227
H	-3.4655403	-1.4452481	-3.1229427
C	-4.8911679	-0.6860801	-0.1846320
H	-5.9064653	-0.4171355	-0.5215230
H	-4.9347811	-1.7302530	0.1339117
C	-4.5060313	0.1985587	0.9952129
H	-5.3123439	0.1731434	1.7475226
H	-4.4110908	1.2375498	0.6648468
C	-2.7292690	0.9018016	2.4704205
H	-1.7074881	0.6898703	2.7909679
H	-2.7168323	1.8340254	1.9043599
H	-3.3862687	1.0238504	3.3479642
C	0.0345672	-2.9247219	1.7924567
H	0.5458394	-3.2768398	0.8965888
H	0.5114316	-1.9972763	2.1155784
H	0.1060593	-3.6878203	2.5868447
C	-1.3148498	-3.1454716	-2.6840915
H	-1.6152563	-2.3583774	-3.3766680
H	-0.2522676	-3.0089604	-2.4692438
H	-1.4988697	-4.1344166	-3.1382237
C	-4.1866523	0.6578383	-2.0643942
H	-4.1738216	1.5258522	-1.4020030
H	-3.3967588	0.7781651	-2.8092691
H	-5.1703918	0.6118712	-2.5607550
C	-0.2409692	1.7236620	-1.4108370
C	-1.2965775	2.3283662	-0.4860941
H	-0.2752428	2.2206985	-2.3931785
H	0.7650512	1.9493594	-1.0228291
H	-2.2450084	1.7572246	-0.5648979
H	-0.9675252	2.2048263	0.5528091
C	-1.6692186	3.7970652	-0.7431495
H	-0.7672802	4.4195462	-0.6547591
H	-1.3031487	-0.1841879	-2.5510722
H	-0.7429036	0.4936388	-2.0910064
H	-2.0116326	3.8997522	-1.7828063
C	-2.7496611	4.3261614	0.2054316

C	-3.1560492	5.7782061	-0.0682767
H	-2.3967259	4.2394936	1.2435264
H	-3.6416958	3.6848097	0.1306619
C	-4.2409526	6.2842673	0.8879699
H	-2.2678769	6.4200690	0.0099215
H	-3.5094795	5.8628841	-1.1049085
H	-4.5131809	7.3231540	0.6716780
H	-5.1489485	5.6745151	0.8065837
H	-3.8986665	6.2351120	1.9286868

TS9^{+} : H_2 exchange via cation **3**$^{+}$
95
Energy = -2745.314168700

Ca	-1.5671066	-0.0147210	-0.0224514
H	-0.0092544	-0.1473365	-1.6029667
N	-3.0500397	-1.1755055	-1.7612677
N	-3.1416512	-1.7161669	1.1974304
N	-3.0091057	1.2360818	1.7439664
N	-2.9098044	1.7655487	-1.2198754
C	-4.1708600	-1.8619473	-1.0840624
H	-4.6611798	-2.5741563	-1.7685115
H	-4.9221307	-1.1124268	-0.8203696
C	-3.7164177	-2.6117146	0.1642220
H	-4.5664924	-3.1840406	0.5710228
H	-2.9382893	-3.3331672	-0.0993029
C	-4.2102749	-0.9496946	1.8784322
H	-4.7633950	-1.5984047	2.5776038
H	-4.9270847	-0.6182004	1.1227998
C	-3.6669662	0.2549976	2.6376120
H	-4.4862413	0.7239705	3.2069726
H	-2.9187519	-0.0707668	3.3654466
C	-4.0163184	2.0334056	1.0066069
H	-4.4663265	2.7927516	1.6676003
H	-4.8248808	1.3630242	0.7020193
C	-3.4283652	2.7257933	-0.2186777
H	-4.1902502	3.3885277	-0.6608880
H	-2.5862282	3.3547537	0.0822100
C	-4.0022816	1.1279842	-1.9863103
H	-4.4168157	1.8282290	-2.7303914
H	-4.8125428	0.8894687	-1.2914019
C	-3.5387481	-0.1396823	-2.7003012
H	-4.3613680	-0.5252403	-3.3246314
H	-2.7081910	0.0970512	-3.3705717
C	-2.2050019	-2.1459423	-2.4891592
H	-1.8149139	-2.8882338	-1.7906459
H	-1.3493899	-1.6096275	-2.9044377
H	-2.7734302	-2.6530789	-3.2861790
C	-2.3967045	-2.5333206	2.1787213
H	-1.9575931	-1.8889144	2.9419031
H	-1.5877747	-3.0517857	1.6577766
H	-3.0556206	-3.2696016	2.6670114
C	-2.1319720	2.1176533	2.5418566
H	-1.6052582	2.8079181	1.8824196
H	-1.3778245	1.5041558	3.0401137
H	-2.7094789	2.6789296	3.2954337
C	-1.9606537	2.4511083	-2.1205779
H	-1.5234499	1.7311674	-2.8143512
H	-1.1492447	2.8561854	-1.5121544
H	-2.4540696	3.2592798	-2.6849174
Ca	1.5644069	-0.0492015	-0.0285262
H	0.0127818	1.5133316	0.4062049
N	3.0173441	1.1843117	1.7493992
N	2.9127592	1.7385570	-1.2084291
N	3.0425779	-1.1971512	-1.7815468
N	3.1404875	-1.7580639	1.1721792
C	4.0174041	1.9983194	1.0208590
H	4.4577742	2.7568946	1.6891256
H	4.8340738	1.3392361	0.7132963
C	3.4248079	2.6941069	-0.1997483
H	4.1809202	3.3667911	-0.6370932
H	2.5763240	3.3123837	0.1056910
C	4.0098014	1.1031859	-1.9700297
H	4.4373085	1.8091894	-2.7014006
H	4.8103909	0.8515385	-1.2684350
C	3.5481185	-0.1538815	-2.7034036
H	4.3760589	-0.5369630	-3.3220242
H	2.7277574	0.0950072	-3.3816915
C	4.1541539	-1.9101198	-1.1161028
H	4.6247560	-2.6281324	-1.8081637
H	4.9224484	-1.1769414	-0.8551158
C	3.6965769	-2.6572960	0.1327110
H	4.5412637	-3.2432036	0.5317685
H	2.9061966	-3.3664610	-0.1285021
C	4.2203819	-0.9996645	1.8433745
H	4.7861703	-1.6560132	2.5253190
H	4.9236039	-0.6570782	1.0799623
C	3.6858650	0.1942876	2.6250928
H	4.5112683	0.6579318	3.1898606
H	2.9473857	-0.1406419	3.3585028
C	2.1390661	2.0494651	2.5637790
H	1.6135985	2.7530071	1.9174591
H	1.3837456	1.4260831	3.0480806
H	2.7154245	2.5952161	3.3296166
C	1.9718185	2.4312134	-2.1122653
H	1.5356690	1.7164026	-2.8118992
H	1.1587976	2.8366512	-1.5062422
H	2.4721669	3.2397895	-2.6700473
C	2.1872930	-2.1472534	-2.5252102
H	1.7940822	-2.9003588	-1.8396631
H	1.3343508	-1.5969819	-2.9279759
H	2.7499004	-2.6432092	-3.3332794
C	2.3952621	-2.5678537	2.1585497
H	1.9624546	-1.9194438	2.9220395
H	1.5805796	-3.0783057	1.6391581
H	3.0503592	-3.3074633	2.6468733
H	0.0052288	-1.5485803	0.9016965
H	-0.0095551	-0.7844818	1.5454205
H	0.0322944	-2.2850922	0.0728382
